# Evolution and Competitive Struggles of *Lactiplantibacillus plantarum* under Different Oxygen Contents

**DOI:** 10.3390/ijms25168861

**Published:** 2024-08-14

**Authors:** Sojeong Heo, Eun Jin Jung, Mi-Kyung Park, Moon-Hee Sung, Do-Won Jeong

**Affiliations:** 1 Department of Food and Nutrition, Dongduk Women’s University, Seoul 02748, Republic of Korea; hsjeong325v@gmail.com; 2 Ministry of Food and Drug Safety, Cheongju 28159, Republic of Korea; alwalj@korea.kr; 3 School of Food Science and Biotechnology, Food and Bio-Industry Research Institute, Kyungpook National University, Daegu 41566, Republic of Korea; parkmik@knu.ac.kr; 4 KookminBio Corporation, Seoul 02826, Republic of Korea; smoonhee@kookmin.ac.kr

**Keywords:** *Lactiplantibacillus plantarum*, pan-genome, core-genome, nitrate metabolism

## Abstract

*Lactiplantibacillus* (*Lb.*) *plantarum* is known as a benign bacterium found in various habitats, including the intestines of animals and fermented foods. Since animal intestines lack oxygen, while fermented foods provide a limited or more oxygen environment, this study aimed to investigate whether there were genetic differences in the growth of *Lb. plantarum* under aerobic vs. anaerobic conditions. Genomic analysis of *Lb. plantarum* obtained from five sources—animals, dairy products, fermented meat, fermented vegetables, and humans—was conducted. The analysis included not only an examination of oxygen-utilizing genes but also a comparative pan-genomic analysis to investigate evolutionary relationships between genomes. The ancestral gene analysis of the evolutionary pathway classified Lb. plantarum into groups A and B, with group A further subdivided into A1 and A2. It was confirmed that group A1 does not possess the *narGHIJ* operon, which is necessary for energy production under limited oxygen conditions. Additionally, it was found that group A1 has experienced more gene acquisition and loss compared to groups A2 and B. Despite an initial assumption that there would be genetic distinctions based on the origin (aerobic or anaerobic conditions), it was observed that such differentiation could not be attributed to the origin. However, the evolutionary process indicated that the loss of genes related to nitrate metabolism was essential in anaerobic or limited oxygen conditions, contrary to the initial hypothesis.

## 1. Introduction

Oxygen levels could influence microbial growth in their habitats [1,2]. Facultative anaerobic bacteria can grow under aerobic and anaerobic environments and evolve an extraordinary metabolic diversity to grow in both conditions. Although the evolution of the respiratory system in facultative anaerobic bacteria has been intensively studied in recent years [3], conversion of respiratory capacities under different environments is currently unclear. A few studies have analyzed the respiratory system in *Escherichia coli* or *Salmonella enterica* in vivo [4,5].

Lactic acid bacteria (LAB) are known to be microaerophilic or facultative anaerobic bacteria [6]. Since they are not obligate anaerobes, surviving in oxygen-devoid environments is challenging for them. Nonetheless, LAB are also isolated from strictly anaerobic or low-oxygen environments, such as the human gut and the guts of animals [7]. Consequently, LAB are sometimes referred to as oxygen-tolerant bacteria [8], and there has been research on the respiratory pathways of LAB [9]. However, there is a lack of comparative research on the genes involved in oxygen utilization based on the oxygen levels of their environments of origin. 

*Lactiplantibacillus* (*Lb.*) *plantarum* belongs to lactic acid bacteria, well known to be a probiotic species. *Lb. plantarum* bacteria are facultative anaerobic Gram-positive bacteria detected from several niches, including the gastrointestinal tracts of mammals and a variety of fermented foods, such as sausage, cheese, and vegetables [10]. The human intestine, with a very low oxygen level, is predominantly an anaerobic environment of *Lb. plantarum*. However, *Lb. plantarum* bacteria in fermented food are exposed to a more aerobic environment. Although *Lb. plantarum* bacteria can grow under both aerobic and anaerobic environments because they are facultative anaerobic bacteria, *Lb. plantarum* might adapt to different oxygen pressures. Little is known about how *Lb. plantarum* can adapt to diverse natural environments with various oxygen contents. Especially, molecular mechanisms that underpin its adaptation to anaerobic environments are not yet well understood. Thus, the current study performed a comparative genomic analysis of 25 *Lb. plantarum* strains from different niches to understand the dynamics of their evolution under aerobic and anaerobic conditions. This study introduced interspecific comprehensive comparative genome analysis to shed light on the genetic background of *Lb. plantarum* species.

## 2. Results and Discussion

### 2.1. Genome Summary and General Features

As of 30 June 2023, complete genomes of 228 *Lb. plantarum* strains were published in NCBI GenBank. They were classified according to their origins. Although soybeans and vegetables are both plant-based foods, we examined them separately, considering soybeans are consumed for their seeds, while vegetables are consumed for their leaves, stems, and other parts. Strains with an unclear origin and strains with an origin having a number ≤ 10 were excluded, as they were assumed to have low reproducibility or an insufficient number of genomes to identify core genomes accurately. After exclusion, 168 strains remained. The number of genomes according to the frequency of origin was in the order of fermented vegetables, human, and dairy products (Table 1 and Supplementary Table S1). The average genome sequence length of 228 strains was 3.31 Mb. Strain Gmze16 from Kombucha exhibited the smallest genome (2.79 Mb), while strain DF from Drosophila melanogaster possessed the largest genome (3.70 Mb). *Lb. plantarum* strains displayed an average G + C content of 44.46%. Strain ATG-K2 from fermented kimchi showed the highest G + C content of 45.08%, while strain SPC-SNU-72-1 from kimchi showed the lowest G + C content of 44.00%. We checked the number of coding sequences (CDSs) predicted from 228 *Lb. plantarum* genome sequences. Each genome possessed an average of 3015.5 CDSs. Comparable numbers of CDSs were obtained for each genome (Table 1). Genome size, G + C content, and CDSs did not significantly show differences among origins at *p* < 0.05 via Scheffe’s test (Table 1). 

### 2.2. Comparative Analysis of Lb. plantarum

For comparative genome analysis of *Lb. plantarum* strains of different origins, we selected five origins and, for each origin, five genomes. These genomes were prioritized based on the strains we possessed for future experimental validation. Afterward, we selected strains from various countries and diverse origins (Table 2). In the comparative analysis list, *Lb. plantarum* KM2 isolated from a low-temperature aging beef in a previous study [11] was included. Genome sizes ranged from 2.98 Mb to 3.58 Mb, and G + C content ranged from 44.15% to 44.84%. Strain Q7, isolated from a dairy product, showed the highest G + C content (44.84%), while strain BLS41, isolated from fermented vegetable, showed the lowest G + C content (44.15%). 

To analyze genomic distances between strain KM2 and *Lb. plantarum* reference strains, dDDH (digital DNA-DNA hybridization) and average nucleotide identity (ANI) values were calculated (Table 3). As a result, ANI and dDDH values for pair-wise comparisons were in ranges of 98.59–99.96% and 88.4–100.0%, respectively. These results reveal that genetic divergence exists between *Lb. plantarum* strains (Table 3). 

**Figure 1:**
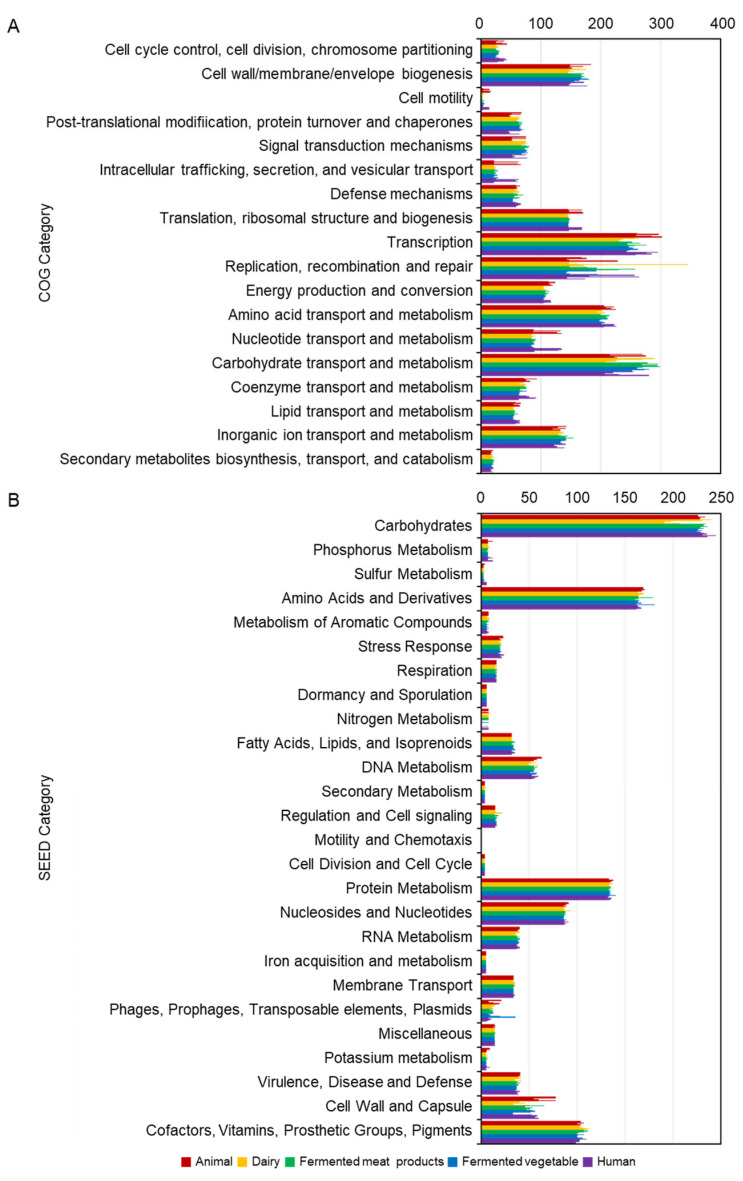
Comparison of functional categories of 25 *Lb. plantarum* genomes based on COG (**A**) and SEED (**B**). Genome sequences of 25 *Lb. plantarum* strains originated from five origins were uploaded to COG and SEED viewer servers independently. Functional roles of annotated genes were assigned and grouped in subsystem feature categories. The length of the colored bar represents the number of genes assigned to each category, and the color represents the origin.

The average number of CDSs predicted was 3027.3 for the 25 strains (Table 2). Predicted average numbers of coding sequences (CDSs) per genome using Clusters of Orthologous Gene (COG) functional categorization and SEED subsystem categorization were 2747.7 and 1105.3, respectively (Figure 1). Based on the COG functional categorization of genes, transcription was the most abundant category, followed by carbohydrate transport and metabolism (Figure 1A). In particular, in the case of strain NCIMB 700965, genes related to replication, recombination, and repair appeared to be the most abundant. However, this seemed to be a strain-specific result. The distribution of genes did not show any specific patterns according to origin.

Based on the SEED subsystem, the most abundant category was ‘carbohydrates’, followed by ‘amino acids and derivatives’ (Figure 1B). Contrary to expectations that the distribution of genes according to origin would be different, it was found that the distribution of COG and SEED according to origin was not significantly different. Interestingly, genes related to nitrogen metabolism were not detected from 11 strains (two strains, SRCM 210465 and E2, from animal; one strain, 8P-A3, from dairy product; two strains, R1-113 and MF1298, from fermented meat; four strains, LMT1-48, KC28, IDCC3501, and DSR_M2, from fermented vegetable; and two strains, LZ95 and BF_15, from human). The gene associated with nitrogen metabolism was included in the category of genes for energizing in oxygen-free conditions via nitrate to ammonium. Genomes containing genes involved in nitrogen metabolism had genes encoding nitrate reductase and transporter (Table S2). This gene involved the transfer of electrons in the order of the Fe-S cluster and molybdenum. It finally reduces them by converting nitrate to nitrite. Therefore, it is a gene necessary to transfer electrons under anaerobic conditions. However, these genes associated with nitrogen metabolism were also found to be strain-specific, regardless of origin. 

### 2.3. Pan-Genome and Core-Genome Analysis

**Figure 2:**
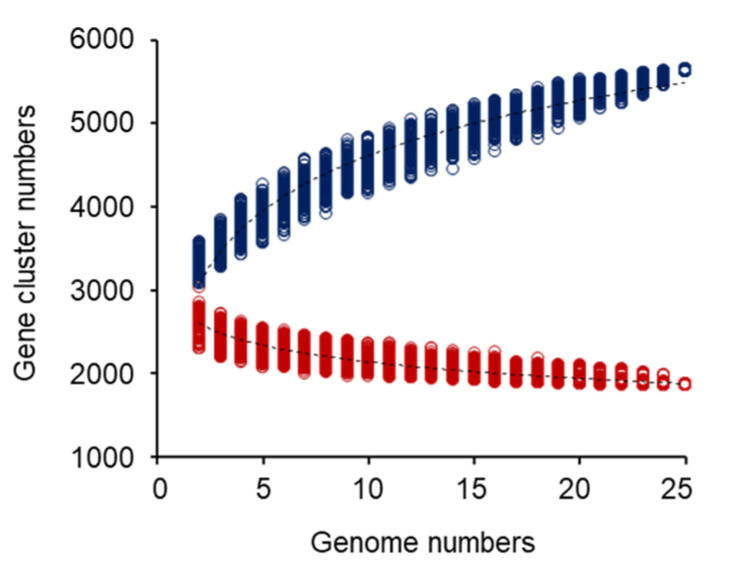
Sizes of core- and pan-genomes of 25 *Lb. plantarum* strains. Red curve (core genome) and blue curve (pan-genome) were fitted to decay function (937.967exp(−x / 11.490) + 1700.174) and Heap’s law function (2798.409 × 0.207), respectively. Each dot shows gene cluster number of an individual genome.

Comparative genomic analysis was conducted for 25 *Lb. plantarum* strains to determine their genomic diversity and evolutionary relationships. Estimation of the pan-genome of *Lb. plantarum* indicated that the gene pool should be increased with the sequential addition of each new genome (Figure 2). In this study, we predicted that the *Lb. plantarum* genome could hold at least 5634.3 ± 6.0 genes. Starting with 3338.6 ± 108.2 genes, there was a 3.5-fold increase from the first addition (217.5 genes) to the 25th addition (61.6 genes) as the growth rate gradually decreased (Figure 2). The above analyses confirmed that *Lb. plantarum* species possessed an open pan-genome that could increase in size with the addition of newly sequenced strains.

In contrast to the pan-genome, estimation of the core genome of *Lb. plantarum* indicated that genes shared by all strains decreased with each addition, finally reaching a plateau of around 1860.9 ± 4.7 genes (Figure 2). The decrease dropped from 2609.7 ± 121.0 genes to 2462.0 ± 119.9 genes upon the first addition. It dropped to 1860.9 ± 4.7 genes after the 25th addition. As a result, a final constant number of 1860.9 ± 4.7 shared genes was determined as the core genome size. The size of the *Lb. plantarum* core genome decreased as a function of the number of genomes included, while the size of the pan-genome increased. It is well known that core genes represent the essence of the species, while unique genes represent the diversity of the species [12]. We therefore conclude that 1860.9 ± 4.7 core genes of the 25 *Lb. plantarum* strains are essential components for these microorganisms to survive and thrive in nature. The average gene content for *Lb. plantarum* genomes was found to be 2747 ± 112 genes based on COG. The 25 *Lb. plantarum* strains shared 1860.9 ± 4.7 genes in their core genomes as determined by the Efficient Database framework for comparative Genome Analyses using BLAST score Ratios (EDGAR) (Figure 2). The core genome accounted for approximately 58.4–70.2% of genes in each genome. It was surprising that only 0.51–5.43% of the genes in the pan-genome of *Lb. plantarum* (based on 25 genome sequences) were present in only one lineage. In addition, most strain-specific genes were phage- or transposon-related, plasmid-encoded, or hypothetical (Table S3). The above genes have been frequently detected as strain-specific genes. The result of a low proportion of unique genes suggested that the diversity among *Lb. plantarum* strains was not high.

### 2.4. Shared and Unique Gene Analysis of Lb. plantarum

**Figure 3:**
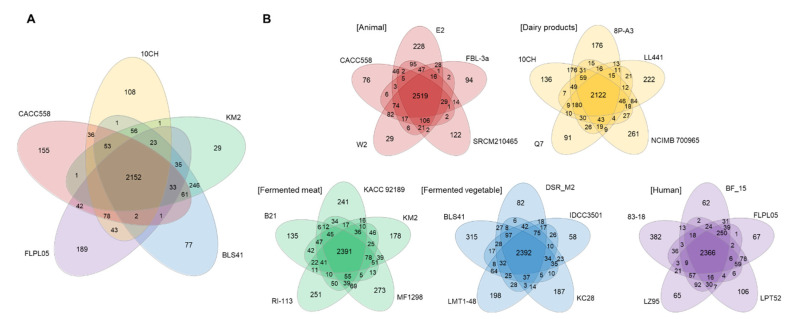
Venn diagrams of five *Lb. plantarum* genomes of different origins (**A**) or the same origins (**B**). Venn diagram generated using EDGAR. Overlapping regions represent *Lb. plantarum* CDSs shared between *Lb. plantarum* genomes. Numbers outside overlapping regions indicate numbers of CDSs in each genome without homologs in other sequenced *Lb. plantarum* genomes. Red, yellow, green, blue, and purple indicated animal, dairy, fermented meat, fermented vegetables, and human-derived strains.

To check unique genes according to origins, genes shared between genomes of the same origin were first analyzed (Figure 3). The number of core genes between five genomes of the same origins was identified as 2122–2519, which was about 74.5–84.1% of the total CDSs (Figure 3B). In terms of the number of core genes, strains derived from dairy products were found to share core genes with the lowest number (2122 genes). Many CDSs in the core genome were assigned via COG annotation to functions relating to metabolism and transcription. 

About 29 to 382 unique genes were identified for each strain, accounting for 1.00–12.78% of total CDSs. The most unique gene-rich strain was 83–18, which was derived from humans. However, as a result of checking the average unique genes by origin, the proportion of unique genes of human-derived strains was about 12.78%, which was higher than the proportion of average unique genes of 25 strains (average: 5.42%). The number or difference of common genes was not significant depending on the origin. Unique genes were either hypothetic protein genes or mobile elements such as transposase (Figure 3 and Table S4). We selected one strain from each origin (CACC 558 from animal, 10CH from dairy product, KM2 from fermented meat, BLS41 from fermented vegetable, and FLPL05 from human) and conducted a Venn diagram analysis. Results were similar to analysis results by origin (Figure 3A). Most of them were hypothetical proteins. Mobile elements such as recombinase and transposase, as unique genes of each strain, were analyzed. 

### 2.5. Gene Gain and Loss Analysis—Evolutionary Events

**Figure 4:**
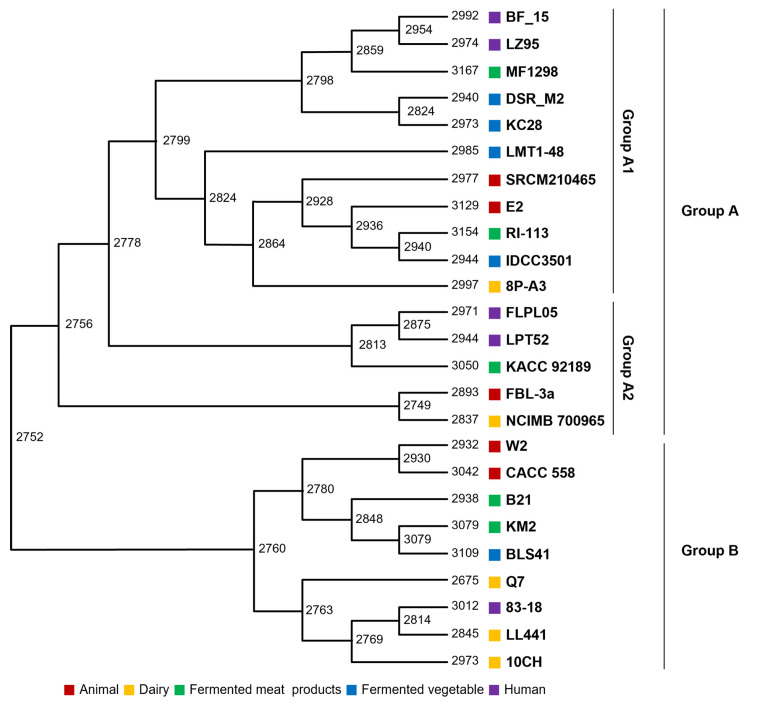
Analysis of ancestral genes in evolutionary path. Numbers adjacent to internal nodes indicate the number of estimated ancestral genes (protein-coding genes). Right panel indicates the number of CDSs of *Lb. plantarum* strains.

The evolution of bacteria frequently involves the acquisition or loss of genes. Gene acquisition may be a crucial ability for bacteria’s survival during their life. Loss of genes can occur when they are unnecessary for survival. In other words, evolution in bacteria concerning their survival is linked to the acquisition or loss of genes within their habitat. As mentioned in the introduction, patterns of gene gain or loss were examined based on the origin of the isolate. Evolutionary paths were analyzed using phylogenetic trees, and the number of genes for each gene group was generated from the most recent common ancestor (MCL) (Figure 4). The diversification of branches is associated with a large number of gene group coding sequences (CDSs) across *Lb. plantarum* as a whole. As shown in Figure 4, 2752 CDSs might have been inherited from the most recent common ancestor of *Lb. plantarum*. Additionally, except for strain Q7, an increase in the number of CDSs during the evolutionary process was observed (Figure 4). The number of strains and CDSs was increased due to evolutionary events. The increase in CDSs might be related to gene replication and horizontal gene transfer. It might also be related to the proliferation of repetitive proteins, such as gene fragmentation and transposases. 

**Figure 5:**
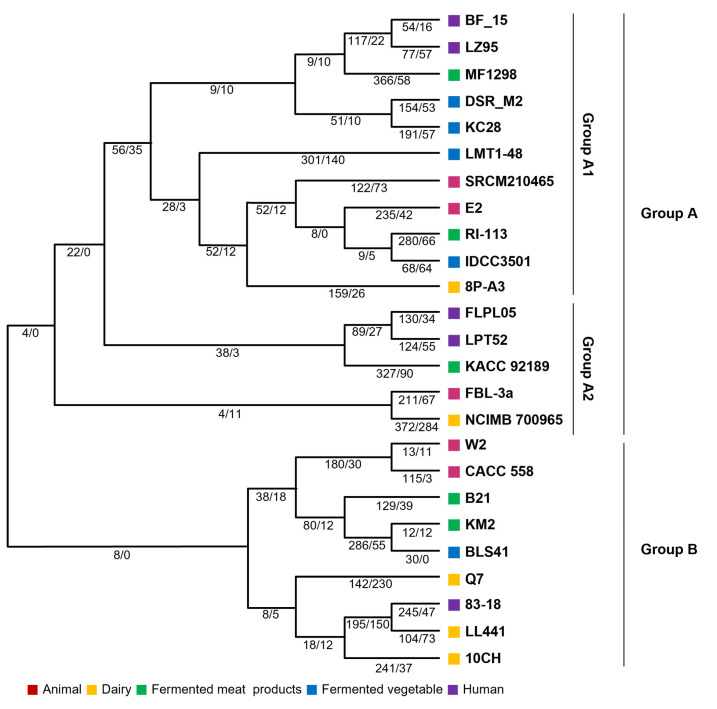
Minimal gene gain and loss events under the best-fit model (GD-FR-ML). Numbers on branches denote the minimum number of gains and losses in that order.

According to the results of the evolutionary path analysis, evolution could be divided into groups A and B. Group A appeared to have a greater number of genes. Group A had 2837–3167 CDSs, while group B had 2675–3109 CDSs. A lower gene acquisition in group B than in group A is evident in Figure 4. Specifically, strain Q7 showed a loss of more genes. Figure 5 illustrates the minimum gene gain and loss events at internal, external, and internal branches (Figure 5). Group A had higher numbers of expanded CDSs and branching points. Strain R1-113, which had the most branching points, possessed 2944 and 3154 CDSs, indicating a high gene gain. Strain RI-114 also showed a significant gene gain. Moreover, expansion was more frequent than loss in all branches, indicating that gene acquisition played a crucial role in dynamic evolution. Thus, it could be concluded that more dynamic evolution occurred in group A than in group B. 

Interestingly, among strains identified in group A, those corresponding to A1 did not possess all nitrogen metabolism-related genes mentioned in SEED analysis results (Figure 1B). Upon examining these genes, it was found that they were related to nitrate reductase (Table S2). The substance nitrate, to which the enzyme reacts, is one that can accept electrons under oxygen-limited conditions or anaerobic conditions [13]. In other words, the above results suggest that survival for those in group A might not be as easy as for other strains when oxygen is limited. However, we did not obtain results that distinguished gene gain or loss according to origin (Figure 4 and Figure 5).

### 2.6. Genomic Insights for Respiration System 

In the absence of oxygen, LAB can generate energy (ATP) through fermentation. However, in the presence of oxygen, they can generate ATP by the respiratory chain using NADH as an electron donor [9]. We assumed that types and numbers of genes associated with respiration or fermentation might be varied if *Lb. plantarum* could grow and evolve in environments with different oxygen concentrations. Therefore, the fermentation of *Lb. plantarum* with different origins and the presence or absence of genes related to the respiratory chain was analyzed.

#### 2.6.1. Hetero-Lactic Fermentation Pathway 

**Figure 6:**
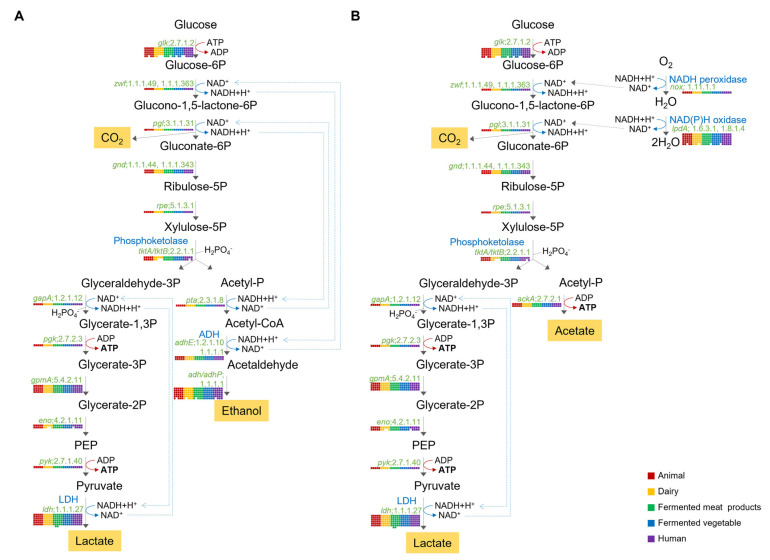
Predicted hetero-lactic fermentative pathways of 25 *Lb. plantarum* strains under anaerobic conditions (**A**) and aerobic conditions (**B**). Enzyme-encoding genes and E.C. number are displayed in green. Metabolites are shown in orange box. Gene possession was marked with a box of colors corresponding to each strain.

It is well known that *Lb. plantarum* can produce lactate, ethanol, carbon dioxide, and one ATP from glucose through the phosphoketolase pathway under anaerobic conditions (Figure 6A), and it can produce acetate instead of lactate using cofactor NAD+ generated from NADH and O_2_ by NADH oxidase to generate two ATP under aerobic conditions [14,15]. Results of this study also showed that the 25 *Lb. plantarum* strains possessed all genes required for the phosphoketolase pathway (Figure 6). Each strain had a total of five copies of a gene encoding glucokinase (*glk*; EC 2.7.1.2), which could produce glucose-6-phosphate using glucose as a substrate. However, some genes had only one copy in the genome of 25 strains, such as the gene encoding glucose-6-phosphate dehydrogenase (*zwf*; EC 1.1.1.49). Because human and animal intestines provide more anaerobic conditions than fermented foods, we assumed that *Lb. plantarum* from human and animal intestines had fewer genes using oxygen as a substrate, such as the gene encoding dihydrolipoyl dehydrogenase (*lpdA*; EC 1.6.3.1, 1.8.1.4), than *Lb. plantarum* from fermented foods. However, as shown in Figure 6B, 21 of 25 strains possessed six copies of the *lpdA* gene, and four strains had five copies of the *nox* gene. Especially, all five strains from human intestine had six copies of the *lpdA* gene. Thus, there was not much correlation depending on the source of isolation to determine oxygen availability based on the presence or absence of a gene related to the fermentation mechanism (Figure 6). 

#### 2.6.2. Respiratory Chain

**Figure 7:**
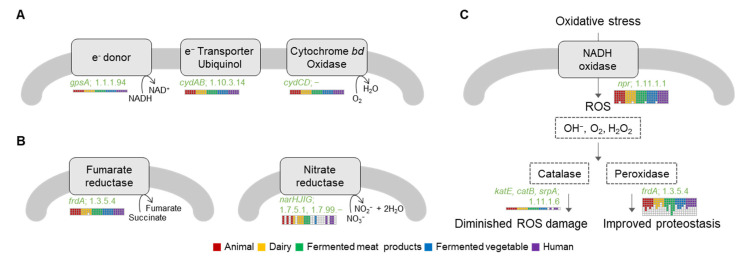
Predicted respiratory chain (**A**), nitrate reduction (**B**), and reactive oxidation species persistence system (**C**) of 25 *Lb. plantarum* strains under anaerobic conditions (**A**) and aerobic conditions (**B**). Enzyme-encoding genes and E.C. number are displayed in green. Gene possession was marked with a box of colors corresponding to each strain.

*Lb. plantarum* required three main membrane components for aerobic respiration: (a) NADH dehydrogenase as an electron donor, (b) quinone or quinol as an electron deliverer, and (c) cytochrome oxidase, a heme-dependent protein complex as an electron acceptor. Others include F0F1 ATP-producing enzymes for ATP production in protons released by the respiratory chain [16]. All 25 *Lb. plantarum* strains possessed genes required for aerobic respiration, including NADH dehydrogenase gene (*gpsA*; EC 1.1.1.94) as an electron donor, (b) ubiquinol oxidase gene (*cydAB*; EC 1.10.3.14) as an electron deliverer, and (c) cytochrome bd oxidase gene (*cydCD*) as an electron acceptor (Figure 7A). All strains had one copy of each gene in common, indicating that oxygen could be used as an energy source.

#### 2.6.3. Nitrate Reductase

Conversion from nitrate to ammonium is another mechanism for energizing in oxygen-free conditions. In the case of *E. coli*, formate is an electron donor that can eventually generate ammonia, protons, and energy by uptaking protons [13]. *Lb. plantarum* has many dehydrogenase genes encoding electron donors using NADH, formate, and lactate. Genes were not significantly different depending on origins or strains. Thus, the analysis was focused on electron acceptors that produced energy when oxygen was limited since the number of genes or the presence of genes related to energy production in the presence of oxygen showed no significant difference. Nitrate is a well-known electron acceptor in bacteria that can produce energy in limited oxygen conditions [13]. Sulfate, carbon dioxide, and fumarate are also electron acceptors [17]. *Lb. plantarum* strains used in the analysis possessed fumarate reductase and nitrate reductase as electron acceptors. However, they did not have any genes to accept electrons derived from sulfate or carbon dioxide (Figure 7B). A closer look revealed that, excluding strains derived from dairy products, genomes of *Lb. plantarum* contained four copies of the fumarate reductase gene (Figure 7B). In contrast, dairy product-derived strains had either four or three copies, fewer than copies possessed by strains from other origins (Figure 7B). Interestingly, not all *Lb. plantarum* strains possessed the *narGHIJ* operon associated with nitrate reduction. Among five strains of *Lb. plantarum* derived from fermented meat, three genomes contained the *narGHIJ* operon. Among strains from dairy and animal sources, two strains had the *narGHIJ* operon. Among strains derived from humans and fermented vegetables, only one strain had the *narGHIJ* operon (Figure 7B). Surprisingly, all strains harboring the *narGHIJ* operon were in the evolutionary path tree except for group A1 (Figure 4). NarGHIJ is a membrane-bound nitrate reductase responsible for anaerobic respiration in the presence of abundant nitrate. The catalytic subunit NarG is facing the cytoplasm. It contains the bis-MGD cofactor and an iron–sulfur cluster named FS0 [18]. NarH is an electron transfer unit containing four iron–sulfur centers. NarI contains a *b*-type cytochrome. It permits anchoring of the complex to the cytoplasmic membrane [19]. Nitrite (NO_2_^−^) generated by NarGHIJ from nitrate is toxic within the cell, requiring conversion to nitrogen for detoxification. For this process, the presence of NrfABCEDRFG or NapFDAGHBC is necessary. However, these two operons could not be found in the *Lb. plantarum* genome. Instead, it was observed that some strains possessed the gene *moaA* (Molybdopterin biosynthesis protein A), which could react with NO_2_ to produce a molybdenum-containing protein for detoxification (Table S5). Particularly noteworthy was that strains lacking *narGHIJ* were grouped into a distinct branch (Group A1) in the phylogenetic tree derived from evolutionary correlation (Figure 4). These results suggest that, during the evolution of *Lb. plantarum*, the necessity of *narGHIJ* for anaerobic respiration decreased, leading to the loss of this gene.

#### 2.6.4. Reactive Oxidation Species (ROS) Persistence System 

It is well known that *Lb. plantarum,* under anaerobic conditions, can produce lactate, ethanol, carbon dioxide, and one ATP from glucose through the phosphoketolase pathway. Under aerobic conditions, *Lb. plantarum* can produce acetate instead of lactate using cofactor NAD+ generated from NADH and O_2_ by NADH oxidase. When exposed to oxygen, LAB can generate reactive oxygen species (ROS) through NADH oxidase, causing toxicity to cells. The produced ROS can activate a system to eliminate them, involving enzymes such as superoxide dismutase (SOD), catalase, NADH oxidase, NADH peroxidase, and non-enzyme agents such as Mn^2+^, ascorbate, tocopherols, and glutathione. SOD can reduce ROS generated by oxygen by converting superoxide anion radicals into hydrogen peroxide. Catalase plays a role in producing water and oxygen from hydrogen peroxide. Although *Lb. plantarum* does not possess SOD, the presence of catalase and peroxidase genes suggests its ability to detoxify hydrogen peroxide produced due to exposure to oxygen (Figure 7C). Examining these genes based on their origins, strains possessing the *npr* gene, which can produce hydrogen peroxide upon exposure to oxygen, had 5–6 copies of this gene per genome. All strains had one copy of the catalase gene. However, for peroxidase, some strains had three or more copies, with the MF1298 strain derived from fermentation having up to eight copies. Strains with a high number of peroxidase genes were found to also possess genes producing molybdoprotein (Figure 7C). In conclusion, there was no significant difference between strains for possessed genes related to producing molybdoprotein.

*Lb. plantarum* is a species commonly detected in the microbial community of the gut or food. However, due to potential differences in the presence of oxygen between gut and food environments, it could be hypothesized that even though they belong to the same species, they might have undergone evolutionary changes. To interpret the evolution of *Lb. plantarum* isolated from five different sources, a genomic comparison was conducted. The initial hypothesis at the planning stage of the experiment suggested that *Lb. plantarum* isolated from human and animal intestines might have different genes responding to oxygen exposure, given their survival in conditions without oxygen. However, contrary to our hypothesis, a clear distinction based on the source of isolation could not be established. Despite selecting *Lb. plantarum* based on their source of isolation, it was challenging to determine whether strains survived in the intestines of animals or humans or whether the ingested *Lb. plantarum* were excreted through feces without colonizing the intestine. This ambiguity was considered a limitation in deriving experimental results. Nevertheless, results from comparative genomics indicated that genomes having the *narGHIJ* operon to allow survival in the absence of oxygen by utilizing nitrate had fewer coding DNA sequences (CDSs) on average than genomes that lacked the *narGHIJ* operon (Figure 4 and Figure 5). Additionally, the frequent branching of tree branches suggests that genomes lacking the nitrate operon might have undergone more evolution. In other words, *Lb. plantarum* belonging to Group A1, which does not possess the *narGHIJ* operon (Figure 4 and Figure 5), has likely adapted to oxygenated conditions by continuously shedding unnecessary nitrate-related genes. These results suggest that further investigation into the presence of the *narGHIJ* operon in *Lb. plantarum* strains from oxygen-limited environments, such as the gut, compared to those exposed to oxygen could provide a clearer understanding of their evolutionary pathways. Beyond its evolutionary implications, the *narGHIJ* operon could also serve as a biomarker for selecting probiotic strains in industrial applications. Probiotics intended to influence the gut microbiota must be able to colonize the gut; therefore, a strain that possesses the *narGHIJ* operon, allowing for growth in oxygen-limited environments, and exhibits health benefits would be an effective probiotic.

## 3. Materials and Methods

### 3.1. Datasets

For comparative genomic analysis, genome sequence data of *Lb. plantarum* were obtained from the NCBI database accessed on 30 June 2023 (http://ncbi.nlm.nih.gov/genomes) (Table 1). Genome sequence data for a total of 25 *Lb. plantarum* with five different origins (5 strains from each origin) were retrieved: animal (CACC 558, E2, FBL-3a, SRCM210465, and W2), dairy products (10CH, 8P-A3, LL441, NCIMB 700965, and Q7), fermented meat (B21, KACC 92189, KM2, MF1298, and RI-113), fermented vegetables (BLS41, DSR_M2, IDCC3501, KC28, and LMT1-48), and human (83-18, BF_15, FLPL05, LPT52, and LZ95) (Table 2). Genome sequences were uploaded to the Rapid Annotations using Subsystem Technology (RAST) server for SEED-based automated annotation, whole-genome sequence-based comparative analysis, and Kyoto Encyclopedia of Genes and Genomes metabolic pathway analysis [20] to annotated functions of genes. 

### 3.2. Genome Similarity Measures 

ANI, which provides a robust measure of genetic distance among bacterial genomes, was used for the comparative analysis of the conserved genes of genomes [21]. Genetic distances were calculated using estimated DNA–DNA hybridization values generated using the Genome-to-Genome Distance Calculator v. 3.0 with formula 2 (DSMZ, Braunschweig, Germany) [22]. 

### 3.3. Comparative Genome

EDGAR was used for core genome, pan-genome, and singleton analyses [23]. Genomes of strain KM2, E2, NCIMB 700965, MF1298, BLS41, and 83-18 were used as reference genomes for Venn diagram construction for the results of genome analyses of five species: animal, dairy products, fermented meat, fermented vegetable, and human-derivate species, respectively. Comparative analyses at the protein level were performed for annotated genomes with an all-against-all comparison. The algorithm used was BLASTP. Data were normalized according to the best score [24]. The score ratio value indicating the quality of hit was calculated by dividing scores of further hits by the best hit [25]. Two genes were considered orthologous when a bidirectional best BLAST hit with a single score ratio value threshold of at least 32% was obtained for orthology estimation. 

### 3.4. Construction of Phylogenetic Tree

Phylogenetic relationships and associated divergence times of individual species were estimated using our orthogroup alignment via a StarBEAST2 template [26] in BEAST2 v2.6.7 [27] by PHYZEN (Seongnam, South Korea). Single-copy (1735) orthologs shared with publicly available genomes were inferred with Orthofinder v. 2.5.4. They were sub-sampled to simultaneously minimize systematic bias and increase the phylogenetic signal with the Gamma Site Model (Category = 4; JTT; Centre for Computational Evolusion, The University of Auckland, New Zealand).

### 3.5. Gene Gain and Loss

Gain events, loss events, and turnover rates of gene families by maximum likelihood were analyzed using the birth, death, and innovation model of BadiRate v. 1.35 [28] by PHYZEN. The two different branch models, a global-rates model and a free-rates model, were applied to our data. The goodness of fit of these models was assessed using likelihood ratios. Clustering yielded 3946 orthogroups.

### 3.6. Statistical Analysis

Genome information *t*-test and Scheffe’s multiple range test following one-way analysis of variance (ANOVA) were used to evaluate significant differences in ratios of each origin values of average at *p* < 0.05, respectively. All statistical analyses were performed using SPSS software v.27 (SPSS Inc., Chicago, IL, USA).

## Figures and Tables

**Table 1:** Genome number and information of *Lb. plantarum* according to origin.

Origin	No. of Genome	Average Size of Genome (Mb) ^ns^	Average G + C Content (%) ^ns^	Average Number of CDSs ^ns^
Animal	30	3.34 ± 0.14	44.44 ± 0.16	3061 ± 151
Dairy products	25	3.27 ± 0.14	44.50 ± 0.14	2982 ± 127
Fermented alcohol	4	3.33 ± 0.16	44.46 ± 0.19	3043 ± 169
Fermented fish	1	3.20 ± 0.00	44.59 ± 0.00	2858 ± 0
Fermented meat	10	3.39 ± 0.10	44.35 ± 0.11	3084 ± 92
Fermented soybean	7	3.33 ± 0.07	44.48 ± 0.14	3028 ± 60
Fermented vegetable	65	3.31 ± 0.13	44.46 ± 0.19	3025 ± 143
Human	38	3.31 ± 0.07	44.48 ± 0.11	3015 ± 77
Plant	9	3.30 ± 0.07	44.45 ± 0.08	2976 ± 95
Others	39	3.31 ± 0.10	44.46 ± 0.14	3013 ± 100

^ns^: Not significant at *p* < 0.05 using Scheffe’s test.

**Table 2:** General genomic information of 25 *Lb. plantarum* strains used in this study.

Strain	Genome Assembly	Country	Origin	Genome Size (Mb)	G + C Content (%)	No. of CDSs
CACC 558	GCA_010092485.1	Korea	Animal; Canine	3.35	44.43	3056
E2	GCA_026013765.1	China	Animal; *Larimichthys crocea*	3.48	44.19	3165
FBL-3a	GCA_003999275.1	China	Animal; Beef cattle	3.23	44.60	2918
SRCM210465	GCA_024969905.1	Korea	Animal; *Sus scrofa domesticus*	3.26	44.50	2994
W2	GCA_021560135.1	China	Animal; *Penaeus vannamei*	3.26	44.60	2944
10CH	GCA_002005385.2	UK	Dairy products; Cheese	3.31	44.50	2993
8P-A3	GCA_009762745.1	Russia	Dairy products; Probiotic preparation “*Lactobacterinum*”	3.33	44.38	3030
LL441	GCA_027474465.1	Spain	Dairy products; Traditional cheese	3.20	44.50	2913
NCIMB 700965	GCA_003611015.1	New Zealand	Dairy products; Cheese	3.22	44.54	2978
Q7	GCA_003999605.1	China	Dairy products; Yak fermented milk	2.98	44.84	2730
B21	GCA_000931425.2	Australia	Fermented meat; Vietnamese fermented sausage (nem chua)	3.31	44.45	3011
KACC 92189	GCA_003692595.1	Korea	Fermented meat; Starter	3.46	44.30	3133
KM2	GCA_016838645.1	Korea	Fermented meat; Ripening beef	3.42	44.28	3111
MF1298	GCA_001880185.2	Norway	Fermented meat; Fermented sausage	3.58	44.22	3243
RI-113	GCA_001990145.1	Switzerland	Fermented meat; Fermented salami	3.46	44.34	3188
BLS41	GCA_002116955.1	Korea	Fermented vegetable; Kimchi	3.48	44.15	3195
DSR_M2	GCA_003286955.1	Korea	Fermented vegetable; Kimchi	3.25	44.59	2971
IDCC3501	GCA_003428355.1	Korea	Fermented vegetable; Kimchi	3.24	44.50	2957
KC28	GCA_002948215.1	Korea	Fermented vegetable; Kimchi	3.29	44.50	3042
LMT1-48	GCA_003813125.1	Korea	Fermented vegetable; Kimchi	3.28	44.50	3032
83-18	GCA_009759825.1	Russia	Human; Human feces (woman, 60 years old)	3.36	44.39	3107
BF_15	GCA_025723165.1	China	Human; The feces of breast-fed infants	3.31	44.50	3016
FLPL05	GCA_027557615.1	China	Human; Homo sapiens-faces	3.30	44.60	2992
LPT52	GCA_023348525.1	Ireland	Human; Homo sapiens-faces	3.27	44.60	2958
LZ95	GCA_001484005.1	China	Human; Homo sapiens	3.32	44.49	3005

Abbreviations: CDS, coding sequence.

**Table 3:** Genome-to-genome distances of 25 *Lb. plantarum* strains used in this study with genome of strain KM2 as a reference genome.

Query Genome	Strain	ANI Value	dDDH Value	Distance	Probability that dDDH > 70%	G + C Difference
KM2	CACC 558	99.26	93.7	0.0081	96.88	0.19
	E2	98.96	91.8	0.0102	96.35	86.50
	FBL-3a	99.12	93.1	0.0087	96.74	0.36
	SRCM210465	99.06	93.4	0.0084	96.82	86.90
	W2	99.24	94.5	0.0071	97.09	90.30
	10CH	99.15	93.2	0.0086	96.76	0.04
	8P-A3	99.11	92.6	0.0092	96.60	0.11
	LL441	98.80	89.2	0.0130	95.52	86.10
	NCIMB 700965	98.59	88.4	0.0138	95.23	0.23
	Q7	99.07	92.5	0.0094	96.58	85.70
	B21	99.41	95.9	0.0056	97.41	0.09
	KACC 92189	99.17	93.9	0.0079	96.93	0.09
	MF1298	99.06	93.5	0.0083	96.83	0.04
	RI-113	98.97	92.6	0.0093	96.59	0.01
	BLS41	99.96	100	0.0000	98.30	0.01
	DSR_M2	99.10	93.1	0.0087	96.74	0.09
	IDCC3501	99.11	93.0	0.0089	96.70	88.60
	KC28	99.09	92.5	0.0094	96.57	0.05
	LMT1-48	98.98	91.0	0.0110	96.13	86.40
	83-18	98.86	91.4	0.0106	96.25	0.21
	BF_15	99.07	92.1	0.0098	96.46	90.50
	FLPL05	99.03	93.0	0.0089	96.69	86.90
	LPT52	99.06	92.8	0.0091	96.65	90.30
	LZ95	99.08	93.3	0.0085	96.77	0.02

Abbreviations: ANI, average nucleotide identity; dDDH, digital DNA-DNA hybridization.

## Data Availability

Data are contained within the article or Supplementary Materials.

## Supplementary Materials

The following supporting information can be downloaded at: https://www.mdpi.com/article/10.3390/ijms25168861/s1, Table S1: General genomic information of the 228 Lb. plantarum strains registered in NCBI GenBank until June 2023; Table S2: Comparison of nitrate-related genes from the genomes of 25 Lb. plantarum; Table S3: List of strain-specific genes derived from pan-genome analysis using 25 Lb. plantarum genomes; Table S4: List of strain-specific genes derived from the Venn diagram results; Table S5: Status of gene retention involved in strain-specific Molybdopterin biosynthesis from 25 Lb. plantarum genomes.
