# Supplementary material for: Evolution and Competitive Struggles of Lactiplantibacillus plantarum under Different Oxygen Contents
Source: Int J Mol Sci. 2024 Aug 14;25(16):8861. doi: 10.3390/ijms25168861 (PMC11354895; doi:10.3390/ijms25168861)
Supplement: Supplementary file 1 [file ijms-25-08861-s001.zip › ijms-3131373-supplementary.pdf]

Table S1. General genomic information of the 228 *Lb. plantarum* strains registered in NCBI GenBank until June 2023

Group	Strain	Genome assembly	Size (Mb)	G+C contents (%)	CDS	Origin
Animal	12	GCA_017301935.1	3.31	44.50	2977	Scophthalmus maximus
	41P	GCA_019399915.1	3.27	44.49	3009	Meat
	A8	GCA_021559915.1	3.22	44.52	2926	Animal droppings
	BDGP2	GCA_002290185.1	3.58	44.25	3328	Drosophila melanogaster Oregon-R modENCODE
	BRD_L15	GCA_026183415.1	3.24	44.53	2958	Stool
	CACC 558	GCA_010092485.1	3.35	44.43	3056	Canine
	DF	GCA_001704335.1	3.70	44.07	3456	Drosophila melanogaster
	dm	GCA_002220175.1	3.33	44.50	3064	Drosophila melanogaster gut
	DMC-S1	GCA_029855105.1	3.47	44.33	3212	Drosophila melanogaster
	DR7	GCA_003586485.1	3.23	44.55	2941	Bos taurus
	E2	GCA_026013765.1	3.48	44.19	3165	Larimichthys crocea
	FBL-3a	GCA_003999275.1	3.23	44.60	2918	Beef cattle
	HC-2	GCA_013458335.1	3.36	44.26	3090	Chaeturichthys stigmatias
	KP	GCA_001704315.1	3.69	44.08	3452	Drosophila melanogaster
	L75a	GCA_019321805.1	3.36	44.43	3039	Scylla serrata
	LB1-2	GCA_002906875.1	3.54	44.11	3263	Apis mellifera
	LZ206	GCA_001659745.1	3.26	44.50	2958	Bos taurus
	LZ227	GCA_001660025.1	3.43	44.34	3164	Bos taurus
	MA2	GCA_029854235.1	3.30	44.56	3006	Tibet
	P-8	GCA_000392485.2	3.25	44.55	2951	Weaned piglets
	plantarum	GCA_003076435.1	3.16	44.70	2881	Weaned piglets
	SRCM210459	GCA_024800605.1	3.26	44.50	2995	Sus scrofa domesticus
	SRCM210465	GCA_024969905.1	3.26	44.50	2994	Sus scrofa domesticus
	SRCM210576	GCA_024969715.1	3.26	44.50	2993	Bos taurus feces
	SRCM210579	GCA_024970125.1	3.26	44.50	2997	Bos taurus feces
	SRCM210580	GCA_024970145.1	3.26	44.50	3002	Bos taurus feces
	SRCM210797	GCA_024970165.1	3.23	44.60	2919	Bos taurus feces
	W2	GCA_021560135.1	3.26	44.60	2944	Penaeus vannamei
	Y42	GCA_026240755.1	3.32	44.59	3157	Fish
	Y44	GCA_007833595.1	3.31	44.51	3008	Turbot
Dairy products	2025	GCA_000466905.3	3.47	44.28	3174	Milk products
	022AE	GCA_021279005.2	3.23	44.50	2939	Fermented dairy sample
	10CH	GCA_002005385.2	3.31	44.50	2993	Cheese
	12_3	GCA_004028335.1	3.40	44.38	3148	Fermented milk
	13_3	GCA_004028315.1	3.27	44.53	2947	Fermented milk
	8P-A3	GCA_009762745.1	3.33	44.38	3030	Probiotic preparation "Lactobacterinum"
	ATCC 8014	GCA_002749655.1	3.31	44.43	3029	Azerbaijan cheeses
	K25	GCA_003020005.1	3.41	44.42	3078	Tibetan kefir
	KLDS1.0386	GCA_017576965.1	3.27	44.45	2966	Traditional fermented dairy products in Inner Mongolia
	KLDS1.0391	GCA_002028365.1	2.91	44.65	2691	Traditional fermented dairy products
	LL441	GCA_027474465.1	3.20	44.50	2913	Traditional cheese

Group	Strain	Genome assembly	Size (Mb)	G+C contents (%)	CDS	Origin
	Lp-6	GCA_024181705.1	3.48	44.36	3196	Kefir
	LP-F1	GCA_023207995.1	3.31	44.50	3006	Fermented milk
	M17	GCA_018588615.2	3.29	44.51	2976	Motal cheese
	M19	GCA_018588605.2	3.51	44.32	3217	Motal cheese
	M8	GCA_018588665.2	3.29	44.51	2976	Motal cheese
	MSD1	GCA_019469465.1	3.08	44.90	2790	Curd
	NCIMB 700965	GCA_003611015.1	3.22	44.54	2978	New Zealand cheese
	NCIMB700965.EF.A	GCA_004328745.1	3.22	44.54	2971	Cheese
	Q7	GCA_003999605.1	2.98	44.84	2730	Yak fermented milk
	SCB0151	GCA_022810685.1	3.21	44.50	2940	Kefir grain
	SHY 21-2	GCA_016415605.1	3.33	44.42	3014	Sichuan Red Original Yak Yogurt
	TK-P2A	GCA_015377525.1	3.21	44.67	2907	Probitic products
	YW11	GCA_004028295.1	3.25	44.56	2927	Fermented milk
	Z.6-1	GCA_023973045.1	3.33	44.38	3016	Dairy starter culture
Fermented alcohol	AR195	GCA_017351995.1	3.36	44.47	3053	Rice wine rice syrup
	JBE490	GCA_002109405.1	3.20	44.57	2939	Nuruk, Korean traditional beverage starter
	UNQLp11	GCA_004730965.1	3.53	44.20	3278	Pinot noir wine
	XJ25	GCA_019076805.1	3.21	44.61	2902	Wine
Fermented fish	LPL-1	GCA_002205775.2	3.20	44.59	2950	Fermented fish
Fermented meat	B21	GCA_000931425.2	3.31	44.45	3011	Vietnamese fermented sausage (nem chua)
	BCC9546	GCA_014084065.1	3.32	44.42	3009	Fermented pork (nham)
	JB-1	GCA_023370155.1	3.31	44.45	2980	Fermented sausage
	KACC 92189	GCA_003692595.1	3.46	44.30	3133	Fermented sausage starter
	KM2	GCA_016838645.1	3.42	44.28	3111	Repening beef
	MF1298	GCA_001880185.2	3.58	44.22	3243	Fermented sausage, Norway
	RI-113	GCA_001990145.1	3.46	44.34	3188	Fermented salami
	TMW 1.25	GCA_002117245.1	3.35	44.30	3085	Raw sausage
	TMW 1.277	GCA_002117265.1	3.40	44.22	3115	Raw sausage
	TMW 1.708	GCA_002117285.1	3.25	44.55	2962	Raw sausage
Fermented soybean	5-2	GCA_001278015.1	3.24	44.70	2965	Fermented soybean
	JBE245	GCA_001596095.1	3.26	44.50	2979	Meju, fermented soybean paste
	SRCM102737	GCA_009913975.1	3.38	44.41	3074	Soybean paste (Chonggugjang)
	TS12	GCA_001908455.1	3.43	44.28	3094	Stinky Tofu
	WLPL21	GCA_029814785.1	3.30	44.50	2996	fermented soybeans
	X7021	GCA_002943545.1	3.41	44.40	3105	Brine of stinky tofu
	ZDY2013	GCA_015693925.1	3.30	44.60	2986	Traditional Chinese fermented soybeans
Fermented vegetable	3-1	GCA_024137985.1	3.45	44.39	3187	Pickle
	AMT74419	GCA_012974545.1	3.23	44.60	2934	Kimchi
	ATG-K2	GCA_003597635.1	3.18	45.08	2892	Kimchi
	ATG-K6	GCA_003597595.1	3.26	44.50	2974	Kimchi
	ATG-K8	GCA_003597615.1	3.28	44.51	2988	Kimchi
	AWA2013	GCA_030297715.1	3.42	44.35	3275	Plant derived fermented beverage

Group	Strain	Genome assembly	Size (Mb)	G+C contents (%)	CDS	Origin
	AWA2045	GCA_030297735.1	3.30	44.46	3156	Plant derived fermented beverage
	b-2	GCA_003352125.1	3.21	44.60	2951	Pickle
	BC015	GCA_026689375.1	3.44	44.35	3158	Chinese sauerkraut
	BK-021	GCA_013487805.1	3.47	44.34	3232	Fermented onions
	BLS41	GCA_002116955.1	3.48	44.15	3195	Kimchi
	CAUH2	GCA_001617525.2	3.27	44.55	2997	Sichuan pickle vegetables
	CNEI-KCA5	GCA_013694305.1	3.21	44.50	2968	Fermented Okpei-Nsukka
	CYLB47	GCA_029537295.1	3.54	44.08	3155	Microbe sample from fermented mustard green
	DSM 20174	GCA_014131735.1	3.25	44.48	2966	Pickled cabbage
	DSR_M2	GCA_003286955.1	3.25	44.59	2971	Kimchi
	EM	GCA_004337615.1	3.65	44.14	3324	Kimchi
	Gmze16	GCA_028411375.1	2.79	45.00	2468	Kombucha
	GR0128	GCA_017068235.1	3.33	44.46	2970	Fu-Tsai (Fermented vegetable, food)
	GR0512	GCA_017068215.1	3.46	44.24	2647	Fu-Tsai (Fermented vegetable, food)
	HAC01	GCA_003143915.1	3.23	44.55	2913	White Kimchi (Baek Kimchi)
	HMX2	GCA_025144505.1	3.32	44.50	3051	Northeast sauerkraut
	HOM3201	GCA_029906425.1	3.36	44.20	3130	Homemade pickle
	HOM3204	GCA_024396815.1	3.30	44.50	3005	Homemade pickled cabbage
	IDCC3501	GCA_003428355.1	3.24	44.50	2957	Kimchi
	IYO2065	GCA_030297695.1	3.30	44.50	3098	Plant derived fermented beverage
	K259	GCA_002868775.1	3.37	44.50	3086	Kimchi
	KC28	GCA_002948215.1	3.29	44.50	3042	Fermented Kimchi
	KC3	GCA_002868755.1	3.33	44.59	3033	Kimchi
	KCCP11226	GCA_009720585.1	3.38	44.39	3110	Kimchi
	KF511	GCA_029543005.1	3.25	44.61	2945	Kimchi
	L55	GCA_026153115.1	3.38	44.39	3191	Kimchi
	LM1004	GCA_002895245.1	3.20	44.60	2940	Napa cabbage kimchi
	LMT1-48	GCA_003813125.1	3.28	44.50	3032	Kimchi
	LP2	GCA_002109425.1	3.28	44.50	3012	Pickles
	LpYC41	GCA_024442115.1	3.30	44.61	3006	Pickle
	LQ80	GCA_003097595.1	3.45	44.38	3159	Pig feed from feed plant
	LRCC5314	GCA_017742875.1	3.25	44.50	2955	Kimchi
	LY-78	GCA_001715615.1	3.13	44.77	2847	Fermented chinese cabbage
	MGEL20154	GCA_025402835.1	3.25	44.48	2962	Kimchi
	MY04	GCA_029854335.1	3.48	44.40	3222	Fermented bamboo shoots
	NCU116	GCA_001672035.1	3.35	44.40	3084	Chinese pickle
	nF1	GCA_003325395.1	3.19	44.61	2910	Kimchi
	nF1-FD	GCA_003952885.1	3.20	44.61	2858	Kimchi
	P9	GCA_024181685.1	3.31	44.27	2974	Sour porridge
	PC518	GCA_016066915.1	3.43	44.21	3153	Sichuan pickle
	PC518	GCA_016598735.1	3.45	44.19	3175	Sichuan pickle
	PC520	GCA_002576835.1	3.45	44.35	3186	Chinese fermented food-pickles
	PMO08	GCA_014840995.1	3.32	44.39	3023	Kimchi
	S58	GCA_016775685.1	3.27	44.53	3011	Chinese pickle

Group	Strain	Genome assembly	Size (Mb)	G+C contents (%)	CDS	Origin
	SK151	GCA_003269405.1	3.23	44.60	2908	Kimchi
	SMB758	GCA_028768485.1	3.25	44.50	2973	Kimchi
	SPC-SNU 72-2	GCA_012109355.1	3.25	44.45	2972	Kimchi
	SPC-SNU-72-1	GCA_029637825.1	3.33	44.00	3068	Kimchi
	SRCM100995	GCA_009913675.1	3.48	44.21	3139	Pickled Green Chili Peppers
	SRCM101105	GCA_009913695.1	3.34	44.38	3018	Kimchi
	SRCM101167	GCA_009914095.1	3.41	44.38	3144	Water kimchi
	SRCM101222	GCA_009913835.1	3.36	44.43	3069	Young radish kimchi
	SRCM101511	GCA_009937825.1	3.27	44.41	2961	Diced Radish Kimchi
	SRCM101518	GCA_009913855.1	3.42	44.31	3140	Young radish kimchi
	SRCM101587	GCA_026976315.1	3.35	44.47	3134	Kimchi
	ST	GCA_022558425.1	3.32	44.40	3009	Tea(De'ang pickled tea)
	ST-III	GCA_000148815.2	3.31	44.50	2995	Chinese traditional pickle
	Zhang-LL	GCA_001581895.1	2.95	44.90	2700	Fermented rice
	ZS2058	GCA_001296095.1	3.20	44.70	2896	Sauerkraut
Human	202195	GCA_010586945.1	3.36	44.32	3060	Homo sapiens
	123-17	GCA_009759845.1	3.24	44.53	2922	Human feces (woman, 24 years old)
	83-18	GCA_009759825.1	3.36	44.39	3107	Human feces (woman, 60 years old)
	ATCC 202195	GCA_018351295.1	3.35	44.32	3040	Healthy infant feces
	BF_15	GCA_025723165.1	3.31	44.50	3016	The feces of breast-fed infants
	CGMCC 1.557	GCA_001272315.2	3.27	44.43	2996	Human gut
	DM083	GCA_024137845.1	3.20	44.60	2924	Homo sapiens
	FLPL05	GCA_027557615.1	3.30	44.60	2992	Homo sapiens-faces
	Heal19	GCA_013367715.1	3.37	44.40	3075	Human GI tract
	HFC8	GCA_001302645.1	3.41	44.33	3112	Faecal sample
	IRG1	GCA_004319665.1	3.38	44.41	3068	Korean infant feces
	LLY-606	GCA_006770505.1	3.26	44.47	2984	Homo sapiens
	LP3	GCA_002286275.1	3.33	44.39	3026	Homo sapiens
	LPC904	GCA_023348385.1	3.28	44.50	2985	Homo sapiens
	LPIMC513	GCA_023348465.1	3.21	44.60	2936	Homo sapiens-faces
	LPT52	GCA_023348525.1	3.27	44.60	2958	Homo sapiens-faces
	LS/07	GCA_011304595.2	3.43	44.46	3079	Homo sapiens
	LZ95	GCA_001484005.1	3.32	44.49	3005	Homo sapiens
	MWLp-12	GCA_028463965.1	3.27	44.46	3045	Homo sapiens
	pc-26	GCA_006770485.1	3.30	44.51	3006	Homo sapiens
	Q180	GCA_024732385.1	3.20	44.60	2943	Feces of healthy Korean adult
	SN35N	GCA_003966855.1	3.42	44.26	3152	Farm
	SRCM100438	GCA_009913615.1	3.22	44.60	2914	Infant faecal
	SRCM100440	GCA_009913635.1	3.22	44.60	2914	Infant faecal
	SRCM100442	GCA_009913655.1	3.22	44.60	2928	Infant faecal
	VHProbi E15	GCA_027558615.1	3.27	44.48	2962	Homo sapiens
	VHProbi O04	GCA_024758665.1	3.30	44.57	2996	Homo sapiens
	VHProbi O10	GCA_024758745.1	3.27	44.48	2965	Homo sapiens
	VHProbi P06	GCA_026016545.1	3.41	44.38	3155	Homo sapiens

Group	Strain	Genome assembly	Size (Mb)	G+C contents (%)	CDS	Origin
	VHProbi V22	GCA_028869445.1	3.39	44.37	3126	Homo sapiens
	WCFS1	GCA_000203855.3	3.35	44.45	3041	Human saliva
	WLPL04	GCA_001331925.2	3.14	44.70	2836	Homo sapiens
	ZFM4	GCA_003627355.1	3.30	44.46	3034	Homo sapiens
	ZFM518	GCA_029590535.1	3.33	44.47	3043	Infant feces
	ZFM55	GCA_003589725.1	3.41	44.35	3130	Homo sapiens
	ZFM9	GCA_003627335.1	3.43	44.28	3146	Homo sapiens
	ZJ316	GCA_000338115.2	3.30	44.45	2996	Homo sapiens
	ZW59	GCA_027920405.1	3.21	44.62	2940	GUT
Plant	B3	GCA_030061935.1	3.28	44.47	2925	Blueberry
	B4	GCA_030061955.1	3.31	44.46	2953	Blueberry
	CNEI-KCA4	GCA_013155145.1	3.33	44.30	3065	Plant
	LPgoji	GCA_030061915.1	3.24	44.48	2867	Wolfberry
	P9	GCA_021559675.1	3.39	44.46	3042	Plant
	SS6	GCA_030061985.1	3.23	44.49	2893	Mulberry
	WLPL01	GCA_029854315.1	3.24	44.52	2932	Plant
	X7022	GCA_011022295.1	3.24	44.51	2943	Plant
	YY-112	GCA_030253605.1	3.44	44.34	3164	Waxberry
Others	16	GCA_000412205.1	3.36	44.31	3038	-
	C410L1	GCA_001874125.1	3.39	44.42	3094	Pit mud
	CLP0611	GCA_002024845.1	3.23	44.54	2940	Environment
	CXG9	GCA_016812075.1	3.41	44.41	3100	Stinky xiancaigeng
	DOMLa	GCA_000604105.1	3.21	44.67	2897	-
	DW12	GCA_019425695.1	3.22	44.60	2904	Fermented food
	EN6	GCA_029542245.1	3.29	44.40	3041	Alfalfa
	G1	GCA_013256965.1	3.23	44.53	2947	Wheat germ
	GB-LP1	GCA_002220815.1	3.04	44.90	2736	Fermented food
	GR1184	GCA_019211765.1	3.31	44.49	3010	Food
	GR1186	GCA_021650875.1	3.31	44.49	2992	Food
	GR1187	GCA_019211785.1	3.33	44.46	3032	Food
	JDM1	GCA_000023085.1	3.20	44.70	2902	-
	Lp900	GCA_016894405.1	3.37	44.38	3028	Ogi (red sorghum)
	MNCW_1	GCA_023278325.1	3.29	44.50	3004	Fermented food
	NCIMB8826	GCA_017798305.1	3.34	44.46	3042	-
	SK156	GCA_014041895.1	3.23	44.60	2933	Fermented food
	SKO-001	GCA_005576935.1	3.38	44.30	3107	Angelica gigas Nakai root surface
	SRCM100434	GCA_002174195.1	3.22	44.60	2910	Food
	SRCM102022	GCA_002173655.1	3.33	44.40	3069	Food
	SRCM103295	GCA_004087995.1	3.45	44.31	3130	Food
	SRCM103297	GCA_004141755.1	3.33	44.38	3040	Food
	SRCM103300	GCA_004141875.1	3.39	44.31	3108	Food
	SRCM103303	GCA_004141895.1	3.30	44.25	3029	Food
	SRCM103311	GCA_004101325.1	3.44	44.31	3141	Food
	SRCM103357	GCA_004101505.1	3.54	44.29	3239	Food

Group	Strain	Genome assembly	Size (Mb)	G+C contents (%)	CDS	Origin
	SRCM103361	GCA_004101545.1	3.28	44.55	3016	Food
	SRCM103362	GCA_004101605.1	3.12	44.67	2767	Food
	SRCM103418	GCA_004101625.1	3.32	44.35	3012	Food
	SRCM103426	GCA_004101645.1	3.48	44.27	3165	Food
	SRCM103472	GCA_004103495.1	3.30	44.50	2983	Food
	SRCM103473	GCA_004103515.1	3.32	44.47	3003	Food
	TCI507	GCA_013305265.1	3.26	44.51	2969	Orange
	TMW 1.1308	GCA_009619495.1	3.33	44.51	3023	-
	TMW 1.1478	GCA_003345375.1	3.39	44.31	3117	Honey
	TMW 1.1623	GCA_002117305.1	3.33	44.37	3028	Fermented food
	VHProbi V38	GCA_023347215.1	3.20	44.59	2908	-
	ZDY04	GCA_029834415.1	3.30	44.60	2996	Vegetables
	ZW5	GCA_020881935.1	3.43	44.31	3124	Water

Table S2. Comparison of nitrate-related genes from the genomes of 25 *Lb. plantarum*

Strains	NarK/NasA family nitrate transporter	Nitrate reductase subunit alpha	Nitrate reductase subunit beta	Nitrate reductase molybdenum cofactor assembly chaperone	Respiratory nitrate reductase subunit gamma	ABC transporter substrate-binding protein	ABC transporter ATP-binding protein	Iron ABC transporter permease
Gene		1.7.99.4	1.7.99.4	1.7.99.4	1.7.99.4			
E.C. No.		*narG*	*narH*	*narJ*	*narI*			
CACC 558	GWD03_RS04055	GWD03_RS04135	GWD03_RS04140	GWD03_RS04145	GWD03_RS04150	GWD03_RS04020	GWD03_RS04025	GWD03_RS04030
E2	-	-	-	-	-	-	-	-
FBL-3a	EEJ46_RS06190	EEJ46_RS06110	EEJ46_RS06105	EEJ46_RS06100	EEJ46_RS06095	EEJ46_RS06230	EEJ46_RS06220	EEJ46_RS06215
SRCM210465	-	-	-	-	-	-	-	-
W2	L1599_RS05970	L1599_RS06050	L1599_RS06055	L1599_RS06060	L1599_RS06065	L1599_RS05935	L1599_RS05940	L1599_RS05945
10CH	B0667_RS06520	B0667_RS06600	B0667_RS06605	B0667_RS06610	B0667_RS06615	B0667_RS06485	B0667_RS06490	B0667_RS06495
8P-A3	-	-	-	-	-	-	-	-
LL441	O4Z47_RS06335	O4Z47_RS06415	O4Z47_RS06420	O4Z47_RS06425	O4Z47_RS06430	O4Z47_RS06300	O4Z47_RS06305	O4Z47_RS06310
NCIMB 700965	CO218_RS13920	CO218_RS14000	CO218_RS14005	CO218_RS14010	CO218_RS14015	CO218_RS13885	CO218_RS13890	CO218_RS13895
Q7	B1H25_RS08670	B1H25_RS08590	B1H25_RS08585	B1H25_RS08580	B1H25_RS08575	B1H25_RS08705	B1H25_RS08700	B1H25_RS08695
B21	SH83_RS06280	SH83_RS06360	SH83_RS06365	SH83_RS06370	SH83_RS06375	SH83_RS06245	SH83_RS06250	SH83_RS06255
KACC 92189	COO33_RS01085	COO33_RS01005	COO33_RS01000	COO33_RS00995	COO33_RS00990	COO33_RS01120	COO33_RS01115	COO33_RS01110
KM2	JQC82_RS06355	JQC82_RS06435	JQC82_RS06440	JQC82_RS06445	JQC82_RS06450	JQC82_RS06320	JQC82_RS06325	JQC82_RS06330
MF1298	-	-	-	-	-	-	-	-
RI-113	-	-	-	-	-	-	-	-
BLS41	BOQ65_RS00550	BOQ65_RS00630	BOQ65_RS00635	BOQ65_RS00640	BOQ65_RS00645	BOQ65_RS00515	BOQ65_RS00520	BOQ65_RS00525
DSR_M2	-	-	-	-	-	-	-	-
IDCC3501	-	-	-	-	-	-	-	-
KC28	-	-	-	-	-	-	-	-
LMT1-48	-	-	-	-	-	-	-	-
83-18	GPK32_RS07390	GPK32_RS07470	GPK32_RS07475	GPK32_RS07480	GPK32_RS07485	GPK32_RS07350	GPK32_RS07360	GPK32_RS07365
BF_15	-	-	-	-	-	-	-	-
FLPL05	GJS00_RS06135	GJS00_RS06215	GJS00_RS06220	GJS00_RS06225	GJS00_RS06230	GJS00_RS06100	GJS00_RS06105	GJS00_RS06110
LPT52	L0056_RS06315	L0056_RS06395	L0056_RS06400	L0056_RS06405	L0056_RS06410	L0056_RS06280	L0056_RS06285	L0056_RS06290
LZ95	-	-	-	-	-	-	-	-

Table S3. List of strain-specific genes derived from pan-genome analysis using 25 *Lb. plantarum* genomes

Strain	Product	Gene locus
CACC 558	Hypothetical protein	GWD03_RS08220
	AAA family ATPase	GWD03_RS08460
	Hypothetical protein	GWD03_RS08490
	Hypothetical protein	GWD03_RS08495
	Hypothetical protein	GWD03_RS08500
	Helix-turn-helix domain-containing protein	GWD03_RS12930
	DUF3800 domain-containing protein	GWD03_RS13345
	Hypothetical protein	GWD03_RS13420
	Hypothetical protein	GWD03_RS14900
	Hypothetical protein	GWD03_RS15195
	Y-family DNA polymerase	GWD03_RS15200
	Hypothetical protein	GWD03_RS15205
	Recombinase family protein	GWD03_RS15420
	IS30 family transposase	GWD03_RS15475
	Hypothetical protein	GWD03_RS15480
	SpaA isopeptide-forming pilin-related protein	GWD03_RS15500
E2	Helix-turn-helix domain-containing protein	OLJ37_00805
	Hypothetical protein	OLJ37_02270
	Hypothetical protein	OLJ37_02275
	DUF3021 domain-containing protein	OLJ37_02315
	LytTR family transcriptional regulator	OLJ37_02325
	Hypothetical protein	OLJ37_02330
	TIGR02328 family protein	OLJ37_02335
	YbgA family protein	OLJ37_02340
	Hypothetical protein	OLJ37_02345
	Hypothetical protein	OLJ37_02540
	DUF2971 domain-containing protein	OLJ37_02545
	Hypothetical protein	OLJ37_02560
	Phage tail tape measure protein	OLJ37_02565
	Hypothetical protein	OLJ37_02650
	Hypothetical protein	OLJ37_02655
	Hypothetical protein	OLJ37_02685
	IS30-like element ISLsa1 family transposase	OLJ37_02690
	Hypothetical protein	OLJ37_03115
	IS30-like element ISLsa1 family transposase	OLJ37_03920
	Hypothetical protein	OLJ37_03935
	Hypothetical protein	OLJ37_04020
	Hypothetical protein	OLJ37_05095
	Hypothetical protein	OLJ37_05695
	ATP-binding protein	OLJ37_05725
	Hypothetical protein	OLJ37_05775
	DUF1642 domain-containing protein	OLJ37_05795
	Hypothetical protein	OLJ37_05800
	ABC transporter permease subunit	OLJ37_05935
	Transposase	OLJ37_07490
	Hypothetical protein	OLJ37_07820
	Transposase	OLJ37_07835
	Type II secretion system GspH family protein	OLJ37_09215
	Endodeoxyribonuclease	OLJ37_09690
	Hypothetical protein	OLJ37_10485
	LamG domain-containing protein	OLJ37_11410
	TfoX/Sxy family protein	OLJ37_12850
	Transposase	OLJ37_13100
	Transposase	OLJ37_13715
	Hypothetical protein	OLJ37_14200
	AAA family ATPase	OLJ37_15580
	Primase C-terminal domain-containing protein	OLJ37_15585
	LysR family transcriptional regulator	OLJ37_15590

	FAD-dependent oxidoreductase	OLJ37_15600
	Anion permease	OLJ37_15605
	Hypothetical protein	OLJ37_15610
	DNA (cytosine-5-)-methyltransferase	OLJ37_15625
	ParB/RepB/Spo0J family partition protein	OLJ37_15720
	SNF2-related protein	OLJ37_15730
	MutH/Sau3AI family endonuclease	OLJ37_15735
	Very short patch repair endonuclease	OLJ37_15740
	Hypothetical protein	OLJ37_15745
	Hypothetical protein	OLJ37_15750
	Hydantoinase/oxoprolinase family protein	OLJ37_15770
	DUF917 domain-containing protein	OLJ37_15775
	Cytosine permease	OLJ37_15780
	PucR family transcriptional regulator ligand-binding domain-containing protein	OLJ37_15785
	LysR family transcriptional regulator	OLJ37_15790
	Hypothetical protein	OLJ37_15795
	Recombinase family protein	OLJ37_15800
	Amino acid permease	OLJ37_15825
	IS30-like element ISLpl1 family transposase	OLJ37_15830
	IS3 family transposase	OLJ37_15965
	ISL3 family transposase	OLJ37_15980
	DNA topoisomerase	OLJ37_16055
	Recombinase family protein	OLJ37_16080
	MarR family transcriptional regulator	OLJ37_16175
	KUP/HAK/KT family potassium transporter	OLJ37_16180
	Helix-turn-helix domain-containing protein	OLJ37_16185
	IS3 family transposase	OLJ37_16190
	Nicotinamide riboside transporter PnuC	OLJ37_16200
	IS30-like element ISLpl1 family transposase	OLJ37_16205
	IS256 family transposase	OLJ37_16215
	Hypothetical protein	OLJ37_16275
	VanZ family protein	OLJ37_16405
	IS256 family transposase	OLJ37_16440
	Glycosyltransferase family 2 protein	OLJ37_16445
	Hypothetical protein	OLJ37_16480
	Glycosyltransferase	OLJ37_16485
	Glycosyltransferase	OLJ37_16500
	Hypothetical protein	OLJ37_16510
	Hypothetical protein	OLJ37_16545
	Hypothetical protein	OLJ37_16605
FBL-3a	S8 family serine peptidase	EEJ46_RS02110
	AAA family ATPase	EEJ46_RS02115
	Hypothetical protein	EEJ46_RS02120
	Hypothetical protein	EEJ46_RS06900
	Hypothetical protein	EEJ46_RS06920
	Trypsin-like peptidase domain-containing protein	EEJ46_RS06925
	YitT family protein	EEJ46_RS07135
	M20/M25/M40 family metallo-hydrolase	EEJ46_RS07145
	Oligosaccharide flippase family protein	EEJ46_RS07150
	EpsG family protein	EEJ46_RS07605
	Glycosyltransferase	EEJ46_RS07615
	CDP-glycerol glycerophosphotransferase family protein	EEJ46_RS07620
	DUF1792 domain-containing protein	EEJ46_RS07625
	Hypothetical protein	EEJ46_RS07630
	Hypothetical protein	EEJ46_RS10955
	AAA family ATPase	EEJ46_RS12415
	Hypothetical protein	EEJ46_RS12420
	Hypothetical protein	EEJ46_RS15725
	Bacterial Ig-like domain-containing protein	EEJ46_RS15780
SRCM210465	Helix-turn-helix transcriptional regulator	NYR24_RS00310
	IS3 family transposase	NYR24_RS00320
	Zinc-binding dehydrogenase	NYR24_RS02085
	Hypothetical protein	NYR24_RS02150

	Hypothetical protein	NYR24_RS02975
	DUF916 domain-containing protein	NYR24_RS04745
	Hypothetical protein	NYR24_RS05900
	Hypothetical protein	NYR24_RS07980
	Hypothetical protein	NYR24_RS08100
	Oligosaccharide flippase family protein	NYR24_RS08155
	Hypothetical protein	NYR24_RS10135
	Stealth CR1 domain-containing protein	NYR24_RS10230
	Hypothetical protein	NYR24_RS10250
	Hypothetical protein	NYR24_RS11330
	Hypothetical protein	NYR24_RS11460
	Hypothetical protein	NYR24_RS12395
	Glycosyl hydrolase family 28-related protein	NYR24_RS12400
	Hypothetical protein	NYR24_RS12405
	Hypothetical protein	NYR24_RS12485
	Hypothetical protein	NYR24_RS12525
	Hypothetical protein	NYR24_RS12530
	Hypothetical protein	NYR24_RS12535
	HNH endonuclease	NYR24_RS12555
	Hypothetical protein	NYR24_RS13025
	ImmA/IrrE family metallo-endopeptidase	NYR24_RS13105
	GIY-YIG nuclease family protein	NYR24_RS13130
	Hypothetical protein	NYR24_RS13160
	GH-E family nuclease	NYR24_RS13600
	Hypothetical protein	NYR24_RS13625
W2	Hypothetical protein	L1599_RS00110
	Hypothetical protein	L1599_RS00625
	Hypothetical protein	L1599_RS03560
	Helix-turn-helix domain-containing protein	L1599_RS04765
	DMT family transporter	L1599_RS04770
	Hypothetical protein	L1599_RS05445
	Hypothetical protein	L1599_RS07120
	ISL3-like element ISP1 family transposase	L1599_RS07990
	Hypothetical protein	L1599_RS08030
	Hemolysin XhlA family protein	L1599_RS08590
	GH25 family lysozyme	L1599_RS10165
	Capsid protein	L1599_RS10170
	Hypothetical protein	L1599_RS10245
	Hypothetical protein	L1599_RS13205
	DUF1819 family protein	L1599_RS13575
10CH	DUF1788 domain-containing protein	B0667_RS03815
	BREX system P-loop protein BrxC	B0667_RS03820
	BREX-1 system adenine-specific DNA-methyltransferase PglX	B0667_RS03825
	BREX-1 system adenine-specific DNA-methyltransferase PglX	B0667_RS03830
	Hypothetical protein	B0667_RS03840
	Glycosyltransferase	B0667_RS04960
	EpsG family protein	B0667_RS05255
	Hypothetical protein	B0667_RS05265
	MarR family transcriptional regulator	B0667_RS05285
	YafY family transcriptional regulator	B0667_RS05530
	Alpha/beta hydrolase	B0667_RS05875
	YdeI/OmpD-associated family protein	B0667_RS05880
	DMT family transporter	B0667_RS05885
	ISL3 family transposase	B0667_RS05895
	Hypothetical protein	B0667_RS06210
	DUF4868 domain-containing protein	B0667_RS10755
	MerR family transcriptional regulator	B0667_RS10760
	Alpha/beta hydrolase	B0667_RS13110
	Aldo/keto reductase	B0667_RS13115
	NAD(P)H-binding protein	B0667_RS13120
	Hypothetical protein	B0667_RS13125
	Helix-turn-helix transcriptional regulator	B0667_RS13135
8P-A3	DUF2513 domain-containing protein	GPJ69_RS00150

	Hypothetical protein	GPJ69_RS02765
	Transcriptional regulator	GPJ69_RS02835
	Hypothetical protein	GPJ69_RS02845
	Hypothetical protein	GPJ69_RS02860
	Hypothetical protein	GPJ69_RS02990
	Hypothetical protein	GPJ69_RS03000
	Hypothetical protein	GPJ69_RS03005
	Hypothetical protein	GPJ69_RS03010
	Hypothetical protein	GPJ69_RS03020
	Restriction endonuclease subunit S	GPJ69_RS03025
	Restriction endonuclease subunit S	GPJ69_RS04110
	Restriction endonuclease subunit S	GPJ69_RS04125
	Glycosyltransferase family 4 protein	GPJ69_RS04130
	Glycosyltransferase	GPJ69_RS05210
	Toll/interleukin-1 receptor domain-containing protein	GPJ69_RS05215
	Hypothetical protein	GPJ69_RS05245
	AAA family ATPase	GPJ69_RS05250
	ATP-dependent helicase	GPJ69_RS05255
	TIGR04141 family sporadically distributed protein	GPJ69_RS05260
	Tyrosine-type recombinase/integrase	GPJ69_RS05265
	Hypothetical protein	GPJ69_RS05270
	Hypothetical protein	GPJ69_RS05275
	Hypothetical protein	GPJ69_RS05280
	Hypothetical protein	GPJ69_RS05285
	DUF1056 family protein	GPJ69_RS06095
	C40 family peptidase	GPJ69_RS06160
	ImmA/IrrE family metallo-endopeptidase	GPJ69_RS06265
	Hypothetical protein	GPJ69_RS10710
	Hypothetical protein	GPJ69_RS10715
	Host-nuclease inhibitor Gam family protein	GPJ69_RS10915
	IS5-like element ISLpl3 family transposase	GPJ69_RS10920
	PTS transporter subunit EIIB	GPJ69_RS11615
	DNA starvation/stationary phase protection protein	GPJ69_RS13995
	Hypothetical protein	GPJ69_RS15850
LL441	Hypothetical protein	O4Z47_RS00095
	Hypothetical protein	O4Z47_RS00635
	IS5-like element ISLpl3 family transposase	O4Z47_RS00640
	Hypothetical protein	O4Z47_RS00825
	IS3 family transposase	O4Z47_RS01295
	IS30 family transposase	O4Z47_RS01665
	IS3 family transposase	O4Z47_RS01960
	Helix-turn-helix domain-containing protein	O4Z47_RS02970
	IS3 family transposase	O4Z47_RS03055
	IS256 family transposase	O4Z47_RS03060
	Hypothetical protein	O4Z47_RS03095
	Hypothetical protein	O4Z47_RS03185
	Helix-turn-helix domain-containing protein	O4Z47_RS03365
	IS3 family transposase	O4Z47_RS03455
	KxYKxGKxW signal peptide domain-containing protein	O4Z47_RS03460
	Integrase core domain-containing protein	O4Z47_RS03980
	IS3 family transposase	O4Z47_RS04195
	Integrase core domain-containing protein	O4Z47_RS04200
	IS3 family transposase	O4Z47_RS04305
	IS3 family transposase	O4Z47_RS04310
	Hypothetical protein	O4Z47_RS04315
	IS3 family transposase	O4Z47_RS04435
	Phage tail protein	O4Z47_RS04445
	GDSL-type esterase/lipase family protein	O4Z47_RS04450
	Protein-export chaperone SecB	O4Z47_RS04465
	Hypothetical protein	O4Z47_RS04485
	Hypothetical protein	O4Z47_RS04490
	Hypothetical protein	O4Z47_RS04495
	Hypothetical protein	O4Z47_RS05565

Hypothetical protein	O4Z47_RS05580
Site-specific DNA-methyltransferase	O4Z47_RS05585
Hypothetical protein	O4Z47_RS05690
Hypothetical protein	O4Z47_RS05725
Hypothetical protein	O4Z47_RS05830
YfhO family protein	O4Z47_RS05855
IS256 family transposase	O4Z47_RS06125
Lytic polysaccharide monooxygenase	O4Z47_RS06545
IS1182 family transposase	O4Z47_RS07280
Hypothetical protein	O4Z47_RS07295
Hypothetical protein	O4Z47_RS08380
Hypothetical protein	O4Z47_RS08750
IS5-like element ISLpl3 family transposase	O4Z47_RS08840
Hypothetical protein	O4Z47_RS09035
Helix-turn-helix domain-containing protein	O4Z47_RS09145
IS3 family transposase	O4Z47_RS09400
IS256 family transposase	O4Z47_RS09435
IS3 family transposase	O4Z47_RS09710
KxYKxGKxW signal peptide domain-containing protein	O4Z47_RS09840
Hypothetical protein	O4Z47_RS09850
Hypothetical protein	O4Z47_RS09860
Hypothetical protein	O4Z47_RS09870
IS3 family transposase	O4Z47_RS09895
Helix-turn-helix domain-containing protein	O4Z47_RS09955
IS5-like element ISLpl3 family transposase	O4Z47_RS09960
IS5-like element ISLpl3 family transposase	O4Z47_RS10025
LasU family protein	O4Z47_RS10230
IS256 family transposase	O4Z47_RS10370
IS5-like element ISLpl3 family transposase	O4Z47_RS10375
IS5-like element ISLpl3 family transposase	O4Z47_RS10445
Hypothetical protein	O4Z47_RS10710
IS256 family transposase	O4Z47_RS10955
IS3 family transposase	O4Z47_RS11485
IS5-like element ISLpl3 family transposase	O4Z47_RS11730
IS30 family transposase	O4Z47_RS12030
LPXTG cell wall anchor domain-containing protein	O4Z47_RS12040
IS30 family transposase	O4Z47_RS12105
IS5-like element ISLpl3 family transposase	O4Z47_RS12180
Tyrosine-type recombinase/integrase	O4Z47_RS12260
Phage integrase SAM-like domain-containing protein	O4Z47_RS12265
Arm DNA-binding domain-containing protein	O4Z47_RS12275
Hypothetical protein	O4Z47_RS12280
IS5-like element ISLpl3 family transposase	O4Z47_RS12350
Hypothetical protein	O4Z47_RS12360
IS30 family transposase	O4Z47_RS12365
Hypothetical protein	O4Z47_RS12790
DUF3324 domain-containing protein	O4Z47_RS12885
IS5-like element ISLpl3 family transposase	O4Z47_RS13620
Hypothetical protein	O4Z47_RS13785
Glycoside hydrolase family 2 TIM barrel-domain containing protein	O4Z47_RS13800
IS5-like element ISLpl3 family transposase	O4Z47_RS13930
ABC transporter permease	O4Z47_RS14110
ABC transporter permease	O4Z47_RS14350
Peptidase domain-containing ABC transporter	O4Z47_RS14355
Type 2 lanthipeptide synthetase LanM family protein	O4Z47_RS14365
Plantaricin C family lantibiotic	O4Z47_RS14370
Type III toxin-antitoxin system ToxN/AbiQ family toxin	O4Z47_RS14375
AAA family ATPase	O4Z47_RS14380
ATP-dependent helicase	O4Z47_RS14385
DNA recombinase	O4Z47_RS14390
Class I SAM-dependent methyltransferase	O4Z47_RS14395
MFS transporter	O4Z47_RS14405
Hypothetical protein	O4Z47_RS14410

	Hypothetical protein	O4Z47_RS14415
	ThiF family adenylyltransferase	O4Z47_RS14420
	Recombinase family protein	O4Z47_RS14425
	NAD(P)/FAD-dependent oxidoreductase	O4Z47_RS14440
	Hypothetical protein	O4Z47_RS14445
	Type II toxin-antitoxin system YafQ family toxin	O4Z47_RS14450
	Type II toxin-antitoxin system prevent-host-death family antitoxin	O4Z47_RS14455
	Site-specific integrase	O4Z47_RS14460
	Hypothetical protein	O4Z47_RS14465
	MobQ family relaxase	O4Z47_RS14545
	Hypothetical protein	O4Z47_RS14555
	Putative holin-like toxin	O4Z47_RS14645
	Putative holin-like toxin	O4Z47_RS14650
	Beta-galactosidase small subunit	O4Z47_RS14680
	Beta-galactosidase C-terminal domain	O4Z47_RS14705
	IS3 family transposase	O4Z47_RS14720
	IS3 family transposase	O4Z47_RS14735
	Recombinase family protein	O4Z47_RS14740
	IS30 family transposase	O4Z47_RS14750
	IS256 family transposase	O4Z47_RS14835
	Anaerobic ribonucleoside-triphosphate reductase activating protein	O4Z47_RS14855
	Anaerobic ribonucleoside-triphosphate reductase	O4Z47_RS14910
	TetR/AcrR family transcriptional regulator	O4Z47_RS14915
	IS5 family transposase	O4Z47_RS14950
	DUF1906 domain-containing protein	O4Z47_RS14960
	Hypothetical protein	O4Z47_RS14965
	Metalloregulator ArsR/SmtB family transcription factor	O4Z47_RS14980
	Hypothetical protein	O4Z47_RS15080
	Site-specific integrase	O4Z47_RS15090
	Hypothetical protein	O4Z47_RS15305
	IS30-like element ISLpl1 family transposase	O4Z47_RS15330
NCIMB 700965	IS982-like element ISLpl4 family transposase	CO218_RS00175
	IS256 family transposase	CO218_RS00180
	IS5 family transposase	CO218_RS00215
	IS30 family transposase	CO218_RS00435
	IS30 family transposase	CO218_RS00910
	IS256 family transposase	CO218_RS00915
	IS66 family transposase	CO218_RS01100
	IS30-like element ISLpl1 family transposase	CO218_RS01195
	IS982 family transposase	CO218_RS01305
	Hypothetical protein	CO218_RS01310
	Hypothetical protein	CO218_RS01480
	Hypothetical protein	CO218_RS01485
	Hypothetical protein	CO218_RS01490
	Hypothetical protein	CO218_RS01615
	IS5-like element ISLpl3 family transposase	CO218_RS01625
	IS256 family transposase	CO218_RS01700
	Fic family protein	CO218_RS02030
	Hypothetical protein	CO218_RS03290
	Helix-turn-helix transcriptional regulator	CO218_RS03455
	ABC transporter ATP-binding protein	CO218_RS03690
	TetR/AcrR family transcriptional regulator	CO218_RS03835
	IS30-like element ISLpl1 family transposase	CO218_RS03840
	IS5-like element ISLpl3 family transposase	CO218_RS04000
	IS256 family transposase	CO218_RS04330
	IS256 family transposase	CO218_RS04450
	IS30 family transposase	CO218_RS04880
	IS30 family transposase	CO218_RS05070
	Hypothetical protein	CO218_RS05310
	Hypothetical protein	CO218_RS05355
	Hypothetical protein	CO218_RS05370
	IS256 family transposase	CO218_RS05380
	Hypothetical protein	CO218_RS05390

Hypothetical protein	CO218_RS05395
IS30-like element ISLpl1 family transposase	CO218_RS05405
IS3 family transposase	CO218_RS05415
Hypothetical protein	CO218_RS05425
Hypothetical protein	CO218_RS05430
IS30 family transposase	CO218_RS05435
IS30 family transposase	CO218_RS05600
ABC transporter ATP-binding protein	CO218_RS05675
IS30 family transposase	CO218_RS05750
IS256 family transposase	CO218_RS05760
IS30-like element ISLpl1 family transposase	CO218_RS05855
IS256 family transposase	CO218_RS05970
IS30 family transposase	CO218_RS06145
IS256 family transposase	CO218_RS06220
IS256 family transposase	CO218_RS06235
Helix-turn-helix domain-containing protein	CO218_RS06710
IS3 family transposase	CO218_RS06780
Helix-turn-helix domain-containing protein	CO218_RS06785
MFS transporter	CO218_RS07100
IS30 family transposase	CO218_RS07130
IS256 family transposase	CO218_RS07135
IS256 family transposase	CO218_RS07235
Hypothetical protein	CO218_RS07305
IS5 family transposase	CO218_RS07365
PASTA domain-containing protein	CO218_RS07520
IS5 family transposase	CO218_RS07535
IS3 family transposase	CO218_RS07540
IS3 family transposase	CO218_RS07735
IS30-like element ISLpl1 family transposase	CO218_RS07740
IS30 family transposase	CO218_RS07815
AAA family ATPase	CO218_RS08160
Helix-turn-helix transcriptional regulator	CO218_RS08170
IS30-like element ISLpl1 family transposase	CO218_RS08175
IS30-like element ISLpl1 family transposase	CO218_RS08185
IS30-like element ISLpl1 family transposase	CO218_RS08740
IS30 family transposase	CO218_RS09000
IS30-like element ISLpl1 family transposase	CO218_RS09120
Helix-turn-helix domain-containing protein	CO218_RS09355
IS30-like element ISLpl1 family transposase	CO218_RS09455
IS3-like element IS1163 family transposase	CO218_RS09590
IS256 family transposase	CO218_RS09625
IS30 family transposase	CO218_RS09930
IS5 family transposase	CO218_RS09940
IS30 family transposase	CO218_RS10015
ABC transporter ATP-binding protein	CO218_RS10845
IS256 family transposase	CO218_RS11170
DUF1700 domain-containing protein	CO218_RS11175
PadR family transcriptional regulator	CO218_RS11180
Hypothetical protein	CO218_RS11185
IS30 family transposase	CO218_RS11190
Hypothetical protein	CO218_RS11195
Hypothetical protein	CO218_RS11200
IS30-like element ISLpl1 family transposase	CO218_RS11205
GNAT family N-acetyltransferase	CO218_RS11540
IS30 family transposase	CO218_RS11680
IS30 family transposase	CO218_RS11695
IS3 family transposase	CO218_RS11710
IS30-like element ISLpl1 family transposase	CO218_RS11800
Hypothetical protein	CO218_RS12355
IS3 family transposase	CO218_RS12515
Helix-turn-helix domain-containing protein	CO218_RS12735
IS30-like element ISLpl1 family transposase	CO218_RS12740
Hypothetical protein	CO218_RS12775

	IS30-like element ISLpl1 family transposase	CO218_RS12805
	IS30 family transposase	CO218_RS13230
	IS982-like element ISLpl4 family transposase	CO218_RS13360
	TetR/AcrR family transcriptional regulator C-terminal domain-containing protein	CO218_RS13680
	NmrA family NAD(P)-binding protein	CO218_RS13735
	IS256 family transposase	CO218_RS13740
	IS30 family transposase	CO218_RS14215
	IS30 family transposase	CO218_RS14880
	Putative holin-like toxin	CO218_RS15000
	Class A sortase	CO218_RS15145
	Isopeptide-forming domain-containing fimbrial protein	CO218_RS15195
	IS30 family transposase	CO218_RS15200
	IS256 family transposase	CO218_RS15210
	Purine permease	CO218_RS15215
	IS66 family transposase	CO218_RS15275
	Universal stress protein	CO218_RS15280
	Nramp family divalent metal transporter	CO218_RS15350
	Hypothetical protein	CO218_RS15355
	Hypothetical protein	CO218_RS15370
	IS30 family transposase	CO218_RS15380
	ADP-ribosylglycohydrolase family protein	CO218_RS15425
	Cytosine permease	CO218_RS15450
	Ribokinase	CO218_RS15455
	Hypothetical protein	CO218_RS15460
	ABC transporter ATP-binding protein	CO218_RS15465
	ABC transporter permease	CO218_RS15485
	Hypothetical protein	CO218_RS15490
	IS5 family transposase	CO218_RS15550
	Hypothetical protein	CO218_RS15595
	ParA family protein	CO218_RS15620
	AAA family ATPase	CO218_RS15645
	ATP-dependent helicase	CO218_RS15705
	Putative holin-like toxin	CO218_RS15710
	Pyruvate oxidase	CO218_RS15730
	IS3 family transposase	CO218_RS15860
	Transposase	CO218_RS15870
	IS5 family transposase	CO218_RS15875
	Replication/maintenance protein RepL	CO218_RS15910
	IS5 family transposase	CO218_RS15930
	Hypothetical protein	CO218_RS15950
	Helix-turn-helix transcriptional regulator	CO218_RS15975
	Hypothetical protein	CO218_RS16000
	IS30 family transposase	CO218_RS16085
	CPBP family intramembrane metalloprotease	CO218_RS16090
	Hypothetical protein	CO218_RS16100
	Hypothetical protein	CO218_RS16125
	Hypothetical protein	CO218_RS16150
	Hypothetical protein	CO218_RS16160
	Hypothetical protein	CO218_RS16175
	Hypothetical protein	CO218_RS16185
	Hypothetical protein	CO218_RS16225
	Hypothetical protein	CO218_RS16235
	Hypothetical protein	CO218_RS16250
	Hypothetical protein	CO218_RS16280
	Hypothetical protein	CO218_RS16285
	Hypothetical protein	CO218_RS16290
Q7	Hypothetical protein	B1H25_RS00100
	IS5-like element ISLpl3 family transposase	B1H25_RS00105
	IS5-like element ISLpl3 family transposase	B1H25_RS00140
	NUDIX hydrolase	B1H25_RS00490
	IS5-like element ISLpl3 family transposase	B1H25_RS00645
	IS5-like element ISLpl3 family transposase	B1H25_RS00855
	Ltp family lipoprotein	B1H25_RS01810

	IS5-like element ISLpl3 family transposase	B1H25_RS02460
	IS5-like element ISLpl3 family transposase	B1H25_RS03575
	Helix-turn-helix transcriptional regulator	B1H25_RS03985
	Hypothetical protein	B1H25_RS04050
	Hypothetical protein	B1H25_RS04075
	Hypothetical protein	B1H25_RS04080
	Type I toxin-antitoxin system Fst family toxin	B1H25_RS04100
	Hypothetical protein	B1H25_RS04105
	ATP-binding protein	B1H25_RS04110
	IS5-like element ISLpl3 family transposase	B1H25_RS04365
	AAA family ATPase	B1H25_RS04370
	Hypothetical protein	B1H25_RS04375
	DUF2188 domain-containing protein	B1H25_RS04415
	ImmA/IrrE family metallo-endopeptidase	B1H25_RS04420
	Hypothetical protein	B1H25_RS04520
	Hypothetical protein	B1H25_RS04545
	SGNH/GDSL hydrolase family protein	B1H25_RS04610
	Hypothetical protein	B1H25_RS04615
	Hypothetical protein	B1H25_RS04620
	Hypothetical protein	B1H25_RS04625
	IS5-like element ISLpl3 family transposase	B1H25_RS06420
	IS30 family transposase	B1H25_RS07160
	IS30 family transposase	B1H25_RS07170
	IS30 family transposase	B1H25_RS07185
	IS5 family transposase	B1H25_RS09330
	C40 family peptidase	B1H25_RS12315
	C40 family peptidase	B1H25_RS13125
	Hypothetical protein	B1H25_RS13130
	IS256 family transposase	B1H25_RS13135
	Type I restriction endonuclease subunit R	B1H25_RS13985
	Tyrosine-type recombinase/integrase	B1H25_RS14325
	DUF3796 domain-containing protein	B1H25_RS14330
	Hypothetical protein	B1H25_RS14340
	Helix-turn-helix transcriptional regulator	B1H25_RS14345
	PedC/BrcD family bacteriocin maturation disulfide isomerase	B1H25_RS14355
	Pediocin PA-1 immunity protein	B1H25_RS14380
	Leucocin A/sakacin P family class II bacteriocin	B1H25_RS14385
	DUF2382 domain-containing protein	B1H25_RS14390
	Hypothetical protein	B1H25_RS14395
	Hypothetical protein	B1H25_RS14415
	Non-ribosomal peptide synthetase	B1H25_RS14490
B21	Aspartate 1-decarboxylase	SH83_RS02560
	Bis(5-nucleosyl)-tetraphosphatase (symmetrical) YqeK	SH83_RS02565
	AMP-binding protein	SH83_RS02570
	4-phosphopantetheinyl transferase superfamily protein	SH83_RS02575
	MFS transporter	SH83_RS02580
	Hypothetical protein	SH83_RS02590
	Hypothetical protein	SH83_RS03780
	DEAD/DEAH box helicase	SH83_RS03785
	Hypothetical protein	SH83_RS03790
	Helix-turn-helix domain-containing protein	SH83_RS03795
	LPXTG cell wall anchor domain-containing protein	SH83_RS03800
	Hypothetical protein	SH83_RS03805
	Hypothetical protein	SH83_RS03810
	Hypothetical protein	SH83_RS03815
	Protein rep	SH83_RS03830
	Hypothetical protein	SH83_RS03835
	Relaxase/mobilization nuclease domain-containing protein	SH83_RS03840
	Hypothetical protein	SH83_RS03845
	Hypothetical protein	SH83_RS03850
	DUF3173 domain-containing protein	SH83_RS03855
	Site-specific integrase	SH83_RS03860
	Cadmium resistance transporter	SH83_RS03865

	Winged helix-turn-helix transcriptional regulator	SH83_RS04765
	Hypothetical protein	SH83_RS04770
	Glycosyltransferase	SH83_RS05125
	CDP-glycerol glycerophosphotransferase family protein	SH83_RS05130
	Transposase	SH83_RS05140
	IS3 family transposase	SH83_RS05160
	Hypothetical protein	SH83_RS05165
	Hypothetical protein	SH83_RS08770
	Hypothetical protein	SH83_RS08920
	Hypothetical protein	SH83_RS08995
	Hypothetical protein	SH83_RS09005
	Hypothetical protein	SH83_RS09030
	Hypothetical protein	SH83_RS09035
	Hypothetical protein	SH83_RS10725
	Hypothetical protein	SH83_RS10730
	RusA family crossover junction endodeoxyribonuclease	SH83_RS10745
	Hypothetical protein	SH83_RS10770
	Hypothetical protein	SH83_RS13560
	NAD(P)-dependent alcohol dehydrogenase	SH83_RS13585
	Aldo/keto reductase	SH83_RS13595
	NAD(P)H-binding protein	SH83_RS13600
	DUF2255 family protein	SH83_RS13605
	SDR family oxidoreductase	SH83_RS13610
	LysR family transcriptional regulator	SH83_RS13615
	DUF927 domain-containing protein	SH83_RS13620
	Hypothetical protein	SH83_RS13640
	Hypothetical protein	SH83_RS13645
	Hypothetical protein	SH83_RS13650
	DUF853 family protein	SH83_RS13655
	Replication-relaxation family protein	SH83_RS15815
	Hypothetical protein	SH83_RS15820
	Helix-turn-helix domain-containing protein	SH83_RS15830
	LPXTG cell wall anchor domain-containing protein	SH83_RS15835
	Hypothetical protein	SH83_RS15840
	C40 family peptidase	SH83_RS15845
	Hypothetical protein	SH83_RS15850
	Hypothetical protein	SH83_RS15855
	ATP-binding protein	SH83_RS15860
	Hypothetical protein	SH83_RS15865
	Hypothetical protein	SH83_RS15870
	Conjugal transfer protein	SH83_RS15875
	Hypothetical protein	SH83_RS15880
	Class IIc cyclic bacteriocin	SH83_RS15885
	Stage II sporulation protein M	SH83_RS15890
	ABC transporter ATP-binding protein	SH83_RS15895
	ABC-2 transporter permease	SH83_RS15900
	Hypothetical protein	SH83_RS15905
	Hypothetical protein	SH83_RS15930
	Hypothetical protein	SH83_RS15935
	Hypothetical protein	SH83_RS15940
	Hypothetical protein	SH83_RS16030
	Hypothetical protein	SH83_RS16035
	DUF4145 domain-containing protein	SH83_RS16125
KACC 92189	Hypothetical protein	COO33_RS01695
	Zinc-ribbon domain-containing protein	COO33_RS01760
	Hypothetical protein	COO33_RS01775
	SocA family protein	COO33_RS01815
	LicD family protein	COO33_RS01820
	Restriction endonuclease subunit S	COO33_RS02570
	Helix-turn-helix transcriptional regulator	COO33_RS02715
	Hypothetical protein	COO33_RS02725
	Hypothetical protein	COO33_RS02730
	Hypothetical protein	COO33_RS02735

Hypothetical protein	COO33_RS02740
HlyD family secretion protein	COO33_RS02750
Peptide cleavage/export ABC transporter	COO33_RS05680
Helix-turn-helix transcriptional regulator	COO33_RS05685
Hypothetical protein	COO33_RS06895
Peptide cleavage/export ABC transporter	COO33_RS06900
AAA family ATPase	COO33_RS06920
AAA family ATPase	COO33_RS07320
Hypothetical protein	COO33_RS07325
Hypothetical protein	COO33_RS07330
RNA-directed DNA polymerase	COO33_RS07345
Phage head-tail connector protein	COO33_RS07350
Phage major capsid protein	COO33_RS07410
Phage portal protein	COO33_RS07420
Terminase large subunit	COO33_RS07425
Phage terminase small subunit P27 family	COO33_RS07435
Sporulation protein Cse60	COO33_RS07440
Virulence protein	COO33_RS07455
Hypothetical protein	COO33_RS07465
PTS transporter subunit EIIC	COO33_RS07475
Hypothetical protein	COO33_RS07930
HAD family hydrolase	COO33_RS07935
6-phospho-alpha-glucosidase	COO33_RS07940
PTS transporter subunit EIIC	COO33_RS07945
MurR/RpiR family transcriptional regulator	COO33_RS07950
Hypothetical protein	COO33_RS07955
AraC family transcriptional regulator	COO33_RS07960
Glycoside hydrolase family 38 C-terminal domain-containing protein	COO33_RS08395
LysR family transcriptional regulator	COO33_RS09500
FAD-dependent oxidoreductase	COO33_RS09865
GHKL domain-containing protein	COO33_RS09870
Response regulator transcription factor	COO33_RS10590
DUF2712 domain-containing protein	COO33_RS10595
Hypothetical protein	COO33_RS10600
Helix-turn-helix transcriptional regulator	COO33_RS11080
HNH endonuclease	COO33_RS12535
Hypothetical protein	COO33_RS12575
IS3 family transposase	COO33_RS12615
4-oxalocrotonate tautomerase	COO33_RS14145
C69 family dipeptidase	COO33_RS15620
Hypothetical protein	COO33_RS15625
CsbD family protein	COO33_RS15630
GlsB/YeaQ/YmgE family stress response membrane protein	COO33_RS15635
Hypothetical protein	COO33_RS15640
DeoR family transcriptional regulator	COO33_RS15650
DUF805 domain-containing protein	COO33_RS15655
Universal stress protein	COO33_RS15660
Tryptophan-rich sensory protein	COO33_RS15665
Hypothetical protein	COO33_RS15670
Antibiotic biosynthesis monooxygenase	COO33_RS15675
Lytic polysaccharide monooxygenase	COO33_RS15680
Hypothetical protein	COO33_RS15685
MFS transporter	COO33_RS15695
IS30 family transposase	COO33_RS15705
Hypothetical protein	COO33_RS15725
Hypothetical protein	COO33_RS15835
Hypothetical protein	COO33_RS15840
Hypothetical protein	COO33_RS15900
Polysaccharide pyruvyl transferase family protein	COO33_RS15930
MFS transporter	COO33_RS16005
IS30 family transposase	COO33_RS16010
Hypothetical protein	COO33_RS16020
LysM peptidoglycan-binding domain-containing protein	COO33_RS16025

KUP/HAK/KT family potassium transporter	COO33_RS16030
MarR family transcriptional regulator	COO33_RS16035
Recombinase family protein	COO33_RS16050
Hypothetical protein	COO33_RS16055
Putative holin-like toxin	COO33_RS16060
S41 family peptidase	COO33_RS16085
Hypothetical protein	COO33_RS16110
Type IA DNA topoisomerase	COO33_RS16125
Conjugal transfer protein	COO33_RS16130
Hypothetical protein	COO33_RS16135
Type IV secretory system conjugative DNA transfer family protein	COO33_RS16140
Hypothetical protein	COO33_RS16145
Hypothetical protein	COO33_RS16150
Hypothetical protein	COO33_RS16155
CHAP domain-containing protein	COO33_RS16160
Conjugal transfer protein	COO33_RS16165
CagC family type IV secretion system protein	COO33_RS16170
Hypothetical protein	COO33_RS16190
Hypothetical protein	COO33_RS16195
MobA/MobL family protein	COO33_RS16200
UV-resistance	COO33_RS16205
Hypothetical protein	COO33_RS16260
HXXEE domain-containing protein	COO33_RS16400
Hypothetical protein	COO33_RS16415
Putative holin-like toxin	COO33_RS16420
Y-family DNA polymerase	COO33_RS16425
Hypothetical protein	COO33_RS16430
Hypothetical protein	COO33_RS16435
Helix-turn-helix domain-containing protein	COO33_RS16440
S41 family peptidase	COO33_RS16445
Hypothetical protein	COO33_RS16450
Hypothetical protein	COO33_RS16460
Type IA DNA topoisomerase	COO33_RS16465
Conjugal transfer protein	COO33_RS16470
Hypothetical protein	COO33_RS16475
Type IV secretory system conjugative DNA transfer family protein	COO33_RS16480
Glycosyltransferase	COO33_RS16485
Hypothetical protein	COO33_RS16500
Glycosyltransferase	COO33_RS16505
Polysaccharide pyruvyl transferase family protein	COO33_RS16510
Glycosyltransferase family 2 protein	COO33_RS16515
Hypothetical protein	COO33_RS16525
MFS transporter	COO33_RS16560
MFS transporter	COO33_RS16565
Family 78 glycoside hydrolase catalytic domain	COO33_RS16570
Helix-turn-helix domain-containing protein	COO33_RS16575
Hypothetical protein	COO33_RS16580
Hypothetical protein	COO33_RS16635
Conjugal transfer protein	COO33_RS16650
DUF87 domain-containing protein	COO33_RS16655
Hypothetical protein	COO33_RS16660
Type IA DNA topoisomerase	COO33_RS16700
Putative holin-like toxin	COO33_RS16710
Sugar transferase	COO33_RS16725
Glycosyl transferase	COO33_RS16755
Multidrug MFS transporter	COO33_RS16765
Glycosyltransferase	COO33_RS16805
Hypothetical protein	COO33_RS16810
Hypothetical protein	COO33_RS16815
Phage major capsid protein	COO33_RS16825
Phage portal protein	COO33_RS16830
Terminase large subunit	COO33_RS16835
P27 family phage terminase small subunit	COO33_RS16840

	HNH endonuclease	COO33_RS16845
	Hypothetical protein	COO33_RS16850
	Hypothetical protein	COO33_RS16855
	Hypothetical protein	COO33_RS16860
	Phage gp6-like head-tail connector protein	COO33_RS16865
	Phage major capsid protein	COO33_RS16875
	Phage portal protein	COO33_RS16880
	Terminase large subunit	COO33_RS16885
	P27 family phage terminase small subunit	COO33_RS16890
	HNH endonuclease	COO33_RS16895
	Hypothetical protein	COO33_RS16900
	Hypothetical protein	COO33_RS16905
	Hypothetical protein	COO33_RS16910
	Hypothetical protein	COO33_RS16945
	Hypothetical protein	COO33_RS16960
	Hypothetical protein	COO33_RS16980
	Hypothetical protein	COO33_RS16985
	Hypothetical protein	COO33_RS16990
	Hypothetical protein	COO33_RS17030
	TetR/AcrR family transcriptional regulator	COO33_RS17045
	IS1380 family transposase	COO33_RS17050
KM2	MFS transporter	JQC82_RS04925
	MobA/MobL family protein	JQC82_RS15310
	DUF536 domain-containing protein	JQC82_RS15350
	Hypothetical protein	JQC82_RS15455
	Hypothetical protein	JQC82_RS15460
	Hypothetical protein	JQC82_RS15470
	MobA/MobL family protein	JQC82_RS15480
	Hypothetical protein	JQC82_RS15690
	ParA family protein	JQC82_RS15700
	Hypothetical protein	JQC82_RS15705
	Type II toxin-antitoxin system RelB/DinJ family antitoxin	JQC82_RS15710
	Hypothetical protein	JQC82_RS15720
	Magnesium transporter CorA family protein	JQC82_RS15725
	Hypothetical protein	JQC82_RS15740
	Hypothetical protein	JQC82_RS15750
	Class I SAM-dependent methyltransferase	JQC82_RS15755
	Putative holin-like toxin	JQC82_RS15955
	Recombinase family protein	JQC82_RS16030
	MFS transporter	JQC82_RS16095
	IS110 family transposase	JQC82_RS16100
MF1298	Hypothetical protein	ASV54_RS01195
	IS110 family transposase	ASV54_RS01775
	Hypothetical protein	ASV54_RS05460
	Hypothetical protein	ASV54_RS05510
	Hemolysin XhlA family protein	ASV54_RS05515
	IS110 family transposase	ASV54_RS05725
	IS110 family transposase	ASV54_RS06585
	IS110 family transposase	ASV54_RS07280
	IS110 family transposase	ASV54_RS07320
	Type 1 glutamine amidotransferase-like domain-containing protein	ASV54_RS08910
	IS110 family transposase	ASV54_RS09225
	IS110 family transposase	ASV54_RS11890
	Winged helix-turn-helix transcriptional regulator	ASV54_RS12625
	MFS transporter	ASV54_RS13155
	Hypothetical protein	ASV54_RS13160
	SIS domain-containing protein	ASV54_RS13405
	PTS sugar transporter subunit IIA	ASV54_RS13410
	PTS galactitol transporter subunit IIB	ASV54_RS13415
	PTS galactitol transporter subunit IIC	ASV54_RS13420
	RpiB/LacA/LacB family sugar-phosphate isomerase	ASV54_RS13425
	DeoR/GlpR transcriptional regulator	ASV54_RS13430
	Glucose 1-dehydrogenase	ASV54_RS13435

SIS domain-containing protein	ASV54_RS13440
Ribulose-phosphate 3-epimerase	ASV54_RS13445
PTS sugar transporter subunit IIB	ASV54_RS13450
PTS sugar transporter subunit IIA	ASV54_RS13460
FMN-binding protein	ASV54_RS13465
Hypothetical protein	ASV54_RS14815
Hypothetical protein	ASV54_RS14820
LysR family transcriptional regulator	ASV54_RS14880
OFA family MFS transporter	ASV54_RS15240
Acyl-CoA/acyl-ACP dehydrogenase	ASV54_RS15245
CoA transferase	ASV54_RS15250
Acyl-CoA dehydrogenase family protein	ASV54_RS15255
Hypothetical protein	ASV54_RS15260
Acyl--CoA ligase	ASV54_RS15265
IS30 family transposase	ASV54_RS15275
IS30 family transposase	ASV54_RS15290
Type 1 glutamine amidotransferase	ASV54_RS15605
Transcriptional regulator Spx	ASV54_RS15620
CadD family cadmium resistance transporter	ASV54_RS15625
Winged helix-turn-helix transcriptional regulator	ASV54_RS15630
FAD-dependent oxidoreductase	ASV54_RS15635
Hypothetical protein	ASV54_RS15640
Arsenite efflux transporter metallochaperone ArsD	ASV54_RS15650
Type I pullulanase	ASV54_RS15665
Type II toxin-antitoxin system RelB/DinJ family antitoxin	ASV54_RS15745
Hypothetical protein	ASV54_RS15760
UDP-galactopyranose mutase	ASV54_RS15770
Transposase	ASV54_RS15790
Transposase	ASV54_RS15835
Hsp20/alpha crystallin family protein	ASV54_RS15840
Hsp20/alpha crystallin family protein	ASV54_RS15845
PTS-dependent dihydroxyacetone kinase phosphotransferase subunit DhaM	ASV54_RS15850
Dihydroxyacetone kinase subunit L	ASV54_RS15870
Dihydroxyacetone kinase subunit DhaK	ASV54_RS15875
IS30 family transposase	ASV54_RS15880
Putative holin-like toxin	ASV54_RS15890
Winged helix-turn-helix transcriptional regulator	ASV54_RS16015
Arsenite efflux transporter metallochaperone ArsD	ASV54_RS16095
Arsenical pump-driving ATPase	ASV54_RS16100
Arsenic transporter	ASV54_RS16105
Hypothetical protein	ASV54_RS16110
Arsenic metallochaperone ArsD family protein	ASV54_RS16115
FAD-dependent oxidoreductase	ASV54_RS16120
Winged helix-turn-helix transcriptional regulator	ASV54_RS16125
CadD family cadmium resistance transporter	ASV54_RS16130
Transcriptional regulator Spx	ASV54_RS16135
Type 1 glutamine amidotransferase	ASV54_RS16140
Glycoside hydrolase family 68 protein	ASV54_RS16145
Nucleoside hydrolase	ASV54_RS16160
Pyruvate oxidase	ASV54_RS16180
Biotin transporter BioY	ASV54_RS16185
Hypothetical protein	ASV54_RS16190
Hypothetical protein	ASV54_RS16195
Type IA DNA topoisomerase	ASV54_RS16210
Metalloregulator ArsR/SmtB family transcription factor	ASV54_RS16255
Arsenic transporter	ASV54_RS16280
Sugar porter family MFS transporter	ASV54_RS16285
DUF4355 domain-containing protein	ASV54_RS16355
DUF305 domain-containing protein	ASV54_RS16370
Hypothetical protein	ASV54_RS16440
Copper-translocating P-type ATPase	ASV54_RS16460
CopY/TcrY family copper transport repressor	ASV54_RS16525
IS30 family transposase	ASV54_RS16530

IS30-like element ISLsa1 family transposase	ASV54_RS16555
DEAD/DEAH box helicase family protein	ASV54_RS16560
IS30 family transposase	ASV54_RS16565
IS3 family transposase	ASV54_RS16585
Helix-turn-helix domain-containing protein	ASV54_RS16620
IS30 family transposase	ASV54_RS16625
BRCT domain-containing protein	ASV54_RS16635
Hypothetical protein	ASV54_RS16660
Alpha-glucosidase	ASV54_RS16665
SLC45 family MFS transporter	ASV54_RS16670
Hypothetical protein	ASV54_RS16680
DNA starvation/stationary phase protection protein	ASV54_RS16715
AsnC family protein	ASV54_RS16755
Iron reductase	ASV54_RS16775
Hypothetical protein	ASV54_RS16785
Tyrosine-type recombinase/integrase	ASV54_RS16800
Helix-turn-helix transcriptional regulator	ASV54_RS16810
Hypothetical protein	ASV54_RS16820
IS30 family transposase	ASV54_RS16905
RNA-directed DNA polymerase	ASV54_RS16920
RNA-directed DNA polymerase	ASV54_RS16925
Ribonucleoside-triphosphate reductase, adenosylcobalamin-dependent	ASV54_RS16930
Hypothetical protein	ASV54_RS16950
Cob(I)yrinic acid a,c-diamide adenosyltransferase	ASV54_RS16955
FadR family transcriptional regulator	ASV54_RS16960
Histidine phosphatase family protein	ASV54_RS16965
Adenosylcobinamide-GDP ribazoletransferase	ASV54_RS16975
Hypothetical protein	ASV54_RS16980
ECF transporter S component	ASV54_RS16985
DDE-type integrase/transposase/recombinase	ASV54_RS16990
Energy-coupling factor transporter transmembrane protein EcfT	ASV54_RS16995
ATP-binding cassette domain-containing protein	ASV54_RS17000
ECF transporter S component	ASV54_RS17005
Fucose-binding lectin II	ASV54_RS17010
DUF4430 domain-containing protein	ASV54_RS17015
Hypothetical protein	ASV54_RS17020
IS5 family transposase	ASV54_RS17025
Oxidoreductase	ASV54_RS17075
FAD:protein FMN transferase	ASV54_RS17080
FMN-binding protein	ASV54_RS17085
Hypothetical protein	ASV54_RS17090
Glycosyltransferase family 2 protein	ASV54_RS17115
Response regulator transcription factor	ASV54_RS17120
Cadmium-translocating P-type ATPase	ASV54_RS17140
Tyrosine recombinase	ASV54_RS17150
IS30 family transposase	ASV54_RS17155
IS30 family transposase	ASV54_RS17160
Helix-turn-helix domain-containing protein	ASV54_RS17180
DUF2089 family protein	ASV54_RS17265
Hypothetical protein	ASV54_RS17280
ATP-binding protein	ASV54_RS17285
Hypothetical protein	ASV54_RS17325
Replication protein	ASV54_RS17360
Protein TrsL	ASV54_RS17365
Plasmid recombination protein	ASV54_RS17375
DNA starvation/stationary phase protection protein	ASV54_RS17380
Hypothetical protein	ASV54_RS17405
Hypothetical protein	ASV54_RS17430
Protein rep	ASV54_RS17445
Hypothetical protein	ASV54_RS17465
Ribbon-helix-helix protein, CopG family	ASV54_RS17470
GNAT family N-acetyltransferase	ASV54_RS17485
Hypothetical protein	ASV54_RS17495

	Hypothetical protein	ASV54_RS17505
	Hypothetical protein	ASV54_RS17545
	Hypothetical protein	ASV54_RS17560
	Electron transfer flavoprotein subunit alpha/FixB family protein	ASV54_RS17590
	Hypothetical protein	ASV54_RS17605
	Hypothetical protein	ASV54_RS17620
	RepB family plasmid replication initiator protein	ASV54_RS17655
	Hypothetical protein	ASV54_RS17680
	Hypothetical protein	ASV54_RS17690
	PTS sugar transporter subunit IIA	ASV54_RS17745
RI-113	PTS sugar transporter subunit IIB	BJD88_RS00005
	PTS ascorbate transporter subunit IIC	BJD88_RS00010
	Glutamate--cysteine ligase	BJD88_RS00015
	LTA synthase family protein	BJD88_RS03085
	YkuJ family protein	BJD88_RS07610
	Hypothetical protein	BJD88_RS07615
	Hypothetical protein	BJD88_RS10350
	Hypothetical protein	BJD88_RS10355
	Abi family protein	BJD88_RS10360
	Hypothetical protein	BJD88_RS10365
	ImmA/IrrE family metallo-endopeptidase	BJD88_RS10640
	Hypothetical protein	BJD88_RS11090
	IS256 family transposase	BJD88_RS11095
	Thioredoxin family protein	BJD88_RS13890
	IS5 family transposase	BJD88_RS14655
	PTS sugar transporter subunit IIB	BJD88_RS14810
	Beta-galactosidase	BJD88_RS15910
	Beta-galactosidase small subunit	BJD88_RS15955
	Hypothetical protein	BJD88_RS15960
	IS3 family transposase	BJD88_RS16010
	Cof-type HAD-IIB family hydrolase	BJD88_RS16070
	Beta-phosphoglucomutase	BJD88_RS16075
	Glycoside hydrolase family 65 protein	BJD88_RS16080
	Hypothetical protein	BJD88_RS16085
	ABC transporter permease	BJD88_RS16095
	IS5 family transposase	BJD88_RS16120
	ABC transporter ATP-binding protein	BJD88_RS16140
	Iron chelate uptake ABC transporter family permease subunit	BJD88_RS16150
	ABC transporter substrate-binding protein	BJD88_RS16155
	NEAT domain-containing protein	BJD88_RS16160
	Hypothetical protein	BJD88_RS16165
	Cytochrome P450	BJD88_RS16170
	IS5 family transposase	BJD88_RS16175
	IS6 family transposase	BJD88_RS16200
	MFS transporter	BJD88_RS16230
	Carboxylating nicotinate-nucleotide diphosphorylase	BJD88_RS16245
	Aminotransferase class V-fold PLP-dependent enzyme	BJD88_RS16250
	Quinolinate synthase NadA	BJD88_RS16270
	FAD-binding protein	BJD88_RS16275
	Amino acid permease	BJD88_RS16280
	Transposase	BJD88_RS16300
	PTS sugar transporter subunit IIA	BJD88_RS16315
	NAD(P)H-binding protein	BJD88_RS16320
	Ldh family oxidoreductase	BJD88_RS16340
	IS5 family transposase	BJD88_RS16345
	IS5 family transposase	BJD88_RS16450
	Amino acid ABC transporter permease	BJD88_RS16465
	Amino acid ABC transporter permease	BJD88_RS16475
	Amino acid ABC transporter ATP-binding protein	BJD88_RS16480
	Transporter substrate-binding domain-containing protein	BJD88_RS16485
	Hypothetical protein	BJD88_RS16490
	PTS sugar transporter subunit IIA	BJD88_RS16510
	Transketolase	BJD88_RS16515

	Transposase	BJD88_RS16530
	LysR family transcriptional regulator	BJD88_RS16545
	FAD-binding protein	BJD88_RS16550
	Helix-turn-helix domain-containing protein	BJD88_RS16555
	PTS transporter subunit EIIC	BJD88_RS16570
	ChbG/HpnK family deacetylase	BJD88_RS16575
	Glycoside hydrolase family 1 protein	BJD88_RS16590
	Glycoside hydrolase family 1 protein	BJD88_RS16595
	Hypothetical protein	BJD88_RS16600
	IS30 family transposase	BJD88_RS16605
	Hypothetical protein	BJD88_RS16630
	Type IA DNA topoisomerase	BJD88_RS16670
	Hypothetical protein	BJD88_RS16685
	Restriction endonuclease subunit S	BJD88_RS16825
	N-6 DNA methylase	BJD88_RS16835
	Hypothetical protein	BJD88_RS16840
	Type II toxin-antitoxin system RelB/DinJ family antitoxin	BJD88_RS16850
	SDR family oxidoreductase	BJD88_RS16880
	Phosphate/phosphite/phosphonate ABC transporter substrate-binding protein	BJD88_RS16940
	Phosphonate ABC transporter ATP-binding protein	BJD88_RS16945
	Phosphonate ABC transporter, permease protein PhnE	BJD88_RS16950
	Phosphonate ABC transporter, permease protein PhnE	BJD88_RS16955
	Bifunctional metallophosphatase/5-nucleotidase	BJD88_RS16960
	Hypothetical protein	BJD88_RS16965
	DNA starvation/stationary phase protection protein	BJD88_RS16970
	Recombinase family protein	BJD88_RS16990
	Metal-sensitive transcriptional regulator	BJD88_RS17015
	PspC domain-containing protein	BJD88_RS17035
	IS30-like element ISLpl1 family transposase	BJD88_RS17065
	Hypothetical protein	BJD88_RS17075
	ABC transporter ATP-binding protein	BJD88_RS17135
	IS30 family transposase	BJD88_RS17160
	IS5 family transposase	BJD88_RS17175
	IS5-like element ISLpl3 family transposase	BJD88_RS17215
	IS5-like element ISLpl3 family transposase	BJD88_RS17245
	IS5-like element ISLpl3 family transposase	BJD88_RS17300
	XkdX family protein	BJD88_RS17335
	Hypothetical protein	BJD88_RS17410
	IS5 family transposase	BJD88_RS17420
	DUF536 domain-containing protein	BJD88_RS17490
	PTS sugar transporter subunit IIC	BJD88_RS17520
	Hypothetical protein	BJD88_RS17580
	Hypothetical protein	BJD88_RS17635
	ATPase	BJD88_RS17720
	Hypothetical protein	BJD88_RS17725
BLS41	Hypothetical protein	BOQ65_RS00005
	Phage tail protein	BOQ65_RS00010
	Hypothetical protein	BOQ65_RS00020
	Hypothetical protein	BOQ65_RS00025
	Phage tail tape measure protein	BOQ65_RS00030
	Phage tail family protein	BOQ65_RS00035
	DUF1617 family protein	BOQ65_RS00040
	Hypothetical protein	BOQ65_RS00050
	Hypothetical protein	BOQ65_RS00055
	Hypothetical protein	BOQ65_RS00060
	Lysis protein	BOQ65_RS00065
	SH3 domain-containing protein	BOQ65_RS00070
	Tyrosine-type recombinase/integrase	BOQ65_RS00075
	DUF4062 domain-containing protein	BOQ65_RS15515
	Hypothetical protein	BOQ65_RS15520
	Hypothetical protein	BOQ65_RS15525
	Hypothetical protein	BOQ65_RS15530
	Helix-turn-helix transcriptional regulator	BOQ65_RS15535

	Hypothetical protein	BOQ65_RS15540
	Hypothetical protein	BOQ65_RS15545
	DUF771 domain-containing protein	BOQ65_RS15550
	Hypothetical protein	BOQ65_RS15555
	Hypothetical protein	BOQ65_RS15560
	Siphovirus Gp157 family protein	BOQ65_RS15565
	DEAD/DEAH box helicase family protein	BOQ65_RS15570
	Helix-turn-helix transcriptional regulator	BOQ65_RS15575
	AAA family ATPase	BOQ65_RS15580
	Hypothetical protein	BOQ65_RS15585
	Helicase	BOQ65_RS15590
	VRR-NUC domain-containing protein	BOQ65_RS15600
	Hypothetical protein	BOQ65_RS15605
	DUF1642 domain-containing protein	BOQ65_RS15615
	Hypothetical protein	BOQ65_RS15620
	Hypothetical protein	BOQ65_RS15625
	DUF3775 domain-containing protein	BOQ65_RS15630
	Clp protease ClpP	BOQ65_RS15645
	Hypothetical protein	BOQ65_RS15685
	Hypothetical protein	BOQ65_RS15700
	Hypothetical protein	BOQ65_RS15705
	Phage tail protein	BOQ65_RS15710
	Hypothetical protein	BOQ65_RS15715
	Hypothetical protein	BOQ65_RS15720
	Phage tail tape measure protein	BOQ65_RS15725
	Phage tail family protein	BOQ65_RS15730
	DUF1617 family protein	BOQ65_RS15735
	Hypothetical protein	BOQ65_RS15745
	Hypothetical protein	BOQ65_RS15750
	Class I SAM-dependent methyltransferase	BOQ65_RS15805
	Hypothetical protein	BOQ65_RS16055
	Transposase	BOQ65_RS16060
	Hypothetical protein	BOQ65_RS16635
	Hypothetical protein	BOQ65_RS16640
	MFS transporter	BOQ65_RS16645
	Recombinase family protein	BOQ65_RS16695
	IS1380 family transposase	BOQ65_RS16700
	TetR/AcrR family transcriptional regulator	BOQ65_RS16725
	MFS transporter	BOQ65_RS16840
	Recombinase family protein	BOQ65_RS16845
	Rgg/GadR/MutR family transcriptional regulator	BOQ65_RS16850
	ThiF family adenylyltransferase	BOQ65_RS16860
	MFS transporter	BOQ65_RS16865
	Hypothetical protein	BOQ65_RS16870
	YxeA family protein	BOQ65_RS16965
	DUF536 domain-containing protein	BOQ65_RS16970
	Hypothetical protein	BOQ65_RS16975
	Hypothetical protein	BOQ65_RS16980
	ATP-binding cassette domain-containing protein	BOQ65_RS16985
	Hypothetical protein	BOQ65_RS16995
	Hypothetical protein	BOQ65_RS17000
	Putative holin-like toxin	BOQ65_RS17125
	Hypothetical protein	BOQ65_RS17175
	IS110 family transposase	BOQ65_RS17290
DSR_M2	Hypothetical protein	CFN49_RS00020
	Hypothetical protein	CFN49_RS04340
	MFS transporter	CFN49_RS05165
	Hypothetical protein	CFN49_RS07960
	Hypothetical protein	CFN49_RS08970
	Hypothetical protein	CFN49_RS08975
	Hypothetical protein	CFN49_RS10005
	Tyrosine-type recombinase/integrase	CFN49_RS10010
	Hypothetical protein	CFN49_RS10015

	Hypothetical protein	CFN49_RS10020
	Hypothetical protein	CFN49_RS10025
	Hypothetical protein	CFN49_RS10030
	Hypothetical protein	CFN49_RS10035
	Hypothetical protein	CFN49_RS10040
	Hypothetical protein	CFN49_RS10045
	Hypothetical protein	CFN49_RS10050
	Hypothetical protein	CFN49_RS10060
	Hypothetical protein	CFN49_RS10065
	Hypothetical protein	CFN49_RS10070
	Hypothetical protein	CFN49_RS10075
	Abi family protein	CFN49_RS10080
	LysM peptidoglycan-binding domain-containing protein	CFN49_RS10085
	Hypothetical protein	CFN49_RS10090
	Recombinase family protein	CFN49_RS10095
	Site-specific DNA-methyltransferase	CFN49_RS10100
	Hypothetical protein	CFN49_RS10110
	Hypothetical protein	CFN49_RS10115
	Hypothetical protein	CFN49_RS10125
	Site-specific DNA-methyltransferase	CFN49_RS10130
	Rgg/GadR/MutR family transcriptional regulator	CFN49_RS10135
	DNA starvation/stationary phase protection protein	CFN49_RS12770
	IS256 family transposase	CFN49_RS13795
	Hypothetical protein	CFN49_RS15580
IDCC3501	Hypothetical protein	D0Y51_RS04945
	Hypothetical protein	D0Y51_RS05055
	Hypothetical protein	D0Y51_RS05075
	Hypothetical protein	D0Y51_RS05080
	PTS sugar transporter subunit IIA	D0Y51_RS05095
	Hypothetical protein	D0Y51_RS07240
	Hypothetical protein	D0Y51_RS11525
	Glycosyltransferase	D0Y51_RS14915
	Hypothetical protein	D0Y51_RS15275
	Hypothetical protein	D0Y51_RS15290
	Hypothetical protein	D0Y51_RS15295
	Acyltransferase	D0Y51_RS15300
	Hypothetical protein	D0Y51_RS15310
	Transposase	D0Y51_RS15315
	Sugar transferase	D0Y51_RS15330
	Glycosyl transferase	D0Y51_RS15365
	LicD family protein	D0Y51_RS15400
	Glycosyltransferase	D0Y51_RS15410
	Hypothetical protein	D0Y51_RS15415
	Glycosyltransferase family 2 protein	D0Y51_RS15430
	Hypothetical protein	D0Y51_RS15435
KC28	Hypothetical protein	C4O30_RS00005
	Phage tail family protein	C4O30_RS00010
	Phage tail protein	C4O30_RS00015
	Hypothetical protein	C4O30_RS00020
	Hypothetical protein	C4O30_RS00025
	Hypothetical protein	C4O30_RS00030
	Hypothetical protein	C4O30_RS00035
	Hypothetical protein	C4O30_RS00040
	DUF4393 domain-containing protein	C4O30_RS00050
	Hypothetical protein	C4O30_RS00095
	Helix-turn-helix transcriptional regulator	C4O30_RS00125
	Helix-turn-helix transcriptional regulator	C4O30_RS00135
	Hypothetical protein	C4O30_RS00140
	DUF2513 domain-containing protein	C4O30_RS00150
	Helix-turn-helix transcriptional regulator	C4O30_RS00155
	Helix-turn-helix transcriptional regulator	C4O30_RS00160
	Hypothetical protein	C4O30_RS00165
	PBSX family phage terminase large subunit	C4O30_RS00255

Phage portal protein	C4O30_RS00280
Minor capsid protein	C4O30_RS00285
DUF4355 domain-containing protein	C4O30_RS00290
Hypothetical protein	C4O30_RS00295
Major capsid protein	C4O30_RS00300
Phage head-tail connector protein	C4O30_RS00305
Hypothetical protein	C4O30_RS00310
Hypothetical protein	C4O30_RS00315
Hypothetical protein	C4O30_RS00320
Hypothetical protein	C4O30_RS00325
Hypothetical protein	C4O30_RS00330
Hypothetical protein	C4O30_RS00335
Tape measure protein	C4O30_RS00340
Hypothetical protein	C4O30_RS00345
Phage tail family protein	C4O30_RS00350
Phage tail protein	C4O30_RS00355
Hypothetical protein	C4O30_RS00360
Hypothetical protein	C4O30_RS00365
Phage baseplate upper protein	C4O30_RS00370
Hypothetical protein	C4O30_RS00375
Hypothetical protein	C4O30_RS00380
XkdX family protein	C4O30_RS00385
LysM peptidoglycan-binding domain-containing protein	C4O30_RS00390
Holin	C4O30_RS00395
Hypothetical protein	C4O30_RS00405
Hypothetical protein	C4O30_RS00410
Helix-turn-helix transcriptional regulator	C4O30_RS04615
Helix-turn-helix transcriptional regulator	C4O30_RS04620
Helix-turn-helix transcriptional regulator	C4O30_RS04625
Hypothetical protein	C4O30_RS04630
Hypothetical protein	C4O30_RS04635
Hypothetical protein	C4O30_RS04640
Hypothetical protein	C4O30_RS04660
Terminase small subunit	C4O30_RS04700
Hypothetical protein	C4O30_RS04705
Holin	C4O30_RS05475
Phage major capsid protein	C4O30_RS05480
DUF1056 family protein	C4O30_RS05560
Hypothetical protein	C4O30_RS05570
Hypothetical protein	C4O30_RS05595
Hypothetical protein	C4O30_RS05605
HNH endonuclease	C4O30_RS05610
Hypothetical protein	C4O30_RS05615
Site-specific integrase	C4O30_RS05620
Hypothetical protein	C4O30_RS05635
CsbD family protein	C4O30_RS08155
Hypothetical protein	C4O30_RS08275
DUF4373 domain-containing protein	C4O30_RS08295
ImmA/IrrE family metallo-endopeptidase	C4O30_RS08330
Hypothetical protein	C4O30_RS08395
FRG domain-containing protein	C4O30_RS08420
ImmA/IrrE family metallo-endopeptidase	C4O30_RS13215
Tyrosine-type recombinase/integrase	C4O30_RS13220
DUF4393 domain-containing protein	C4O30_RS15975
Hypothetical protein	C4O30_RS15980
Hypothetical protein	C4O30_RS16000
Hypothetical protein	C4O30_RS16005
ImmA/IrrE family metallo-endopeptidase	C4O30_RS16010
Helix-turn-helix transcriptional regulator	C4O30_RS16015
Helix-turn-helix transcriptional regulator	C4O30_RS16020
Hypothetical protein	C4O30_RS16025
Hypothetical protein	C4O30_RS16030
DUF2513 domain-containing protein	C4O30_RS16035

	Helix-turn-helix transcriptional regulator	C4O30_RS16040
	Helix-turn-helix transcriptional regulator	C4O30_RS16045
	Hypothetical protein	C4O30_RS16050
	Hypothetical protein	C4O30_RS16055
	Hypothetical protein	C4O30_RS16060
	DNA-binding protein	C4O30_RS16065
	Hypothetical protein	C4O30_RS16070
	Hypothetical protein	C4O30_RS16075
	Recombinase RecT	C4O30_RS16080
	PD-(D/E)XK nuclease-like domain-containing protein	C4O30_RS16085
	DnaD domain protein	C4O30_RS16090
	Hypothetical protein	C4O30_RS16095
	Hypothetical protein	C4O30_RS16100
	Hypothetical protein	C4O30_RS16105
	Hypothetical protein	C4O30_RS16110
	DUF1642 domain-containing protein	C4O30_RS16115
	Hypothetical protein	C4O30_RS16120
	Hypothetical protein	C4O30_RS16140
	Terminase small subunit	C4O30_RS16155
	Hypothetical protein	C4O30_RS16160
	Hypothetical protein	C4O30_RS16165
	Hypothetical protein	C4O30_RS16170
	Hypothetical protein	C4O30_RS16210
	Hypothetical protein	C4O30_RS16215
	ATP-binding protein	C4O30_RS16305
LMT1-48	Hypothetical protein	EGU28_RS00010
	Clp protease ClpP	EGU28_RS00030
	Phage major capsid protein	EGU28_RS00085
	Phage tail spike protein	EGU28_RS00090
	Hypothetical protein	EGU28_RS00140
	Hypothetical protein	EGU28_RS00145
	Hemolysin XhlA family protein	EGU28_RS00150
	Holin	EGU28_RS00165
	2-hydroxymuconic semialdehyde hydrolase	EGU28_RS00170
	Hypothetical protein	EGU28_RS00180
	Hypothetical protein	EGU28_RS00875
	Hypothetical protein	EGU28_RS00940
	Hypothetical protein	EGU28_RS00945
	Hypothetical protein	EGU28_RS00980
	HNH endonuclease	EGU28_RS00985
	Phage terminase small subunit P27 family	EGU28_RS01010
	DUF2726 domain-containing protein	EGU28_RS01015
	DUF1056 family protein	EGU28_RS01020
	Phage portal protein	EGU28_RS01030
	Phage gp6-like head-tail connector protein	EGU28_RS01035
	Hypothetical protein	EGU28_RS01050
	Phage tail family protein	EGU28_RS01080
	DUF1617 family protein	EGU28_RS01090
	Hypothetical protein	EGU28_RS05620
	Hypothetical protein	EGU28_RS05735
	Helix-turn-helix transcriptional regulator	EGU28_RS05755
	IS30-like element ISLsa1 family transposase	EGU28_RS06015
	IS30 family transposase	EGU28_RS06105
	IS30-like element ISLsa1 family transposase	EGU28_RS06615
	Hypothetical protein	EGU28_RS07070
	IS256-like element IS1310 family transposase	EGU28_RS09790
	IS30-like element ISLsa1 family transposase	EGU28_RS10800
	IS30 family transposase	EGU28_RS10825
	Alpha-xylosidase	EGU28_RS10975
	Extracellular solute-binding protein	EGU28_RS11270
	Sugar ABC transporter permease	EGU28_RS11295
	Carbohydrate ABC transporter permease	EGU28_RS11300
	Hypothetical protein	EGU28_RS11305

	IS30-like element ISLsa1 family transposase	EGU28_RS11310
	Helix-turn-helix domain-containing protein	EGU28_RS11590
	Hypothetical protein	EGU28_RS12945
	Hypothetical protein	EGU28_RS13050
	ImmA/IrrE family metallo-endopeptidase	EGU28_RS13055
	Helix-turn-helix transcriptional regulator	EGU28_RS15440
	Putative HNHc nuclease	EGU28_RS15450
	D-2-hydroxyacid dehydrogenase	EGU28_RS15490
	IS30 family transposase	EGU28_RS15545
	Hypothetical protein	EGU28_RS15575
	Hypothetical protein	EGU28_RS15585
	IS30 family transposase	EGU28_RS15595
	Universal stress protein	EGU28_RS15640
	Amino acid permease	EGU28_RS15645
	IS3 family transposase	EGU28_RS15650
	IS30 family transposase	EGU28_RS15660
	Aldehyde dehydrogenase family protein	EGU28_RS15705
	Sugar transferase	EGU28_RS15725
	Transcriptional regulator Spx	EGU28_RS15775
	Hypothetical protein	EGU28_RS15815
	GntR family transcriptional regulator	EGU28_RS15850
	Deoxyribose-phosphate aldolase	EGU28_RS15855
	PTS sugar transporter subunit IIA	EGU28_RS15860
	DUF5320 domain-containing protein	EGU28_RS15865
	Type IV secretory system conjugative DNA transfer family protein	EGU28_RS15895
	Hypothetical protein	EGU28_RS16035
	Hypothetical protein	EGU28_RS16065
	AlwI family type II restriction endonuclease	EGU28_RS16070
	Hypothetical protein	EGU28_RS16080
	Hypothetical protein	EGU28_RS16180
	Hypothetical protein	EGU28_RS16200
	Hypothetical protein	EGU28_RS16210
	Hypothetical protein	EGU28_RS16230
	Hypothetical protein	EGU28_RS16345
	Hypothetical protein	EGU28_RS16360
	Helix-turn-helix domain-containing protein	EGU28_RS16370
83-18	Hypothetical protein	GPK32_RS00005
	Hypothetical protein	GPK32_RS00010
	Nucleotidyltransferase	GPK32_RS00015
	Hypothetical protein	GPK32_RS00020
	Hypothetical protein	GPK32_RS00120
	Amidohydrolase family protein	GPK32_RS00145
	Oligosaccharide flippase family protein	GPK32_RS00160
	Asparagine synthase	GPK32_RS00165
	CDP-glycerol glycerophosphotransferase family protein	GPK32_RS00170
	EpsG family protein	GPK32_RS00180
	Glycosyltransferase	GPK32_RS00190
	Sugar transferase	GPK32_RS00200
	Polysaccharide biosynthesis tyrosine autokinase	GPK32_RS00205
	Chain-length determining protein	GPK32_RS00215
	IS30 family transposase	GPK32_RS00220
	IS256 family transposase	GPK32_RS00225
	IS30-like element ISLpl1 family transposase	GPK32_RS00245
	Group II intron reverse transcriptase/maturase	GPK32_RS00310
	Glycosyltransferase	GPK32_RS00355
	Acyltransferase	GPK32_RS00395
	Glycosyltransferase	GPK32_RS00400
	Hypothetical protein	GPK32_RS00405
	Hypothetical protein	GPK32_RS00415
	IS3 family transposase	GPK32_RS00420
	IS3 family transposase	GPK32_RS00440
	Glycosyltransferase	GPK32_RS00445
	IS30-like element ISLsa1 family transposase	GPK32_RS00470

DNA topoisomerase III	GPK32_RS00485
Hypothetical protein	GPK32_RS00535
Hypothetical protein	GPK32_RS00675
Type IV secretory system conjugative DNA transfer family protein	GPK32_RS00680
Hypothetical protein	GPK32_RS00685
Hypothetical protein	GPK32_RS00690
DUF975 family protein	GPK32_RS00695
Glycosyltransferase	GPK32_RS00700
Hypothetical protein	GPK32_RS00730
Hypothetical protein	GPK32_RS00745
Hypothetical protein	GPK32_RS00755
Replication protein	GPK32_RS00760
IS5-like element ISLpl3 family transposase	GPK32_RS00785
IS30 family transposase	GPK32_RS00870
Cadmium-translocating P-type ATPase	GPK32_RS00950
Arsenate reductase (thioredoxin)	GPK32_RS00955
Recombinase family protein	GPK32_RS00965
Tn3 family transposase	GPK32_RS00990
IS30-like element ISLpl1 family transposase	GPK32_RS00995
IS5-like element ISLpl3 family transposase	GPK32_RS01030
Replication protein	GPK32_RS01075
Type II toxin-antitoxin system RelB/DinJ family antitoxin	GPK32_RS01115
IS256 family transposase	GPK32_RS01120
Helix-turn-helix domain-containing protein	GPK32_RS01155
Beta-galactosidase	GPK32_RS01160
Beta-galactosidase	GPK32_RS01205
IS5-like element ISLpl3 family transposase	GPK32_RS01210
DegV family protein	GPK32_RS01250
DUF1836 domain-containing protein	GPK32_RS01325
IS30-like element ISLpl1 family transposase	GPK32_RS01330
Hypothetical protein	GPK32_RS01335
Hypothetical protein	GPK32_RS01375
Hypothetical protein	GPK32_RS01380
AAA family ATPase	GPK32_RS01395
Type II toxin-antitoxin system PemK/MazF family toxin	GPK32_RS01400
Hypothetical protein	GPK32_RS01405
Hypothetical protein	GPK32_RS01445
Hypothetical protein	GPK32_RS01470
IS5-like element ISLpl3 family transposase	GPK32_RS01475
Hypothetical protein	GPK32_RS03185
Host-nuclease inhibitor Gam family protein	GPK32_RS03955
Hypothetical protein	GPK32_RS04025
Hypothetical protein	GPK32_RS04030
BppU family phage baseplate upper protein	GPK32_RS04160
XkdX family protein	GPK32_RS04165
Holin	GPK32_RS04175
Hypothetical protein	GPK32_RS04190
Hypothetical protein	GPK32_RS04195
Hypothetical protein	GPK32_RS04200
IS5-like element ISLpl3 family transposase	GPK32_RS04205
KxYKxGKxW signal peptide domain-containing protein	GPK32_RS04570
IS256 family transposase	GPK32_RS04715
IS3 family transposase	GPK32_RS06480
IS3 family transposase	GPK32_RS09800
Hypothetical protein	GPK32_RS09805
Type I restriction enzyme HsdR N-terminal domain-containing protein	GPK32_RS11505
IS5 family transposase	GPK32_RS11510
Putative DNA binding domain-containing protein	GPK32_RS11525
Hypothetical protein	GPK32_RS11555
Helix-turn-helix transcriptional regulator	GPK32_RS11640
Hypothetical protein	GPK32_RS11665
BppU family phage baseplate upper protein	GPK32_RS11720
Hypothetical protein	GPK32_RS11735

	Hypothetical protein	GPK32_RS11740
	Hypothetical protein	GPK32_RS11855
	Hypothetical protein	GPK32_RS11860
	Hypothetical protein	GPK32_RS11865
	DUF4373 domain-containing protein	GPK32_RS11870
	Hypothetical protein	GPK32_RS11880
	Hypothetical protein	GPK32_RS11940
	IS5-like element ISLpl3 family transposase	GPK32_RS11950
	IS5 family transposase	GPK32_RS12070
	IS30 family transposase	GPK32_RS14285
	Beta-phosphoglucomutase	GPK32_RS14335
	Aldose 1-epimerase	GPK32_RS14345
	Glycoside hydrolase family 65 protein	GPK32_RS14350
	MFS transporter	GPK32_RS14355
	LacI family DNA-binding transcriptional regulator	GPK32_RS14360
	Hypothetical protein	GPK32_RS14365
	Hypothetical protein	GPK32_RS14380
	Hypothetical protein	GPK32_RS14415
	Hypothetical protein	GPK32_RS14420
	Hypothetical protein	GPK32_RS14425
	Alpha/beta hydrolase	GPK32_RS14430
	Alpha/beta hydrolase	GPK32_RS14435
	DUF2357 domain-containing protein	GPK32_RS14440
	AAA family ATPase	GPK32_RS14450
	Hypothetical protein	GPK32_RS14455
	PrsW family intramembrane metalloprotease	GPK32_RS14460
	Hypothetical protein	GPK32_RS14465
	IS256 family transposase	GPK32_RS14470
	Amino acid permease	GPK32_RS14505
	KUP/HAK/KT family potassium transporter	GPK32_RS14895
	Hypothetical protein	GPK32_RS14900
	Helix-turn-helix transcriptional regulator	GPK32_RS15300
	IS5-like element ISLpl3 family transposase	GPK32_RS15340
	IS30-like element ISLpl1 family transposase	GPK32_RS16135
	Hypothetical protein	GPK32_RS16380
	Hypothetical protein	GPK32_RS16410
	Hypothetical protein	GPK32_RS16445
	ISL3-like element ISP1 family transposase	GPK32_RS16490
BF_15	ISL3-like element ISP1 family transposase	LTG66_RS09395
	ISL3-like element ISP1 family transposase	LTG66_RS09910
	Aldehyde dehydrogenase	LTG66_RS10795
	LysR substrate-binding domain-containing protein	LTG66_RS00190
	Zinc-binding dehydrogenase	LTG66_RS00240
	Hypothetical protein	LTG66_RS00795
	Hypothetical protein	LTG66_RS01245
	Hypothetical protein	LTG66_RS01270
	KilA-N domain-containing protein	LTG66_RS03325
	Hypothetical protein	LTG66_RS03650
	Hypothetical protein	LTG66_RS03830
	Hypothetical protein	LTG66_RS04940
	Hypothetical protein	LTG66_RS04945
	Extracellular solute-binding protein	LTG66_RS04950
	Hypothetical protein	LTG66_RS05535
	Hypothetical protein	LTG66_RS08450
	Hypothetical protein	LTG66_RS10250
	AIR synthase related protein	LTG66_RS10625
	SRPBCC family protein	LTG66_RS11540
	IS30 family transposase	LTG66_RS12895
	KxYKxGKxW signal peptide domain-containing protein	LTG66_RS13095
	YfhO family protein	LTG66_RS13200
	NAD(P)H-dependent oxidoreductase	LTG66_RS13545
	IS30 family transposase	LTG66_RS13720
	Hypothetical protein	LTG66_RS14795

	IS30 family transposase	LTG66_RS15505
	PKD domain-containing protein	LTG66_RS15565
FLPL05	Hypothetical protein	GJS00_RS00760
	Hypothetical protein	GJS00_RS00770
	DUF4422 domain-containing protein	GJS00_RS03280
	Hypothetical protein	GJS00_RS04800
	Hypothetical protein	GJS00_RS04810
	Hypothetical protein	GJS00_RS04915
	AAA family ATPase	GJS00_RS04975
	DUF669 domain-containing protein	GJS00_RS07180
	Hypothetical protein	GJS00_RS07185
	Transcriptional regulator	GJS00_RS07200
	Hypothetical protein	GJS00_RS07210
	Hypothetical protein	GJS00_RS07220
	Hypothetical protein	GJS00_RS07535
	ISL3-like element ISP1 family transposase	GJS00_RS08745
	Hypothetical protein	GJS00_RS08840
	Hypothetical protein	GJS00_RS09055
	Hypothetical protein	GJS00_RS09065
	Hypothetical protein	GJS00_RS09070
	Hypothetical protein	GJS00_RS09075
	Hypothetical protein	GJS00_RS09080
	Hypothetical protein	GJS00_RS09145
	Hypothetical protein	GJS00_RS09150
	IS3 family transposase	GJS00_RS09160
	TetR/AcrR family transcriptional regulator	GJS00_RS09200
	Hypothetical protein	GJS00_RS12295
	Hypothetical protein	GJS00_RS13370
	Mor transcription activator family protein	GJS00_RS13380
	Mor transcription activator family protein	GJS00_RS13405
	Site-specific integrase	GJS00_RS13415
	Phage/plasmid primase, P4 family	GJS00_RS13435
	Hypothetical protein	GJS00_RS13445
	DNA polymerase	GJS00_RS13450
	Hypothetical protein	GJS00_RS13455
	Hypothetical protein	GJS00_RS13460
	TetR/AcrR family transcriptional regulator	GJS00_RS13465
	Hypothetical protein	GJS00_RS14585
LPT52	MFS transporter	L0056_RS00870
	Hypothetical protein	L0056_RS01655
	DUF1617 family protein	L0056_RS02495
	CDP-glycerol glycerophosphotransferase family protein	L0056_RS02640
	Polysaccharide pyruvyl transferase family protein	L0056_RS05065
	Acyltransferase family protein	L0056_RS05085
	Polysaccharide deacetylase family protein	L0056_RS05095
	Peptidoglycan amidohydrolase family protein	L0056_RS05100
	Glycerol-3-phosphate cytidylyltransferase	L0056_RS05105
	EpsG family protein	L0056_RS05150
	CDP-glycerol glycerophosphotransferase family protein	L0056_RS05155
	Glycosyltransferase family 2 protein	L0056_RS05160
	Acyltransferase family protein	L0056_RS05170
	ISL3-like element ISP1 family transposase	L0056_RS05180
	Hypothetical protein	L0056_RS09160
	Hypothetical protein	L0056_RS10425
	Hypothetical protein	L0056_RS10525
	Hypothetical protein	L0056_RS10530
	Hypothetical protein	L0056_RS10565
	Hypothetical protein	L0056_RS10570
	Hypothetical protein	L0056_RS10575
	Hypothetical protein	L0056_RS10580
	Hypothetical protein	L0056_RS10655
	MMPL family transporter	L0056_RS10835
	NAD(P)H-binding protein	L0056_RS10980

	Hypothetical protein	L0056_RS13060
	Hypothetical protein	L0056_RS13115
LZ95	Recombinase RecT	AD081_RS05505
	PD-(D/E)XK nuclease-like domain-containing protein	AD081_RS05510
	DnaD domain protein	AD081_RS05515
	Hypothetical protein	AD081_RS05520
	Hypothetical protein	AD081_RS05535
	Hypothetical protein	AD081_RS05575
	Phage portal protein	AD081_RS05580
	Minor capsid protein	AD081_RS05600
	Hypothetical protein	AD081_RS05605
	DUF4355 domain-containing protein	AD081_RS05610
	Hypothetical protein	AD081_RS05615
	Phage head-tail connector protein	AD081_RS05620
	Hypothetical protein	AD081_RS05625
	HK97 gp10 family phage protein	AD081_RS05630
	Hypothetical protein	AD081_RS05635
	Phage major tail protein, TP901-1 family	AD081_RS05640
	Hypothetical protein	AD081_RS05645
	Phage tail tape measure protein	AD081_RS05650
	Phage tail family protein	AD081_RS05660
	DUF3037 domain-containing protein	AD081_RS05665
	Hypothetical protein	AD081_RS05770
	Hypothetical protein	AD081_RS05915
	Hypothetical protein	AD081_RS05920
	Hypothetical protein	AD081_RS05925
	IS256 family transposase	AD081_RS05930
	IS110 family transposase	AD081_RS14930
	Hypothetical protein	AD081_RS15375
	Ig-like domain-containing protein	AD081_RS15430
	Hypothetical protein	AD081_RS15755
	Hypothetical protein	AD081_RS16465
	hypothetical protein	AD081_RS16485

Table S4. List of strain-specific genes derived from the Venn diagram results

Strain	Product	Gene Locus
CACC 558	Hypothetical protein	GWD03_RS04170
	Hypothetical protein	GWD03_RS04980
	Hypothetical protein	GWD03_RS06550
	IS200/IS605 family transposase	GWD03_RS07315
	GH25 family lysozyme	GWD03_RS08215
	Hypothetical protein	GWD03_RS08220
	Hypothetical protein	GWD03_RS08290
	Hypothetical protein	GWD03_RS08345
	Hypothetical protein	GWD03_RS08360
	Helix-turn-helix transcriptional regulator	GWD03_RS08370
	YjzC family protein	GWD03_RS08375
	Hypothetical protein	GWD03_RS08455
	Hypothetical protein	GWD03_RS08460
	Hypothetical protein	GWD03_RS08465
	Hypothetical protein	GWD03_RS08470
	AAA family ATPase	GWD03_RS08490
	Hypothetical protein	GWD03_RS08495
	Hypothetical protein	GWD03_RS08500
	Hypothetical protein	GWD03_RS12930
	Helix-turn-helix domain-containing protein	GWD03_RS13345
	Hypothetical protein	GWD03_RS13355
	Hypothetical protein	GWD03_RS13370
	Hypothetical protein	GWD03_RS13375
	DUF3800 domain-containing protein	GWD03_RS13420
	Hypothetical protein	GWD03_RS14900
	Glycohydrolase toxin TNT-related protein	GWD03_RS14970
	DUF3800 domain-containing protein	GWD03_RS15190
	Hypothetical protein	GWD03_RS15195
	Hypothetical protein	GWD03_RS15200
	Y-family DNA polymerase	GWD03_RS15205
	Putative holin-like toxin	GWD03_RS15210
	DUF5706 domain-containing protein	GWD03_RS15215
	Adenylate/guanylate cyclase domain-containing protein	GWD03_RS15220
	MarR family transcriptional regulator	GWD03_RS15260
	Recombinase family protein	GWD03_RS15265
	IS5-like element ISLpl3 family transposase	GWD03_RS15315
	Cation:proton antiporter	GWD03_RS15320
	ClC family H(+)/Cl(-) exchange transporter	GWD03_RS15325
	IS110 family transposase	GWD03_RS15335
	LysM domain-containing protein	GWD03_RS15340
	IS30 family transposase	GWD03_RS15345
	Glycine betaine/L-proline ABC transporter ATP-binding protein	GWD03_RS15350
	Proline/glycine betaine ABC transporter permease	GWD03_RS15355
	Glycine/betaine ABC transporter	GWD03_RS15360
	IS30 family transposase	GWD03_RS15365
	Hypothetical protein	GWD03_RS15385
	Hypothetical protein	GWD03_RS15415
	Hypothetical protein	GWD03_RS15420
	Thioredoxin-disulfide reductase	GWD03_RS15425
	Thioredoxin family protein	GWD03_RS15430
	ArsR family transcriptional regulator	GWD03_RS15435
	MucBP domain-containing protein	GWD03_RS15440
	Hypothetical protein	GWD03_RS15445
	PTS system mannose/fructose/sorbose family transporter subunit IID	GWD03_RS15450
	PTS sugar transporter subunit IIC	GWD03_RS15455
	PTS sugar transporter subunit IIB	GWD03_RS15460

Strain	Product	Gene Locus
	Phosphotransferase system, mannose/fructose-specific component IIA	GWD03_RS15465
	PRD domain-containing protein	GWD03_RS15470
	Recombinase family protein	GWD03_RS15475
	IS30 family transposase	GWD03_RS15480
	APC family permease	GWD03_RS15485
	Recombinase family protein	GWD03_RS15495
	Hypothetical protein	GWD03_RS15500
	Hypothetical protein	GWD03_RS15510
	Methyltransferase	GWD03_RS15515
	Hypothetical protein	GWD03_RS15525
	Hypothetical protein	GWD03_RS15590
	Ribonuclease G	GWD03_RS15610
	IS30 family transposase	GWD03_RS15615
	OsmC family protein	GWD03_RS15620
	OsmC family protein	GWD03_RS15625
	Winged helix-turn-helix transcriptional regulator	GWD03_RS15630
	Hypothetical protein	GWD03_RS15635
	NAD(P)/FAD-dependent oxidoreductase	GWD03_RS15645
	Thioredoxin	GWD03_RS15650
	DsbA family oxidoreductase	GWD03_RS15655
E2	SpaA isopeptide-forming pilin-related protein	OLJ37_00805
	Hypothetical protein	OLJ37_01665
	IS256 family transposase	OLJ37_01680
	Helix-turn-helix domain-containing protein	OLJ37_02270
	Hypothetical protein	OLJ37_02275
	Rha family transcriptional regulator	OLJ37_02280
	Hypothetical protein	OLJ37_02285
	Hypothetical protein	OLJ37_02290
	Hypothetical protein	OLJ37_02295
	Bifunctional DNA primase/polymerase	OLJ37_02300
	Virulence protein	OLJ37_02305
	Hypothetical protein	OLJ37_02310
	Hypothetical protein	OLJ37_02315
	Hypothetical protein	OLJ37_02320
	DUF3021 domain-containing protein	OLJ37_02325
	LytTR family transcriptional regulator	OLJ37_02330
	Hypothetical protein	OLJ37_02335
	TIGR02328 family protein	OLJ37_02340
	YbgA family protein	OLJ37_02345
	Site-specific integrase	OLJ37_02445
	Hypothetical protein	OLJ37_02450
	Hypothetical protein	OLJ37_02485
	Hypothetical protein	OLJ37_02540
	Hypothetical protein	OLJ37_02545
	Hypothetical protein	OLJ37_02550
	DUF2971 domain-containing protein	OLJ37_02560
	Hypothetical protein	OLJ37_02565
	Clp protease ClpP	OLJ37_02605
	Phage tail tape measure protein	OLJ37_02650
	Hypothetical protein	OLJ37_02655
	DUF1617 family protein	OLJ37_02675
	Hypothetical protein	OLJ37_02680
	Hypothetical protein	OLJ37_02685
	Hypothetical protein	OLJ37_02690
	IS30-like element ISLsa1 family transposase	OLJ37_03115
	Hypothetical protein	OLJ37_03920
	IS30-like element ISLsa1 family transposase	OLJ37_03935
	Hypothetical protein	OLJ37_04020

Strain	Product	Gene Locus
	GtrA family protein	OLJ37_05010
	Hypothetical protein	OLJ37_05015
	Hypothetical protein	OLJ37_05020
	Hypothetical protein	OLJ37_05025
	Glycosyltransferase	OLJ37_05035
	Glycosyltransferase	OLJ37_05040
	Glycosyltransferase family 2 protein	OLJ37_05045
	Glycosyltransferase family 2 protein	OLJ37_05060
	Hypothetical protein	OLJ37_05065
	Glycosyltransferase	OLJ37_05070
	Hypothetical protein	OLJ37_05095
	Flippase	OLJ37_05115
	Hypothetical protein	OLJ37_05695
	Hypothetical protein	OLJ37_05710
	Hypothetical protein	OLJ37_05725
	DUF1351 domain-containing protein	OLJ37_05750
	ERF family protein	OLJ37_05755
	Single-stranded DNA-binding protein	OLJ37_05760
	ATP-binding protein	OLJ37_05775
	Hypothetical protein	OLJ37_05780
	Hypothetical protein	OLJ37_05795
	DUF1642 domain-containing protein	OLJ37_05800
	Hypothetical protein	OLJ37_05815
	Hypothetical protein	OLJ37_05935
	ABC transporter permease subunit	OLJ37_07490
	Transposase	OLJ37_07820
	Hypothetical protein	OLJ37_07835
	Transposase	OLJ37_09215
	Type II secretion system GspH family protein	OLJ37_09690
	DUF2829 domain-containing protein	OLJ37_10460
	Endodeoxyribonuclease	OLJ37_10485
	Single-stranded DNA-binding protein	OLJ37_10500
	ERF family protein	OLJ37_10505
	Helix-turn-helix domain-containing protein	OLJ37_10555
	Hypothetical protein	OLJ37_10595
	Hypothetical protein	OLJ37_10600
	Hypothetical protein	OLJ37_10605
	Hypothetical protein	OLJ37_10635
	DNA cytosine methyltransferase	OLJ37_10660
	DUF262 domain-containing protein	OLJ37_10665
	MAE_28990/MAE_18760 family HEPN-like nuclease	OLJ37_10670
	Hypothetical protein	OLJ37_10695
	Hypothetical protein	OLJ37_10725
	Hypothetical protein	OLJ37_10750
	Hypothetical protein	OLJ37_10755
	Hypothetical protein	OLJ37_10770
	Helix-turn-helix domain-containing protein	OLJ37_10780
	Hypothetical protein	OLJ37_10825
	LPXTG cell wall anchor domain-containing protein	OLJ37_11220
	Hypothetical protein	OLJ37_11410
	LamG domain-containing protein	OLJ37_12850
	IS30 family transposase	OLJ37_13095
	TfoX/Sxy family protein	OLJ37_13100
	Transposase	OLJ37_13715
	Transposase	OLJ37_14200
	Hypothetical protein	OLJ37_14590
	IS256 family transposase	OLJ37_15285
	Hypothetical protein	OLJ37_15580

Strain	Product	Gene Locus
	AAA family ATPase	OLJ37_15585
	Primase C-terminal domain-containing protein	OLJ37_15590
	LysR family transcriptional regulator	OLJ37_15600
	FAD-dependent oxidoreductase	OLJ37_15605
	Anion permease	OLJ37_15610
	Zeta toxin family protein	OLJ37_15620
	Hypothetical protein	OLJ37_15625
	Hypothetical protein	OLJ37_15630
	MobA/MobL family protein	OLJ37_15635
	Hypothetical protein	OLJ37_15640
	Hypothetical protein	OLJ37_15645
	CagC family type IV secretion system protein	OLJ37_15650
	Hypothetical protein	OLJ37_15655
	TrsD/TraD family conjugative transfer protein	OLJ37_15660
	DUF87 domain-containing protein	OLJ37_15665
	Type VII secretion protein EssB/YukC	OLJ37_15670
	Phage tail tip lysozyme	OLJ37_15675
	Hypothetical protein	OLJ37_15680
	Thioredoxin family protein	OLJ37_15685
	Hypothetical protein	OLJ37_15690
	Type IV secretory system conjugative DNA transfer family protein	OLJ37_15695
	Hypothetical protein	OLJ37_15700
	Hypothetical protein	OLJ37_15705
	Hypothetical protein	OLJ37_15710
	DNA (cytosine-5-)-methyltransferase	OLJ37_15720
	ParB/RepB/Spo0J family partition protein	OLJ37_15730
	SNF2-related protein	OLJ37_15735
	MutH/Sau3AI family endonuclease	OLJ37_15740
	Very short patch repair endonuclease	OLJ37_15745
	Hypothetical protein	OLJ37_15750
	Hypothetical protein	OLJ37_15770
	Hydantoinase/oxoprolinase family protein	OLJ37_15775
	DUF917 domain-containing protein	OLJ37_15780
	Cytosine permease	OLJ37_15785
	PucR family transcriptional regulator ligand-binding domain-containing protein	OLJ37_15790
	LysR family transcriptional regulator	OLJ37_15795
	Hypothetical protein	OLJ37_15800
	Hypothetical protein	OLJ37_15810
	Multicopper oxidase domain-containing protein	OLJ37_15815
	MFS transporter	OLJ37_15820
	Recombinase family protein	OLJ37_15825
	Amino acid permease	OLJ37_15830
	Hypothetical protein	OLJ37_15855
	Hypothetical protein	OLJ37_15860
	Type IA DNA topoisomerase	OLJ37_15870
	FAD-dependent oxidoreductase	OLJ37_15890
	Metalloregulator ArsR/SmtB family transcription factor	OLJ37_15895
	Arsenic transporter	OLJ37_15900
	MarR family transcriptional regulator	OLJ37_15950
	Recombinase family protein	OLJ37_15955
	IS30-like element ISLpl1 family transposase	OLJ37_15965
	GH25 family lysozyme	OLJ37_15970
	IS3 family transposase	OLJ37_15975
	IS3 family transposase	OLJ37_15980
	Hypothetical protein	OLJ37_16000
	5-oxoprolinase subunit PxpB	OLJ37_16015
	Biotin-dependent carboxyltransferase	OLJ37_16020

Strain	Product	Gene Locus
	Acetyl-CoA carboxylase biotin carboxyl carrier protein subunit	OLJ37_16025
	Acetyl-CoA carboxylase biotin carboxylase subunit	OLJ37_16030
	Putative hydro-lyase	OLJ37_16035
	LamB/YcsF family protein	OLJ37_16040
	Divalent metal cation transporter	OLJ37_16045
	Transposase	OLJ37_16050
	ISL3 family transposase	OLJ37_16055
	IS256 family transposase	OLJ37_16060
	Amino acid permease	OLJ37_16065
	DNA topoisomerase	OLJ37_16080
	MobQ family relaxase	OLJ37_16085
	BRCT domain-containing protein	OLJ37_16115
	ParA family protein	OLJ37_16120
	Hypothetical protein	OLJ37_16125
	Recombinase family protein	OLJ37_16175
	MarR family transcriptional regulator	OLJ37_16180
	KUP/HAK/KT family potassium transporter	OLJ37_16185
	Helix-turn-helix domain-containing protein	OLJ37_16190
	IS3 family transposase	OLJ37_16195
	IS3 family transposase	OLJ37_16200
	Nicotinamide riboside transporter PnuC	OLJ37_16205
	IS30-like element ISLpl1 family transposase	OLJ37_16215
	Carbohydrate ABC transporter permease	OLJ37_16220
	Sugar ABC transporter permease	OLJ37_16225
	ABC transporter substrate-binding protein	OLJ37_16230
	ABC transporter ATP-binding protein	OLJ37_16235
	Hypothetical protein	OLJ37_16245
	Hypothetical protein	OLJ37_16250
	Hypothetical protein	OLJ37_16265
	Hypothetical protein	OLJ37_16270
	IS256 family transposase	OLJ37_16275
	Oleate hydratase	OLJ37_16280
	IS30-like element ISLpl1 family transposase	OLJ37_16285
	Oleate hydratase	OLJ37_16305
	Hypothetical protein	OLJ37_16310
	TetR/AcrR family transcriptional regulator	OLJ37_16315
	Metallophosphoesterase	OLJ37_16325
	IS30 family transposase	OLJ37_16330
	YdhK family protein	OLJ37_16335
	Recombinase family protein	OLJ37_16350
	Hypothetical protein	OLJ37_16380
	Fic family protein	OLJ37_16385
	AAA family ATPase	OLJ37_16395
	DUF5388 domain-containing protein	OLJ37_16400
	Hypothetical protein	OLJ37_16405
	IS3-like element IS1163 family transposase	OLJ37_16410
	Recombinase family protein	OLJ37_16420
	MarR family transcriptional regulator	OLJ37_16425
	LPXTG cell wall anchor domain-containing protein	OLJ37_16430
	Hypothetical protein	OLJ37_16435
	VanZ family protein	OLJ37_16440
	IS256 family transposase	OLJ37_16445
	Sugar transferase	OLJ37_16450
	IS66 family insertion sequence element accessory protein TnpB	OLJ37_16465
	Hypothetical protein	OLJ37_16470
	Glycosyltransferase family 2 protein	OLJ37_16480
	Hypothetical protein	OLJ37_16485
	Acyltransferase	OLJ37_16495

Strain	Product	Gene Locus
	Glycosyltransferase	OLJ37_16500
	Glycosyltransferase	OLJ37_16510
	Hypothetical protein	OLJ37_16545
	Hypothetical protein	OLJ37_16550
	Hypothetical protein	OLJ37_16555
	Site-specific integrase	OLJ37_16560
	Type II toxin-antitoxin system prevent-host-death family antitoxin	OLJ37_16565
	Type II toxin-antitoxin system YafQ family toxin	OLJ37_16570
	Helix-turn-helix domain-containing protein	OLJ37_16575
	AraC family transcriptional regulator	OLJ37_16580
	Type I glyceraldehyde-3-phosphate dehydrogenase	OLJ37_16585
	Helix-turn-helix transcriptional regulator	OLJ37_16590
	DUF536 domain-containing protein	OLJ37_16595
	Protein rep	OLJ37_16600
	Hypothetical protein	OLJ37_16605
	Plasmid recombination protein	OLJ37_16610
FBL-3a	Phosphotransferase	EEJ46_RS00025
	Signal peptidase I	EEJ46_RS00255
	Hypothetical protein	EEJ46_RS00290
	Dihydrofolate reductase	EEJ46_RS00295
	ImmA/IrrE family metallo-endopeptidase	EEJ46_RS01880
	Helix-turn-helix domain-containing protein	EEJ46_RS01885
	Helix-turn-helix transcriptional regulator	EEJ46_RS01890
	Host-nuclease inhibitor Gam family protein	EEJ46_RS01925
	Hypothetical protein	EEJ46_RS01930
	Hypothetical protein	EEJ46_RS02110
	S8 family serine peptidase	EEJ46_RS02115
	AAA family ATPase	EEJ46_RS02120
	Hypothetical protein	EEJ46_RS02130
	LytTR family transcriptional regulator	EEJ46_RS03205
	DUF3021 domain-containing protein	EEJ46_RS03210
	ABC transporter ATP-binding protein	EEJ46_RS03675
	Site-specific integrase	EEJ46_RS03935
	Hypothetical protein	EEJ46_RS03940
	Cation diffusion facilitator family transporter	EEJ46_RS05790
	YhgE/Pip family protein	EEJ46_RS05795
	TetR/AcrR family transcriptional regulator	EEJ46_RS05800
	Helix-turn-helix transcriptional regulator	EEJ46_RS06075
	DUF2975 domain-containing protein	EEJ46_RS06080
	VTT domain-containing protein	EEJ46_RS06420
	Glycosyltransferase family 2 protein	EEJ46_RS06425
	Phosphatase PAP2 family protein	EEJ46_RS06430
	Response regulator transcription factor	EEJ46_RS06435
	HAMP domain-containing sensor histidine kinase	EEJ46_RS06440
	Hypothetical protein	EEJ46_RS06680
	Hypothetical protein	EEJ46_RS06720
	Phage tail protein	EEJ46_RS06725
	DUF806 family protein	EEJ46_RS06730
	Hypothetical protein	EEJ46_RS06735
	Hypothetical protein	EEJ46_RS06815
	Helix-turn-helix domain-containing protein	EEJ46_RS06830
	Host-nuclease inhibitor Gam family protein	EEJ46_RS06850
	Helix-turn-helix transcriptional regulator	EEJ46_RS06890
	LexA family transcriptional regulator	EEJ46_RS06895
	Hypothetical protein	EEJ46_RS06900
	Hypothetical protein	EEJ46_RS06920
	Hypothetical protein	EEJ46_RS06925
	Trypsin-like peptidase domain-containing protein	EEJ46_RS07135

Strain	Product	Gene Locus
	YitT family protein	EEJ46_RS07145
	M20/M25/M40 family metallo-hydrolase	EEJ46_RS07150
	Oligosaccharide flippase family protein	EEJ46_RS07605
	EpsG family protein	EEJ46_RS07615
	Glycosyltransferase	EEJ46_RS07620
	CDP-glycerol glycerophosphotransferase family protein	EEJ46_RS07625
	DUF1792 domain-containing protein	EEJ46_RS07630
	Glycosyltransferase	EEJ46_RS07635
	Cellulase family glycosylhydrolase	EEJ46_RS07695
	Hypothetical protein	EEJ46_RS07700
	O-antigen ligase family protein	EEJ46_RS07705
	Glycosyltransferase	EEJ46_RS07710
	DUF1972 domain-containing protein	EEJ46_RS07715
	Acyltransferase	EEJ46_RS07720
	CDP-glycerol glycerophosphotransferase family protein	EEJ46_RS07725
	Glycosyltransferase	EEJ46_RS07730
	Serine hydrolase	EEJ46_RS07735
	Hypothetical protein	EEJ46_RS07740
	Hypothetical protein	EEJ46_RS10060
	Hypothetical protein	EEJ46_RS10070
	Hypothetical protein	EEJ46_RS10075
	Class IIb bacteriocin, lactobin A/cerein 7B family	EEJ46_RS10800
	Transposase	EEJ46_RS10815
	Transposase	EEJ46_RS10820
	Transposase	EEJ46_RS10825
	Hypothetical protein	EEJ46_RS10830
	Hypothetical protein	EEJ46_RS10835
	Hypothetical protein	EEJ46_RS10875
	Hypothetical protein	EEJ46_RS10890
	Hypothetical protein	EEJ46_RS10945
	Hypothetical protein	EEJ46_RS10955
	Type II-A CRISPR-associated protein Csn2	EEJ46_RS12080
	CRISPR-associated endonuclease Cas2	EEJ46_RS12085
	Type II CRISPR-associated endonuclease Cas1	EEJ46_RS12090
	Type II CRISPR RNA-guided endonuclease Cas9	EEJ46_RS12095
	DUF4111 domain-containing protein	EEJ46_RS12210
	Hypothetical protein	EEJ46_RS12415
	AAA family ATPase	EEJ46_RS12420
	PTS sugar transporter subunit IIA	EEJ46_RS12870
	Helix-turn-helix domain-containing protein	EEJ46_RS13620
	Hypothetical protein	EEJ46_RS13625
	VanZ family protein	EEJ46_RS13630
	Hypothetical protein	EEJ46_RS13635
	SH3 domain-containing protein	EEJ46_RS14670
	Amidohydrolase family protein	EEJ46_RS14695
	Alpha/beta fold hydrolase	EEJ46_RS14700
	TetR/AcrR family transcriptional regulator	EEJ46_RS14705
	Hypothetical protein	EEJ46_RS14885
	Hypothetical protein	EEJ46_RS14890
	SDR family oxidoreductase	EEJ46_RS15025
	Hypothetical protein	EEJ46_RS15725
	Hypothetical protein	EEJ46_RS15740
	Hypothetical protein	EEJ46_RS15780
SRCM210465	Bacterial Ig-like domain-containing protein	NYR24_RS00310
	Helix-turn-helix transcriptional regulator	NYR24_RS00320
	Transposase	NYR24_RS00360
	IS3 family transposase	NYR24_RS02085
	Zinc-binding dehydrogenase	NYR24_RS02150

Strain	Product	Gene Locus
	Hypothetical protein	NYR24_RS02975
	Hypothetical protein	NYR24_RS04745
	Hypothetical protein	NYR24_RS05735
	DUF916 domain-containing protein	NYR24_RS05900
	Transposase	NYR24_RS06310
	Hypothetical protein	NYR24_RS07325
	Arm DNA-binding domain-containing protein	NYR24_RS07895
	Hypothetical protein	NYR24_RS07905
	Lytic polysaccharide monooxygenase	NYR24_RS07910
	Hypothetical protein	NYR24_RS07915
	Hypothetical protein	NYR24_RS07920
	Hypothetical protein	NYR24_RS07925
	GH25 family lysozyme	NYR24_RS07935
	XkdX family protein	NYR24_RS07940
	DUF2977 domain-containing protein	NYR24_RS07945
	Hypothetical protein	NYR24_RS07950
	Phage baseplate upper protein	NYR24_RS07955
	SGNH/GDSL hydrolase family protein	NYR24_RS07960
	Phage tail spike protein	NYR24_RS07965
	Phage tail protein	NYR24_RS07970
	Tape measure protein	NYR24_RS07975
	Hypothetical protein	NYR24_RS07980
	Hypothetical protein	NYR24_RS07985
	Tail assembly chaperone	NYR24_RS07990
	Phage tail protein	NYR24_RS07995
	Hypothetical protein	NYR24_RS08000
	Phage tail protein	NYR24_RS08005
	Hypothetical protein	NYR24_RS08010
	Phage head-tail connector protein	NYR24_RS08015
	Major capsid protein	NYR24_RS08020
	DUF4355 domain-containing protein	NYR24_RS08025
	Minor capsid protein	NYR24_RS08030
	Phage portal protein	NYR24_RS08035
	PBSX family phage terminase large subunit	NYR24_RS08040
	MarR family winged helix-turn-helix transcriptional regulator	NYR24_RS08050
	ATP-binding protein	NYR24_RS08070
	Hypothetical protein	NYR24_RS08100
	Hypothetical protein	NYR24_RS08105
	Hypothetical protein	NYR24_RS08110
	Hypothetical protein	NYR24_RS08115
	Helix-turn-helix transcriptional regulator	NYR24_RS08135
	Hypothetical protein	NYR24_RS08155
	ATP-binding protein	NYR24_RS08170
	DNA cytosine methyltransferase	NYR24_RS08175
	Site-specific integrase	NYR24_RS08180
	HAD-IC family P-type ATPase	NYR24_RS09210
	Serine hydrolase	NYR24_RS09995
	Acyltransferase	NYR24_RS10130
	Oligosaccharide flippase family protein	NYR24_RS10135
	Stealth CR1 domain-containing protein	NYR24_RS10140
	Glycosyltransferase	NYR24_RS10145
	Stealth conserved region 3 domain-containing protein	NYR24_RS10150
	Hypothetical protein	NYR24_RS10215
	AAA family ATPase	NYR24_RS10220
	Hypothetical protein	NYR24_RS10225
	Hypothetical protein	NYR24_RS10230
	Glycosyltransferase	NYR24_RS10245
	Stealth CR1 domain-containing protein	NYR24_RS10250

Strain	Product	Gene Locus
	Glycosyltransferase	NYR24_RS10255
	Hypothetical protein	NYR24_RS10260
	IS3 family transposase	NYR24_RS10265
	Helix-turn-helix domain-containing protein	NYR24_RS10275
	Glycosyltransferase	NYR24_RS10280
	Ribosome recycling factor	NYR24_RS10475
	Hypothetical protein	NYR24_RS11330
	Hypothetical protein	NYR24_RS11460
	Hypothetical protein	NYR24_RS12395
	Hypothetical protein	NYR24_RS12400
	Glycosyl hydrolase family 28-related protein	NYR24_RS12405
	Hypothetical protein	NYR24_RS12410
	PepSY domain-containing protein	NYR24_RS12415
	Phage tail tape measure protein	NYR24_RS12430
	Hypothetical protein	NYR24_RS12435
	Hypothetical protein	NYR24_RS12440
	Hypothetical protein	NYR24_RS12445
	Hypothetical protein	NYR24_RS12450
	Hypothetical protein	NYR24_RS12455
	Hypothetical protein	NYR24_RS12460
	Hypothetical protein	NYR24_RS12465
	Hypothetical protein	NYR24_RS12470
	N4-gp56 family major capsid protein	NYR24_RS12475
	Hypothetical protein	NYR24_RS12480
	Hypothetical protein	NYR24_RS12485
	Hypothetical protein	NYR24_RS12490
	PBSX family phage terminase large subunit	NYR24_RS12500
	Helix-turn-helix domain-containing protein	NYR24_RS12505
	Hypothetical protein	NYR24_RS12510
	Hypothetical protein	NYR24_RS12525
	Hypothetical protein	NYR24_RS12530
	Hypothetical protein	NYR24_RS12535
	Hypothetical protein	NYR24_RS12555
	DNA cytosine methyltransferase	NYR24_RS12560
	Hypothetical protein	NYR24_RS12605
	Hypothetical protein	NYR24_RS12610
	Hypothetical protein	NYR24_RS12625
	Helix-turn-helix transcriptional regulator	NYR24_RS12630
	Helix-turn-helix transcriptional regulator	NYR24_RS12635
	Hypothetical protein	NYR24_RS12640
	DUF5067 domain-containing protein	NYR24_RS12645
	Tyrosine-type recombinase/integrase	NYR24_RS12695
	Hypothetical protein	NYR24_RS12900
	Hypothetical protein	NYR24_RS12905
	Hypothetical protein	NYR24_RS13010
	Hypothetical protein	NYR24_RS13015
	HNH endonuclease	NYR24_RS13025
	Hypothetical protein	NYR24_RS13035
	Hypothetical protein	NYR24_RS13090
	Hypothetical protein	NYR24_RS13095
	Hypothetical protein	NYR24_RS13105
	ImmA/IrrE family metallo-endopeptidase	NYR24_RS13130
	Universal stress protein	NYR24_RS13155
	GIY-YIG nuclease family protein	NYR24_RS13160
	Hypothetical protein	NYR24_RS13600
	GH-E family nuclease	NYR24_RS13625
	Hypothetical protein	NYR24_RS13930
	Hypothetical protein	NYR24_RS13935

Strain	Product	Gene Locus
	Hypothetical protein	NYR24_RS14140
W2	Hypothetical protein	L1599_RS00110
	Hypothetical protein	L1599_RS00625
	Hypothetical protein	L1599_RS03560
	Hypothetical protein	L1599_RS04765
	Helix-turn-helix domain-containing protein	L1599_RS04770
	DMT family transporter	L1599_RS05445
	Hypothetical protein	L1599_RS07120
	ISL3 family transposase	L1599_RS07295
	Hypothetical protein	L1599_RS07990
	ISL3-like element ISP1 family transposase	L1599_RS08030
	Hypothetical protein	L1599_RS08590
	Hypothetical protein	L1599_RS08705
	Holin	L1599_RS10160
	Hemolysin XhlA family protein	L1599_RS10165
	GH25 family lysozyme	L1599_RS10170
	Capsid protein	L1599_RS10245
	PBSX family phage terminase large subunit	L1599_RS10265
	Phage antirepressor KilAC domain-containing protein	L1599_RS10325
	Hypothetical protein	L1599_RS10360
	Hypothetical protein	L1599_RS10395
	DNA/RNA non-specific endonuclease	L1599_RS10435
	Hypothetical protein	L1599_RS10455
	DUF3037 domain-containing protein	L1599_RS10460
	KxYKxGKxW signal peptide domain-containing protein	L1599_RS11665
	Hypothetical protein	L1599_RS13200
	Hypothetical protein	L1599_RS13205
	Hypothetical protein	L1599_RS13575
	IS30 family transposase	L1599_RS15010
	Restriction endonuclease subunit S	L1599_RS15335
10CH	Acetyl-CoA carboxylase biotin carboxyl carrier protein subunit	B0667_RS00190
	Hypothetical protein	B0667_RS00195
	TatD family hydrolase	B0667_RS00200
	Transposase	B0667_RS00205
	Thioesterase	B0667_RS00210
	Transporter substrate-binding domain-containing protein	B0667_RS00215
	DNA replication initiation control protein YabA	B0667_RS01655
	Endodeoxyribonuclease	B0667_RS01660
	Y-family DNA polymerase	B0667_RS01675
	YfhO family protein	B0667_RS01680
	YfhO family protein	B0667_RS01685
	YhgE/Pip domain-containing protein	B0667_RS01690
	YjdF family protein	B0667_RS01695
	YopX family protein	B0667_RS01715
	Hypothetical protein	B0667_RS01775
	Hypothetical protein	B0667_RS01780
	Helix-turn-helix transcriptional regulator	B0667_RS01785
	YjzC family protein	B0667_RS01790
	Hypothetical protein	B0667_RS01795
	Hypothetical protein	B0667_RS01800
	Type IA DNA topoisomerase	B0667_RS03815
	Trypsin-like peptidase domain-containing protein	B0667_RS03820
	Hypothetical protein	B0667_RS03825
	Hypothetical protein	B0667_RS03830
	Hypothetical protein	B0667_RS03840
	APC family permease	B0667_RS04495
	Cof-type HAD-IIB family hydrolase	B0667_RS04830
	Cyclic-di-AMP receptor	B0667_RS04875

Strain	Product	Gene Locus
	DNA polymerase III subunit delta	B0667_RS04880
	DUF771 domain-containing protein	B0667_RS04895
	DUF536 domain-containing protein	B0667_RS04915
	LacI family DNA-binding transcriptional regulator	B0667_RS04920
	Hypothetical protein	B0667_RS04950
	Hypothetical protein	B0667_RS04960
	DUF4868 domain-containing protein	B0667_RS04975
	DUF4430 domain-containing protein	B0667_RS04985
	DUF4428 domain-containing protein	B0667_RS04990
	Hypothetical protein	B0667_RS05255
	Type II toxin-antitoxin system RelB/DinJ family antitoxin	B0667_RS05265
	Hypothetical protein	B0667_RS05275
	ABC transporter ATP-binding protein	B0667_RS05285
	RluA family pseudouridine synthase	B0667_RS05330
	SsrA-binding protein SmpB	B0667_RS05530
	L(+)-tartrate dehydratase subunit beta	B0667_RS05875
	Hypothetical protein	B0667_RS05880
	L(+)-tartrate dehydratase subunit alpha	B0667_RS05885
	Putative hydro-lyase	B0667_RS05895
	DUF2325 domain-containing protein	B0667_RS06210
	ABC transporter ATP-binding protein	B0667_RS10755
	Cation:proton antiporter	B0667_RS10760
	Hypothetical protein	B0667_RS10780
	Transcriptional regulator	B0667_RS10885
	Hypothetical protein	B0667_RS11370
	Hypothetical protein	B0667_RS11375
	Hypothetical protein	B0667_RS11380
	Hypothetical protein	B0667_RS11385
	Hypothetical protein	B0667_RS11390
	Hemolysin family protein	B0667_RS12540
	LacI family DNA-binding transcriptional regulator	B0667_RS13110
	Hypothetical protein	B0667_RS13115
	Hypothetical protein	B0667_RS13120
	GHKL domain-containing protein	B0667_RS13125
	Polysaccharide deacetylase family protein	B0667_RS13135
	Biotin--[acetyl-CoA-carboxylase] ligase	B0667_RS13140
	Hypothetical protein	B0667_RS13145
	PTS glucitol/sorbitol transporter subunit IIC	B0667_RS13375
	DUF771 domain-containing protein	B0667_RS13380
	DUF1836 domain-containing protein	B0667_RS13475
	LytTR family DNA-binding domain-containing protein	B0667_RS13480
	Hypothetical protein	B0667_RS13550
	Hypothetical protein	B0667_RS13555
	DTMP kinase	B0667_RS13605
	Phosphoribosylformylglycinamidine synthase subunit PurS	B0667_RS13620
	Phosphoribosylformylglycinamidine synthase subunit PurQ	B0667_RS13625
	Glycerol kinase GlpK	B0667_RS13630
	AAA family ATPase	B0667_RS13635
	Hypothetical protein	B0667_RS13640
	Asp23/Gls24 family envelope stress response protein	B0667_RS13650
	Alpha-amylase family glycosyl hydrolase	B0667_RS13665
	Amino acid permease	B0667_RS13680
	Hypothetical protein	B0667_RS13685
	Hypothetical protein	B0667_RS13690
	IS256 family transposase	B0667_RS14030
	IS256 family transposase	B0667_RS14035
	IS256 family transposase	B0667_RS14040
	IS256 family transposase	B0667_RS14045

Strain	Product	Gene Locus
	IS256 family transposase	B0667_RS14050
	Hypothetical protein	B0667_RS15120
	ABC transporter permease	B0667_RS15125
	DEAD/DEAH box helicase family protein	B0667_RS15130
	Hypothetical protein	B0667_RS15135
	MFS transporter	B0667_RS15145
	FGGY-family carbohydrate kinase	B0667_RS15150
	ABC transporter permease	B0667_RS15280
	Spx/MgsR family RNA polymerase-binding regulatory protein	B0667_RS15285
	DUF669 domain-containing protein	B0667_RS15290
	Sugar ABC transporter permease	B0667_RS15295
	Sugar ABC transporter permease	B0667_RS15300
	FAD-dependent oxidoreductase	B0667_RS15315
	LysR family transcriptional regulator	B0667_RS15380
	LysR family transcriptional regulator	B0667_RS15385
	LysR family transcriptional regulator	B0667_RS15390
	LysR family transcriptional regulator	B0667_RS15395
	LysR family transcriptional regulator	B0667_RS15400
	LysR substrate-binding domain-containing protein	B0667_RS15405
	Lytic polysaccharide monooxygenase	B0667_RS15410
	Lytic polysaccharide monooxygenase	B0667_RS15415
	Magnesium transporter CorA family protein	B0667_RS15420
	Sugar ABC transporter	B0667_RS15565
	SemiSWEET family transporter	B0667_RS15570
	PTS sugar transporter subunit IIC	B0667_RS15590
	GH25 family lysozyme	B0667_RS15595
	GIY-YIG nuclease family protein	B0667_RS15600
	GNAT family N-acetyltransferase	B0667_RS15605
	YjjG family noncanonical pyrimidine nucleotidase	B0667_RS15610
	Hypothetical protein	B0667_RS15615
	NAD(P)-binding domain-containing protein	B0667_RS15620
	Hypothetical protein	B0667_RS15625
	Accessory gene regulator AgrB	B0667_RS15630
	Glucosamine-6-phosphate deaminase	B0667_RS15635
	Restriction endonuclease subunit S	B0667_RS15645
	SDR family oxidoreductase	B0667_RS15650
	Hypothetical protein	B0667_RS15655
	ABC transporter ATP-binding protein	B0667_RS15660
	Acyltransferase family protein	B0667_RS15665
	Hypothetical protein	B0667_RS15670
	OsmC family protein	B0667_RS15675
	RNA polymerase recycling motor HelD	B0667_RS15680
	Bacteriocin	B0667_RS15685
	Hypothetical protein	B0667_RS15690
	Zeta toxin family protein	B0667_RS15695
	YkuJ family protein	B0667_RS15975
	YdeI/OmpD-associated family protein	B0667_RS16020
	LysR family transcriptional regulator	B0667_RS16025
	Terminase small subunit	B0667_RS16065
	Hypothetical protein	B0667_RS16090
8P-A3	Hypothetical protein	GPJ69_RS00150
	MarR family winged helix-turn-helix transcriptional regulator	GPJ69_RS00230
	PTS galactitol transporter subunit IIC	GPJ69_RS00410
	Veg family protein	GPJ69_RS00980
	Uracil-DNA glycosylase	GPJ69_RS01030
	Hypothetical protein	GPJ69_RS01035
	Hypothetical protein	GPJ69_RS01040
	Peptide chain release factor N(5)-glutamine methyltransferase	GPJ69_RS01670

Strain	Product	Gene Locus
	SDR family oxidoreductase	GPJ69_RS01680
	Alpha/beta hydrolase-fold protein	GPJ69_RS01725
	Phosphoketolase family protein	GPJ69_RS01730
	Aspartate--ammonia ligase	GPJ69_RS01735
	Asparagine--tRNA ligase	GPJ69_RS01740
	FMN-dependent NADH-azoreductase	GPJ69_RS01745
	LysR family transcriptional regulator	GPJ69_RS01760
	Carboxymuconolactone decarboxylase family protein	GPJ69_RS02360
	MerR family transcriptional regulator	GPJ69_RS02365
	Hypothetical protein	GPJ69_RS02380
	Hypothetical protein	GPJ69_RS02385
	Hypothetical protein	GPJ69_RS02390
	NusG domain II-containing protein	GPJ69_RS02395
	Phosphate ABC transporter permease PstA	GPJ69_RS02400
	ABC transporter ATP-binding protein	GPJ69_RS02405
	Sugar O-acetyltransferase	GPJ69_RS02410
	50S ribosomal protein L18	GPJ69_RS02720
	MerR family transcriptional regulator	GPJ69_RS02750
	Sugar kinase	GPJ69_RS02755
	Alpha-galactosidase	GPJ69_RS02760
	YitT family protein	GPJ69_RS02765
	Serine hydroxymethyltransferase	GPJ69_RS02770
	Nitroreductase family protein	GPJ69_RS02775
	Nucleoside hydrolase	GPJ69_RS02780
	Polyphosphate kinase 2 family protein	GPJ69_RS02785
	SH3 domain-containing protein	GPJ69_RS02810
	Methionine ABC transporter ATP-binding protein	GPJ69_RS02835
	Response regulator transcription factor	GPJ69_RS02840
	DUF1934 domain-containing protein	GPJ69_RS02845
	Uracil phosphoribosyltransferase	GPJ69_RS02855
	FtsW/RodA/SpoVE family cell cycle protein	GPJ69_RS02860
	ISL3-like element ISP1 family transposase	GPJ69_RS02865
	DUF2969 domain-containing protein	GPJ69_RS02990
	Membrane protein insertion efficiency factor YidD	GPJ69_RS03000
	Rod shape-determining protein	GPJ69_RS03005
	PTS sugar transporter subunit IIB	GPJ69_RS03010
	GNAT family protein	GPJ69_RS03020
	UDP-N-acetylglucosamine 1-carboxyvinyltransferase	GPJ69_RS03025
	MerR family transcriptional regulator	GPJ69_RS03345
	Phage head-tail connector protein	GPJ69_RS03355
	IS3-like element IS1163 family transposase	GPJ69_RS03360
	ABC transporter permease	GPJ69_RS04110
	ABC transporter ATP-binding protein	GPJ69_RS04125
	CPBP family intramembrane metalloprotease	GPJ69_RS04130
	Phage portal protein	GPJ69_RS05210
	Hypothetical protein	GPJ69_RS05215
	Zinc-binding dehydrogenase	GPJ69_RS05220
	Hypothetical protein	GPJ69_RS05225
	Putative hydroxymethylpyrimidine transporter CytX	GPJ69_RS05230
	Nickel pincer cofactor biosynthesis protein LarB	GPJ69_RS05235
	Type I pantothenate kinase	GPJ69_RS05245
	F0F1 ATP synthase subunit beta	GPJ69_RS05250
	Helix-turn-helix domain-containing protein	GPJ69_RS05255
	Hypothetical protein	GPJ69_RS05260
	RNA polymerase recycling motor HelD	GPJ69_RS05265
	30S ribosomal protein S8	GPJ69_RS05270
	F0F1 ATP synthase subunit gamma	GPJ69_RS05275
	F0F1 ATP synthase subunit alpha	GPJ69_RS05280

Strain	Product	Gene Locus
	F0F1 ATP synthase subunit C	GPJ69_RS05285
	PTS glucose transporter subunit IIA	GPJ69_RS05295
	Hypothetical protein	GPJ69_RS05305
	Peptide chain release factor 1	GPJ69_RS05315
	Mannose-6-phosphate isomerase, class I	GPJ69_RS05320
	Ketohydroxyglutarate aldolase	GPJ69_RS05325
	Thymidine kinase	GPJ69_RS05330
	Hypothetical protein	GPJ69_RS05335
	Glutamine amidotransferase	GPJ69_RS05340
	GHKL domain-containing protein	GPJ69_RS05345
	Prepilin peptidase	GPJ69_RS05410
	50S ribosomal protein L22	GPJ69_RS05415
	50S ribosomal protein L6	GPJ69_RS05420
	DNA mismatch repair protein MutS	GPJ69_RS05425
	Serine hydrolase domain-containing protein	GPJ69_RS06030
	GntR family transcriptional regulator	GPJ69_RS06040
	Helix-turn-helix domain-containing protein	GPJ69_RS06065
	F0F1 ATP synthase subunit A	GPJ69_RS06095
	DMT family transporter	GPJ69_RS06140
	Tail assembly chaperone	GPJ69_RS06160
	DNA topoisomerase (ATP-hydrolyzing) subunit B	GPJ69_RS06185
	Cys-tRNA(Pro) deacylase	GPJ69_RS06190
	Hypothetical protein	GPJ69_RS06195
	Arsenate reductase (thioredoxin)	GPJ69_RS06200
	GntR family transcriptional regulator	GPJ69_RS06205
	Hypothetical protein	GPJ69_RS06265
	Heavy metal translocating P-type ATPase	GPJ69_RS06490
	Metallophosphoesterase	GPJ69_RS06525
	Hypothetical protein	GPJ69_RS06540
	PTS lactose/cellobiose transporter subunit IIA	GPJ69_RS07455
	Hypothetical protein	GPJ69_RS07595
	4-oxalocrotonate tautomerase	GPJ69_RS07925
	D-serine ammonia-lyase	GPJ69_RS08570
	Cadmium resistance transporter	GPJ69_RS08625
	Hypothetical protein	GPJ69_RS09565
	DUF488 family protein	GPJ69_RS10370
	Glycosyltransferase	GPJ69_RS10700
	MFS transporter	GPJ69_RS10710
	PTS transporter subunit IIC	GPJ69_RS10715
	Fructose PTS transporter subunit IIB	GPJ69_RS10725
	TetR/AcrR family transcriptional regulator	GPJ69_RS10740
	Transposase	GPJ69_RS10745
	Nicotinate phosphoribosyltransferase	GPJ69_RS10760
	Amino acid ABC transporter permease	GPJ69_RS10765
	Transcription repressor NadR	GPJ69_RS10770
	DUF871 domain-containing protein	GPJ69_RS10775
	SDR family NAD(P)-dependent oxidoreductase	GPJ69_RS10780
	DUF2829 domain-containing protein	GPJ69_RS10785
	Hypothetical protein	GPJ69_RS10790
	Ribosome-associated translation inhibitor RaiA	GPJ69_RS10795
	DHH family phosphoesterase	GPJ69_RS10800
	3-oxoacyl-ACP reductase	GPJ69_RS10805
	WxL domain-containing protein	GPJ69_RS10810
	HIT family protein	GPJ69_RS10815
	Hypothetical protein	GPJ69_RS10820
	NUDIX hydrolase	GPJ69_RS10825
	Glycoside hydrolase family 65 protein	GPJ69_RS10835
	NAD(P)/FAD-dependent oxidoreductase	GPJ69_RS10840

Strain	Product	Gene Locus
	Hypothetical protein	GPJ69_RS10845
	Signal peptidase I	GPJ69_RS10850
	Hypothetical protein	GPJ69_RS10870
	Solute carrier family 23 protein	GPJ69_RS10915
	Glycoside hydrolase family 38 C-terminal domain-containing protein	GPJ69_RS10920
	Hypothetical protein	GPJ69_RS10945
	Hypothetical protein	GPJ69_RS10970
	SpaA isopeptide-forming pilin-related protein	GPJ69_RS10985
	MFS transporter	GPJ69_RS11615
	Hypothetical protein	GPJ69_RS12570
	Hypothetical protein	GPJ69_RS12575
	Hypothetical protein	GPJ69_RS12585
	Hypothetical protein	GPJ69_RS12600
	Hypothetical protein	GPJ69_RS12605
	Hypothetical protein	GPJ69_RS12610
	Hypothetical protein	GPJ69_RS12615
	Hypothetical protein	GPJ69_RS12620
	Hypothetical protein	GPJ69_RS12625
	Hypothetical protein	GPJ69_RS12630
	Hypothetical protein	GPJ69_RS12635
	Hypothetical protein	GPJ69_RS12640
	Hypothetical protein	GPJ69_RS12650
	Helix-turn-helix transcriptional regulator	GPJ69_RS13720
	ImmA/IrrE family metallo-endopeptidase	GPJ69_RS13765
	Alpha-glucosidase	GPJ69_RS13995
	GNAT family N-acetyltransferase	GPJ69_RS14045
	NADH-dependent flavin oxidoreductase	GPJ69_RS14055
	DUF771 domain-containing protein	GPJ69_RS14060
	DUF5067 domain-containing protein	GPJ69_RS14065
	Hypothetical protein	GPJ69_RS14070
	IS30 family transposase	GPJ69_RS14385
	Amidophosphoribosyltransferase	GPJ69_RS14835
	Ldh family oxidoreductase	GPJ69_RS15250
	Leucocin A/sakacin P family class II bacteriocin	GPJ69_RS15255
	LicD family protein	GPJ69_RS15260
	MFS transporter	GPJ69_RS15480
	MFS transporter	GPJ69_RS15485
	MFS transporter	GPJ69_RS15490
	MFS transporter	GPJ69_RS15495
	MFS transporter	GPJ69_RS15500
	MFS transporter	GPJ69_RS15510
	MFS transporter	GPJ69_RS15515
	MFS transporter	GPJ69_RS15520
	MFS transporter	GPJ69_RS15525
	MFS transporter	GPJ69_RS15530
	MFS transporter	GPJ69_RS15535
	MFS transporter	GPJ69_RS15545
	Site-specific integrase	GPJ69_RS15850
	L-threonylcarbamoyladenylate synthase	GPJ69_RS15895
	ABC transporter ATP-binding protein	GPJ69_RS15950
	Phage tail tape measure protein	GPJ69_RS15955
	IS30-like element ISLsa1 family transposase	GPJ69_RS15975
LL441	Cell division protein ZapA	O4Z47_RS00095
	TetR/AcrR family transcriptional regulator	O4Z47_RS00575
	DUF1292 domain-containing protein	O4Z47_RS00635
	Holliday junction resolvase RuvX	O4Z47_RS00640
	AI-2E family transporter	O4Z47_RS00825
	Phosphate ABC transporter ATP-binding protein PstB	O4Z47_RS00840

Strain	Product	Gene Locus
	S-ribosylhomocysteine lyase	O4Z47_RS00980
	IS30-like element ISLpl1 family transposase	O4Z47_RS01150
	IS3 family transposase	O4Z47_RS01155
	IS30 family transposase	O4Z47_RS01160
	CvpA family protein	O4Z47_RS01270
	Alanine--tRNA ligase	O4Z47_RS01295
	Valine--tRNA ligase	O4Z47_RS01455
	GGDEF domain-containing protein	O4Z47_RS01665
	Replication-associated recombination protein A	O4Z47_RS01765
	Phage holin family protein	O4Z47_RS01855
	Excinuclease ABC subunit UvrA	O4Z47_RS01960
	2-amino-4-hydroxy-6- hydroxymethyldihydropteridine diphosphokinase	O4Z47_RS02435
	Central glycolytic genes regulator	O4Z47_RS02795
	Folylpolyglutamate synthase/dihydrofolate synthase family protein	O4Z47_RS02800
	DUF421 domain-containing protein	O4Z47_RS02970
	Dihydroneopterin aldolase	O4Z47_RS03005
	Oligosaccharide flippase family protein	O4Z47_RS03055
	Hypothetical protein	O4Z47_RS03060
	CBS domain-containing protein	O4Z47_RS03095
	DEAD/DEAH box helicase	O4Z47_RS03185
	Sensor histidine kinase KdpD	O4Z47_RS03195
	LysM peptidoglycan-binding domain-containing protein	O4Z47_RS03215
	Bifunctional oligoribonuclease/PAP phosphatase NrnA	O4Z47_RS03365
	CDP-glycerol glycerophosphotransferase family protein	O4Z47_RS03455
	Y-family DNA polymerase	O4Z47_RS03460
	VIT family protein	O4Z47_RS03980
	MalY/PatB family protein	O4Z47_RS04195
	LysR family transcriptional regulator	O4Z47_RS04200
	Hypothetical protein	O4Z47_RS04245
	DUF1054 domain-containing protein	O4Z47_RS04250
	DUF1398 family protein	O4Z47_RS04270
	Phosphate signaling complex protein PhoU	O4Z47_RS04305
	SDR family oxidoreductase	O4Z47_RS04310
	Hypothetical protein	O4Z47_RS04315
	Hypothetical protein	O4Z47_RS04345
	Hypothetical protein	O4Z47_RS04350
	DUF87 domain-containing protein	O4Z47_RS04360
	DNA polymerase IV	O4Z47_RS04435
	SDR family oxidoreductase	O4Z47_RS04445
	Hypothetical protein	O4Z47_RS04450
	Family 1 glycosylhydrolase	O4Z47_RS04465
	DUF3021 domain-containing protein	O4Z47_RS04485
	DNA polymerase III subunit gamma/tau	O4Z47_RS04490
	Preprotein translocase subunit YajC	O4Z47_RS04495
	TRNA guanosine(34) transglycosylase Tgt	O4Z47_RS05565
	TRNA preQ1(34) S-adenosylmethionine ribosyltransferase-isomerase QueA	O4Z47_RS05580
	Holliday junction branch migration DNA helicase RuvB	O4Z47_RS05585
	EndoU domain-containing protein	O4Z47_RS05605
	Sugar phosphate isomerase/epimerase	O4Z47_RS05610
	Universal stress protein	O4Z47_RS05615
	Tail protein	O4Z47_RS05620
	Type I 3-dehydroquinate dehydratase	O4Z47_RS05625
	Hypothetical protein	O4Z47_RS05660
	ABC transporter ATP-binding protein	O4Z47_RS05690
	Helix-turn-helix transcriptional regulator	O4Z47_RS05695
	Citrate lyase acyl carrier protein	O4Z47_RS05700
	Holliday junction branch migration protein RuvA	O4Z47_RS05725
	Hypothetical protein	O4Z47_RS05740

Strain	Product	Gene Locus
	30S ribosomal protein S4	O4Z47_RS05825
	YfhO family protein	O4Z47_RS05830
	Hypothetical protein	O4Z47_RS05855
	HAD family hydrolase	O4Z47_RS06035
	Metal ABC transporter permease	O4Z47_RS06125
	Hypothetical protein	O4Z47_RS06200
	ABC transporter ATP-binding protein	O4Z47_RS06545
	Glycosyltransferase family 2 protein	O4Z47_RS06815
	Thioredoxin family protein	O4Z47_RS07280
	Helix-turn-helix transcriptional regulator	O4Z47_RS07295
	Matrixin family metalloprotease	O4Z47_RS07480
	Ribosomal protein S18-alanine N-acetyltransferase	O4Z47_RS07835
	DNA mismatch repair endonuclease MutL	O4Z47_RS08380
	Hypothetical protein	O4Z47_RS08390
	YaaL family protein	O4Z47_RS08735
	Thiol peroxidase	O4Z47_RS08740
	Glycerol-3-phosphate cytidylyltransferase	O4Z47_RS08745
	DNA mismatch repair protein MutS	O4Z47_RS08750
	Glutamate--cysteine ligase	O4Z47_RS08830
	YbaB/EbfC family nucleoid-associated protein	O4Z47_RS08840
	TRNA uracil 4-sulfurtransferase ThiI	O4Z47_RS08845
	Multidrug efflux SMR transporter	O4Z47_RS09035
	Thioredoxin	O4Z47_RS09060
	TIGR00282 family metallophosphoesterase	O4Z47_RS09145
	Site-specific integrase	O4Z47_RS09400
	Cupin domain-containing protein	O4Z47_RS09435
	Branched-chain amino acid ABC transporter permease	O4Z47_RS09710
	Cyclophilin-like fold protein	O4Z47_RS09840
	VIT family protein	O4Z47_RS09850
	DNA/RNA helicase	O4Z47_RS09855
	Ribonuclease Y	O4Z47_RS09860
	Recombinase RecA	O4Z47_RS09870
	Carbonic anhydrase family protein	O4Z47_RS09880
	Competence/damage-inducible protein A	O4Z47_RS09895
	DUF3290 domain-containing protein	O4Z47_RS09955
	Site-specific integrase	O4Z47_RS09960
	SMR family transporter	O4Z47_RS10025
	Hemolysin III family protein	O4Z47_RS10230
	Cytosine permease	O4Z47_RS10370
	Branched-chain amino acid ABC transporter permease	O4Z47_RS10375
	RNA polymerase factor sigma-54	O4Z47_RS10445
	YigZ family protein	O4Z47_RS10600
	Hypothetical protein	O4Z47_RS10710
	GntR family transcriptional regulator	O4Z47_RS10950
	CDP-diacylglycerol--glycerol-3-phosphate 3-phosphatidyltransferase	O4Z47_RS10955
	Flippase	O4Z47_RS11350
	5-(carboxyamino)imidazole ribonucleotide mutase	O4Z47_RS11465
	EAL domain-containing protein	O4Z47_RS11485
	Peptide ABC transporter substrate-binding protein	O4Z47_RS11525
	NAD(P)H-binding protein	O4Z47_RS11730
	CopY/TcrY family copper transport repressor	O4Z47_RS11770
	MarR family transcriptional regulator	O4Z47_RS11775
	DNA-3-methyladenine glycosylase I	O4Z47_RS12030
	YxeA family protein	O4Z47_RS12040
	ClC family H(+)/Cl(-) exchange transporter	O4Z47_RS12105
	Helix-turn-helix domain-containing protein	O4Z47_RS12120
	DUF308 domain-containing protein	O4Z47_RS12125
	DUF805 domain-containing protein	O4Z47_RS12180

Strain	Product	Gene Locus
	Dihydropteroate synthase	O4Z47_RS12260
	Type Z 30S ribosomal protein S14	O4Z47_RS12265
	Bis(5-nucleosyl)-tetraphosphatase	O4Z47_RS12270
	50S ribosomal protein L9	O4Z47_RS12275
	Hypothetical protein	O4Z47_RS12280
	Helix-turn-helix transcriptional regulator	O4Z47_RS12290
	Acyltransferase	O4Z47_RS12295
	Septation ring formation regulator EzrA	O4Z47_RS12310
	Pitrilysin family protein	O4Z47_RS12350
	Non-canonical purine NTP pyrophosphatase	O4Z47_RS12360
	Pitrilysin family protein	O4Z47_RS12365
	Helix-turn-helix domain-containing protein	O4Z47_RS12790
	MFS transporter	O4Z47_RS12880
	Aspartate kinase	O4Z47_RS12885
	Hypothetical protein	O4Z47_RS13620
	Mur ligase family protein	O4Z47_RS13785
	Amino acid ABC transporter substrate-binding protein	O4Z47_RS13800
	Hypothetical protein	O4Z47_RS13930
	GTP cyclohydrolase I FolE	O4Z47_RS14110
	Hypothetical protein	O4Z47_RS14350
	Hypothetical protein	O4Z47_RS14355
	Hypothetical protein	O4Z47_RS14360
	TRNA uridine-5-carboxymethylaminomethyl(34) synthesis GTPase MnmE	O4Z47_RS14365
	Serine transporter	O4Z47_RS14370
	Hypothetical protein	O4Z47_RS14375
	Elongation factor G	O4Z47_RS14380
	Helix-turn-helix domain-containing protein	O4Z47_RS14385
	Hypothetical protein	O4Z47_RS14390
	SGNH/GDSL hydrolase family protein	O4Z47_RS14395
	LexA family transcriptional regulator	O4Z47_RS14405
	Alpha/beta fold hydrolase	O4Z47_RS14410
	Recombination mediator RecR	O4Z47_RS14415
	Amino acid ABC transporter ATP-binding protein	O4Z47_RS14420
	Multidrug efflux SMR transporter	O4Z47_RS14425
	FAD-dependent oxidoreductase	O4Z47_RS14430
	SPFH domain-containing protein	O4Z47_RS14435
	Aminotransferase class I/II-fold pyridoxal phosphate-dependent enzyme	O4Z47_RS14440
	TIGR02328 family protein	O4Z47_RS14445
	Amino acid ABC transporter permease	O4Z47_RS14450
	TetR/AcrR family transcriptional regulator	O4Z47_RS14455
	Alpha/beta hydrolase	O4Z47_RS14460
	PfkB family carbohydrate kinase	O4Z47_RS14465
	D-ribose pyranase	O4Z47_RS14470
	YueI family protein	O4Z47_RS14475
	PTS sugar transporter subunit IIA	O4Z47_RS14495
	Methionine synthase	O4Z47_RS14500
	Hypothetical protein	O4Z47_RS14520
	Phage portal protein	O4Z47_RS14525
	Septum site-determining protein MinD	O4Z47_RS14545
	HAD family hydrolase	O4Z47_RS14555
	Autorepressor SdpR family transcription factor	O4Z47_RS14640
	Septum site-determining protein MinC	O4Z47_RS14645
	Glutathione peroxidase	O4Z47_RS14650
	Aldo/keto reductase	O4Z47_RS14680
	Hypothetical protein	O4Z47_RS14685
	M20/M25/M40 family metallo-hydrolase	O4Z47_RS14690
	Hypothetical protein	O4Z47_RS14705
	Hypothetical protein	O4Z47_RS14720

Strain	Product	Gene Locus
	Alpha-glucosidase	O4Z47_RS14725
	Membrane protein	O4Z47_RS14735
	ABC transporter ATP-binding protein	O4Z47_RS14740
	Cysteine synthase family protein	O4Z47_RS14750
	L,D-transpeptidase	O4Z47_RS14790
	Cysteine desulfurase family protein	O4Z47_RS14795
	Beta-glucoside-specific PTS transporter subunit IIABC	O4Z47_RS14800
	DeoR/GlpR family DNA-binding transcription regulator	O4Z47_RS14805
	Dihydroorotate dehydrogenase	O4Z47_RS14810
	Phage tail tape measure protein	O4Z47_RS14825
	Type II toxin-antitoxin system prevent-host-death family antitoxin	O4Z47_RS14830
	Ldh family oxidoreductase	O4Z47_RS14835
	DUF1648 domain-containing protein	O4Z47_RS14840
	Hypothetical protein	O4Z47_RS14855
	Glutamine-hydrolyzing GMP synthase	O4Z47_RS14865
	LPXTG cell wall anchor domain-containing protein	O4Z47_RS14875
	Bifunctional DNA primase/polymerase	O4Z47_RS14880
	PTS transporter subunit EIIC	O4Z47_RS14885
	Hypothetical protein	O4Z47_RS14910
	Hypothetical protein	O4Z47_RS14915
	VanZ family protein	O4Z47_RS14925
	IS30 family transposase	O4Z47_RS14930
	APC family permease	O4Z47_RS14935
	Recombinase family protein	O4Z47_RS14940
	Hypothetical protein	O4Z47_RS14945
	Hypothetical protein	O4Z47_RS14950
	PLDc N-terminal domain-containing protein	O4Z47_RS14960
	Type II toxin-antitoxin system YafQ family toxin	O4Z47_RS14965
	Rod shape-determining protein MreD	O4Z47_RS14980
	ABC transporter ATP-binding protein	O4Z47_RS15005
	Hypothetical protein	O4Z47_RS15055
	AraC family transcriptional regulator	O4Z47_RS15080
	Rod shape-determining protein	O4Z47_RS15090
	Hypothetical protein	O4Z47_RS15095
	Hypothetical protein	O4Z47_RS15285
	Amidohydrolase/deacetylase family metallohydrolase	O4Z47_RS15290
	DNA-directed RNA polymerase subunit beta	O4Z47_RS15295
	PAS domain-containing protein	O4Z47_RS15300
	Hypothetical protein	O4Z47_RS15305
	Multidrug effflux MFS transporter	O4Z47_RS15320
	JAB domain-containing protein	O4Z47_RS15330
	MFS transporter	O4Z47_RS15410
NCIMB 700965	Cyclopropane-fatty-acyl-phospholipid synthase family protein	CO218_RS00175
	Aldo/keto reductase	CO218_RS00180
	Collagen binding domain-containing protein	CO218_RS00215
	PTS glucose transporter subunit IIABC	CO218_RS00435
	Excinuclease ABC subunit UvrB	CO218_RS00775
	SDR family oxidoreductase	CO218_RS00910
	TetR/AcrR family transcriptional regulator	CO218_RS00915
	EamA family transporter	CO218_RS01100
	Formate C-acetyltransferase	CO218_RS01195
	Serine hydrolase domain-containing protein	CO218_RS01200
	SdpI family protein	CO218_RS01205
	Hypothetical protein	CO218_RS01305
	Type I glyceraldehyde-3-phosphate dehydrogenase	CO218_RS01310
	Hypothetical protein	CO218_RS01470
	Diaminopimelate epimerase	CO218_RS01480
	Isoleucine--tRNA ligase	CO218_RS01485

Strain	Product	Gene Locus
	DivIVA domain-containing protein	CO218_RS01490
	Metallophosphoesterase	CO218_RS01590
	RNA-binding protein	CO218_RS01615
	YggT family protein	CO218_RS01625
	TetR/AcrR family transcriptional regulator	CO218_RS01665
	Endonuclease MutS2	CO218_RS01670
	KUP/HAK/KT family potassium transporter	CO218_RS01700
	GNAT family N-acetyltransferase	CO218_RS01715
	Lipase/esterase	CO218_RS02030
	Amidohydrolase	CO218_RS03290
	Transketolase	CO218_RS03325
	DNA polymerase III subunit beta	CO218_RS03340
	ABC transporter ATP-binding protein	CO218_RS03345
	Beta-phosphoglucomutase	CO218_RS03350
	DASS family sodium-coupled anion symporter	CO218_RS03355
	Hypothetical protein	CO218_RS03435
	Hypothetical protein	CO218_RS03440
	Cell surface protein	CO218_RS03455
	Phage tail protein	CO218_RS03650
	Phage tail protein	CO218_RS03655
	Phage tail family protein	CO218_RS03660
	Sugar transporter	CO218_RS03690
	Helix-turn-helix transcriptional regulator	CO218_RS03835
	Hypothetical protein	CO218_RS03840
	Protein kinase	CO218_RS04000
	Cation diffusion facilitator family transporter	CO218_RS04330
	Amidohydrolase family protein	CO218_RS04450
	NAD(P)H-dependent glycerol-3-phosphate dehydrogenase	CO218_RS04880
	Aldehyde reductase	CO218_RS05070
	Aldo/keto reductase	CO218_RS05310
	Cell division protein SepF	CO218_RS05355
	Cell division protein FtsA	CO218_RS05370
	Cell division protein FtsQ/DivIB	CO218_RS05380
	Winged helix-turn-helix transcriptional regulator	CO218_RS05390
	UDP-N-acetylmuramoyl-L-alanine--D-glutamate ligase	CO218_RS05395
	Hypothetical protein	CO218_RS05400
	Phospho-N-acetylmuramoyl-pentapeptide- transferase	CO218_RS05405
	DUF4064 domain-containing protein	CO218_RS05415
	Aldehyde reductase	CO218_RS05425
	Penicillin-binding transpeptidase domain-containing protein	CO218_RS05430
	Cell division protein FtsL	CO218_RS05435
	Helix-turn-helix domain-containing protein	CO218_RS05600
	ABC transporter substrate-binding protein	CO218_RS05675
	Hemolysin XhlA family protein	CO218_RS05750
	XTP/dITP diphosphatase	CO218_RS05755
	ABC transporter ATP-binding protein	CO218_RS05760
	Glutamate racemase	CO218_RS05770
	YslB family protein	CO218_RS05775
	ROK family protein	CO218_RS05855
	DUF4352 domain-containing protein	CO218_RS05970
	IMP dehydrogenase	CO218_RS06010
	ABC transporter ATP-binding protein	CO218_RS06145
	Iron ABC transporter permease	CO218_RS06220
	Nitroreductase family protein	CO218_RS06235
	Mechanosensitive ion channel family protein	CO218_RS06240
	Hypothetical protein	CO218_RS06255
	Beta-galactosidase	CO218_RS06265
	N-acetylglucosamine-specific PTS transporter subunit IIBC	CO218_RS06710

Strain	Product	Gene Locus
	SHOCT domain-containing protein	CO218_RS06780
	MerR family transcriptional regulator	CO218_RS06785
	Sigma-70 family RNA polymerase sigma factor	CO218_RS07100
	Hypothetical protein	CO218_RS07130
	Iron ABC transporter permease	CO218_RS07135
	ATP-binding cassette domain-containing protein	CO218_RS07235
	YtxH domain-containing protein	CO218_RS07250
	ABC transporter permease	CO218_RS07305
	16S rRNA (cytosine(1402)-N(4))-methyltransferase RsmH	CO218_RS07365
	Endonuclease III	CO218_RS07520
	Phenolic acid decarboxylase	CO218_RS07535
	Hypothetical protein	CO218_RS07540
	LysM peptidoglycan-binding domain-containing protein	CO218_RS07735
	LysM peptidoglycan-binding domain-containing protein	CO218_RS07740
	Hypothetical protein	CO218_RS07815
	TetR family transcriptional regulator	CO218_RS08160
	HAMP domain-containing sensor histidine kinase	CO218_RS08170
	IS30 family transposase	CO218_RS08175
	Sulfite exporter TauE/SafE family protein	CO218_RS08185
	Argininosuccinate lyase	CO218_RS08740
	AzlD domain-containing protein	CO218_RS09000
	SDR family oxidoreductase	CO218_RS09120
	AzlC family ABC transporter permease	CO218_RS09355
	EpsG family protein	CO218_RS09455
	Methyltransferase	CO218_RS09460
	DUF1129 domain-containing protein	CO218_RS09515
	Universal stress protein	CO218_RS09540
	D-alanyl-D-alanine carboxypeptidase family protein	CO218_RS09590
	Sucrose-specific PTS transporter subunit IIBC	CO218_RS09625
	ABC transporter substrate-binding protein	CO218_RS09930
	SDR family oxidoreductase	CO218_RS09940
	NADPH-dependent FMN reductase	CO218_RS10015
	HD domain-containing protein	CO218_RS10845
	SGNH/GDSL hydrolase family protein	CO218_RS11060
	HNH endonuclease	CO218_RS11170
	UTP--glucose-1-phosphate uridylyltransferase GalU	CO218_RS11175
	Transposase	CO218_RS11180
	PTS glucitol/sorbitol transporter subunit IIB	CO218_RS11185
	Bifunctional lysylphosphatidylglycerol flippase/synthetase MprF	CO218_RS11190
	SDR family NAD(P)-dependent oxidoreductase	CO218_RS11195
	Division/cell wall cluster transcriptional repressor MraZ	CO218_RS11200
	DUF3397 domain-containing protein	CO218_RS11205
	Alpha-glucosidase	CO218_RS11210
	HAMP domain-containing sensor histidine kinase	CO218_RS11540
	Phage tail family protein	CO218_RS11680
	MerR family transcriptional regulator	CO218_RS11695
	MBG domain-containing protein	CO218_RS11710
	Amino acid ABC transporter ATP-binding protein	CO218_RS11745
	Hypothetical protein	CO218_RS11800
	Response regulator transcription factor	CO218_RS12355
	DUF4044 domain-containing protein	CO218_RS12515
	Helix-turn-helix domain-containing protein	CO218_RS12560
	Metal ABC transporter solute-binding protein	CO218_RS12735
	Siphovirus Gp157 family protein	CO218_RS12740
	YoaK family protein	CO218_RS12775
	DNA translocase FtsK	CO218_RS12805
	Foldase	CO218_RS13230
	WxL domain-containing protein	CO218_RS13360

Strain	Product	Gene Locus
	Anaerobic ribonucleoside-triphosphate reductase	CO218_RS13495
	YdeI/OmpD-associated family protein	CO218_RS13680
	TetR/AcrR family transcriptional regulator	CO218_RS13735
	Pyruvate oxidase	CO218_RS13740
	Hypothetical protein	CO218_RS14215
	50S ribosomal protein L5	CO218_RS14855
	BspA family leucine-rich repeat surface protein	CO218_RS14880
	TetR/AcrR family transcriptional regulator	CO218_RS15000
	CRISPR-associated endonuclease Cas2	CO218_RS15030
	Clp protease ClpP	CO218_RS15035
	Class IIb bacteriocin, lactobin A/cerein 7B family	CO218_RS15040
	DMT family transporter	CO218_RS15045
	CvfD/Ygs/GSP13 family RNA-binding post-transcriptional regulator	CO218_RS15050
	Peptidylprolyl isomerase	CO218_RS15055
	VTT domain-containing protein	CO218_RS15060
	TIGR01906 family membrane protein	CO218_RS15065
	ISL3 family transposase	CO218_RS15070
	Class 1b ribonucleoside-diphosphate reductase subunit alpha	CO218_RS15075
	Hypothetical protein	CO218_RS15080
	TIGR01457 family HAD-type hydrolase	CO218_RS15085
	Bifunctional UDP-sugar hydrolase/5-nucleotidase	CO218_RS15090
	Metallophosphoesterase family protein	CO218_RS15095
	Histidine phosphatase family protein	CO218_RS15100
	Class I SAM-dependent methyltransferase	CO218_RS15105
	Prepilin-type N-terminal cleavage/methylation domain-containing protein	CO218_RS15115
	Hypothetical protein	CO218_RS15120
	Type II secretion system protein	CO218_RS15125
	Redoxin NrdH	CO218_RS15130
	O-antigen ligase family protein	CO218_RS15135
	ECF transporter S component	CO218_RS15140
	Energy-coupling factor transporter transmembrane component T	CO218_RS15145
	Competence type IV pilus major pilin ComGC	CO218_RS15155
	Type II secretion system F family protein	CO218_RS15160
	Membrane protein	CO218_RS15165
	Competence type IV pilus ATPase ComGA	CO218_RS15170
	Ribokinase	CO218_RS15175
	Hypothetical protein	CO218_RS15180
	YebC/PmpR family DNA-binding transcriptional regulator	CO218_RS15185
	TIM barrel protein	CO218_RS15190
	KUP/HAK/KT family potassium transporter	CO218_RS15195
	Triose-phosphate isomerase	CO218_RS15200
	Translation elongation factor 4	CO218_RS15210
	LPXTG cell wall anchor domain-containing protein	CO218_RS15215
	RNA degradosome polyphosphate kinase	CO218_RS15275
	Pyruvate formate-lyase-activating protein	CO218_RS15280
	Hypothetical protein	CO218_RS15305
	PTS mannitol-specific transporter subunit IIBC	CO218_RS15310
	Hypothetical protein	CO218_RS15330
	Adenylate kinase	CO218_RS15350
	L-rhamnose isomerase	CO218_RS15355
	TRNA (cytidine(34)-2-O)-methyltransferase	CO218_RS15370
	MFS transporter	CO218_RS15375
	NAD(P)H-binding protein	CO218_RS15380
	Hypothetical protein	CO218_RS15400
	Thioredoxin	CO218_RS15405
	Cation:proton antiporter	CO218_RS15425
	N-acetyldiaminopimelate deacetylase	CO218_RS15435
	2,3,4,5-tetrahydropyridine-2,6-dicarboxylate N-acetyltransferase	CO218_RS15440

Strain	Product	Gene Locus
	Hypothetical protein	CO218_RS15450
	PBSX family phage terminase large subunit	CO218_RS15455
	Acetate kinase	CO218_RS15460
	Branched-chain amino acid transport system II carrier protein	CO218_RS15465
	Bacteriocin immunity protein	CO218_RS15470
	DUF2089 family protein	CO218_RS15480
	Holin	CO218_RS15485
	Hypothetical protein	CO218_RS15490
	Histidine kinase	CO218_RS15510
	Hypothetical protein	CO218_RS15515
	Asparagine synthase (glutamine-hydrolyzing)	CO218_RS15540
	DUF948 domain-containing protein	CO218_RS15545
	Methyltransferase domain-containing protein	CO218_RS15550
	DsbA family oxidoreductase	CO218_RS15555
	5-oxoprolinase subunit PxpB	CO218_RS15560
	AAA family ATPase	CO218_RS15565
	Hypothetical protein	CO218_RS15570
	C69 family dipeptidase	CO218_RS15575
	Translation initiation factor IF-1	CO218_RS15580
	Hypothetical protein	CO218_RS15595
	Methionine--tRNA ligase	CO218_RS15620
	Catabolite control protein A	CO218_RS15635
	Xaa-Pro peptidase family protein	CO218_RS15640
	GRP family sugar transporter	CO218_RS15645
	ATP-binding protein	CO218_RS15675
	Hypothetical protein	CO218_RS15685
	Divalent metal cation transporter	CO218_RS15690
	DMT family transporter	CO218_RS15695
	Helix-turn-helix domain-containing protein	CO218_RS15705
	Hypothetical protein	CO218_RS15710
	ABC transporter ATP-binding protein	CO218_RS15730
	Biotin-dependent carboxyltransferase	CO218_RS15735
	Cation:proton antiporter	CO218_RS15775
	LicD family protein	CO218_RS15860
	DNA-3-methyladenine glycosylase I	CO218_RS15870
	Helix-turn-helix domain-containing protein	CO218_RS15875
	N-acetyltransferase GCN5	CO218_RS15885
	AAA family ATPase	CO218_RS15890
	Hypothetical protein	CO218_RS15910
	UbiX family flavin prenyltransferase	CO218_RS15920
	MerR family transcriptional regulator	CO218_RS15925
	PTS cellobiose transporter subunit IIC	CO218_RS15930
	FAD synthetase family protein	CO218_RS15935
	Class I SAM-dependent methyltransferase	CO218_RS15940
	Fur family transcriptional regulator	CO218_RS15950
	TRNA adenosine(34) deaminase TadA	CO218_RS15955
	FAD-dependent oxidoreductase	CO218_RS15960
	TIGR00730 family Rossman fold protein	CO218_RS15975
	MucBP domain-containing protein	CO218_RS15980
	AraC family transcriptional regulator	CO218_RS15985
	SDR family oxidoreductase	CO218_RS16000
	WxL domain-containing protein	CO218_RS16005
	PTS ascorbate transporter subunit IIC	CO218_RS16040
	PTS sugar transporter subunit IIA	CO218_RS16045
	Phage scaffolding protein	CO218_RS16065
	30S ribosomal protein S14	CO218_RS16085
	Alpha/beta hydrolase	CO218_RS16090
	Nicotinamide riboside transporter PnuC	CO218_RS16100

Strain	Product	Gene Locus
	AI-2E family transporter	CO218_RS16125
	Lactonase family protein	CO218_RS16150
	Hypothetical protein	CO218_RS16155
	Copper homeostasis protein CutC	CO218_RS16160
	SpaA isopeptide-forming pilin-related protein	CO218_RS16165
	RluA family pseudouridine synthase	CO218_RS16175
	NAD kinase	CO218_RS16185
	GTP pyrophosphokinase family protein	CO218_RS16225
	DsbA family protein	CO218_RS16235
	Class 1b ribonucleoside-diphosphate reductase subunit beta	CO218_RS16250
	Competence protein CoiA family protein	CO218_RS16280
	Adaptor protein MecA	CO218_RS16285
	Transcriptional regulator SpxA	CO218_RS16290
Q7	Peptide deformylase	B1H25_RS00100
	Hypothetical protein	B1H25_RS00105
	NAD(P)/FAD-dependent oxidoreductase	B1H25_RS00140
	Minor capsid protein	B1H25_RS00235
	MIP/aquaporin family protein	B1H25_RS00240
	Bacteriocin immunity protein	B1H25_RS00490
	Glycoside hydrolase family 125 protein	B1H25_RS00645
	Adenylosuccinate lyase	B1H25_RS00855
	Adenylosuccinate synthase	B1H25_RS01810
	WxL domain-containing protein	B1H25_RS02460
	Hypothetical protein	B1H25_RS02530
	Hypothetical protein	B1H25_RS02790
	Tyrosine-protein phosphatase	B1H25_RS03575
	Cardiolipin synthase	B1H25_RS03985
	Glycyl-radical enzyme activating protein	B1H25_RS04050
	Fructose-6-phosphate aldolase	B1H25_RS04055
	DNA-directed RNA polymerase subunit epsilon	B1H25_RS04075
	Ribonuclease J	B1H25_RS04080
	Hypothetical protein	B1H25_RS04085
	TRNA 2-thiouridine(34) synthase MnmA	B1H25_RS04090
	M57 family metalloprotease	B1H25_RS04095
	Diacylglycerol kinase family lipid kinase	B1H25_RS04100
	Aspartate kinase	B1H25_RS04105
	Hypothetical protein	B1H25_RS04110
	SH3 domain-containing protein	B1H25_RS04250
	Restriction endonuclease subunit S	B1H25_RS04360
	Hypothetical protein	B1H25_RS04365
	Hypothetical protein	B1H25_RS04370
	HAD-IC family P-type ATPase	B1H25_RS04375
	Hypothetical protein	B1H25_RS04395
	Sugar-binding transcriptional regulator	B1H25_RS04410
	C40 family peptidase	B1H25_RS04415
	Tyrosine-type recombinase/integrase	B1H25_RS04420
	DNA/RNA non-specific endonuclease	B1H25_RS04425
	DUF1831 domain-containing protein	B1H25_RS04430
	Hypothetical protein	B1H25_RS04490
	Hypothetical protein	B1H25_RS04495
	Hypothetical protein	B1H25_RS04510
	Aldo/keto reductase	B1H25_RS04520
	DegV family protein	B1H25_RS04530
	50S ribosomal protein L7/L12	B1H25_RS04545
	Ferrous iron transport protein B	B1H25_RS04550
	Hypothetical protein	B1H25_RS04555
	Hypothetical protein	B1H25_RS04560
	Hypothetical protein	B1H25_RS04565

Strain	Product	Gene Locus
	Hypothetical protein	B1H25_RS04570
	Hypothetical protein	B1H25_RS04575
	Hypothetical protein	B1H25_RS04580
	Hypothetical protein	B1H25_RS04585
	Hypothetical protein	B1H25_RS04590
	Ribose-phosphate diphosphokinase	B1H25_RS04610
	ABC transporter permease	B1H25_RS04615
	Tetratricopeptide repeat protein	B1H25_RS04620
	Histidine phosphatase family protein	B1H25_RS04625
	5-methylthioadenosine/adenosylhomocysteine nucleosidase	B1H25_RS04630
	Hypothetical protein	B1H25_RS04650
	DUF3037 domain-containing protein	B1H25_RS06145
	DUF262 domain-containing protein	B1H25_RS06160
	WxL domain-containing protein	B1H25_RS06420
	Amino acid permease	B1H25_RS07160
	SH3 domain-containing protein	B1H25_RS07170
	DUF1905 domain-containing protein	B1H25_RS07185
	Helix-turn-helix domain-containing protein	B1H25_RS09330
	Cof-type HAD-IIB family hydrolase	B1H25_RS09380
	Hypothetical protein	B1H25_RS09385
	MFS transporter	B1H25_RS09390
	Hypothetical protein	B1H25_RS10010
	MFS transporter	B1H25_RS10715
	HK97 gp10 family phage protein	B1H25_RS10720
	SDR family oxidoreductase	B1H25_RS12315
	Glycoside hydrolase family 1 protein	B1H25_RS12630
	Subtype B tannase	B1H25_RS13090
	Hypothetical protein	B1H25_RS13125
	Hypothetical protein	B1H25_RS13130
	DUF916 and DUF3324 domain-containing protein	B1H25_RS13135
	PTS sugar transporter subunit IIC	B1H25_RS13985
	Alpha/beta hydrolase	B1H25_RS14325
	50S ribosomal protein L24	B1H25_RS14330
	Minor capsid protein	B1H25_RS14340
	WxL domain-containing protein	B1H25_RS14345
	Thiamine pyrophosphate-binding protein	B1H25_RS14350
	DeoR/GlpR transcriptional regulator	B1H25_RS14355
	Hsp20/alpha crystallin family protein	B1H25_RS14360
	DUF916 and DUF3324 domain-containing protein	B1H25_RS14380
	WxL domain-containing protein	B1H25_RS14385
	MFS transporter	B1H25_RS14390
	XkdX family protein	B1H25_RS14395
	TrkA family potassium uptake protein	B1H25_RS14415
	Potassium transporter TrkG	B1H25_RS14490
	NUDIX hydrolase	B1H25_RS14500
	Ammonium transporter	B1H25_RS14505
B21	Amino acid permease	SH83_RS01670
	XkdX family protein	SH83_RS01695
	Helix-turn-helix transcriptional regulator	SH83_RS01865
	Translational GTPase TypA	SH83_RS02530
	DUF6198 family protein	SH83_RS02560
	Hypothetical protein	SH83_RS02565
	Hypothetical protein	SH83_RS02570
	Hypothetical protein	SH83_RS02575
	Glycosyltransferase	SH83_RS02580
	MFS transporter	SH83_RS02590
	Preprotein translocase subunit SecE	SH83_RS03780
	Hypothetical protein	SH83_RS03785

Strain	Product	Gene Locus
	Phage head-tail connector protein	SH83_RS03790
	GTPase ObgE	SH83_RS03795
	Hypothetical protein	SH83_RS03800
	Threonine/serine exporter family protein	SH83_RS03805
	DeoR/GlpR family DNA-binding transcription regulator	SH83_RS03810
	1-phosphofructokinase	SH83_RS03815
	Inositol monophosphatase family protein	SH83_RS03825
	Fructose-specific PTS transporter subunit EIIC	SH83_RS03830
	MarR family winged helix-turn-helix transcriptional regulator	SH83_RS03835
	Metallophosphoesterase	SH83_RS03840
	Hypothetical protein	SH83_RS03845
	Oligosaccharide flippase family protein	SH83_RS03850
	Hypothetical protein	SH83_RS03855
	Hypothetical protein	SH83_RS03860
	MucBP domain-containing protein	SH83_RS03865
	LysM peptidoglycan-binding domain-containing protein	SH83_RS04000
	LysM domain-containing protein	SH83_RS04005
	Hypothetical protein	SH83_RS04765
	30S ribosomal protein S9	SH83_RS04770
	RluA family pseudouridine synthase	SH83_RS05020
	Hypothetical protein	SH83_RS05025
	Hypothetical protein	SH83_RS05030
	IS30 family transposase	SH83_RS05035
	Hypothetical protein	SH83_RS05040
	Type IV secretory system conjugative DNA transfer family protein	SH83_RS05045
	Hypothetical protein	SH83_RS05050
	Sn-glycerol-3-phosphate ABC transporter ATP-binding protein UgpC	SH83_RS05055
	Universal stress protein	SH83_RS05060
	IS30-like element ISLsa1 family transposase	SH83_RS05065
	Hypothetical protein	SH83_RS05070
	Hypothetical protein	SH83_RS05075
	Thioredoxin	SH83_RS05085
	Hypothetical protein	SH83_RS05120
	Glycosyltransferase	SH83_RS05125
	Hypothetical protein	SH83_RS05130
	P27 family phage terminase small subunit	SH83_RS05135
	IS3 family transposase	SH83_RS05140
	Alpha-glucosidase	SH83_RS05145
	Sucrose-6-phosphate hydrolase	SH83_RS05150
	CTP synthase	SH83_RS05160
	Thioredoxin-disulfide reductase	SH83_RS05165
	50S ribosomal protein L5	SH83_RS07215
	MucBP domain-containing protein	SH83_RS07240
	Transcription termination/antitermination protein NusG	SH83_RS08770
	Hypothetical protein	SH83_RS08845
	Glycosyltransferase family 4 protein	SH83_RS08920
	Glycerol-3-phosphate cytidylyltransferase	SH83_RS08965
	Helix-turn-helix domain-containing protein	SH83_RS08970
	Helix-turn-helix domain-containing protein	SH83_RS08975
	Sugar transferase	SH83_RS08995
	SDR family NAD(P)-dependent oxidoreductase	SH83_RS09005
	Universal stress protein	SH83_RS09015
	Hypothetical protein	SH83_RS09025
	Tyrosine protein phosphatase	SH83_RS09030
	CpsD/CapB family tyrosine-protein kinase	SH83_RS09035
	MucBP domain-containing protein	SH83_RS10720
	Excinuclease ABC subunit UvrC	SH83_RS10725
	Amino acid ABC transporter ATP-binding protein	SH83_RS10730

Strain	Product	Gene Locus
	Dihydrolipoyl dehydrogenase	SH83_RS10735
	Alpha/beta hydrolase	SH83_RS10745
	YlbG family protein	SH83_RS10765
	GntR family transcriptional regulator	SH83_RS10770
	Hypothetical protein	SH83_RS10790
	Helix-turn-helix transcriptional regulator	SH83_RS10795
	Hypothetical protein	SH83_RS10840
	Hypothetical protein	SH83_RS10845
	Universal stress protein	SH83_RS10850
	Putative glycosyltransferase, exosortase G system-associated	SH83_RS10865
	Type II CRISPR RNA-guided endonuclease Cas9	SH83_RS10870
	Helix-turn-helix transcriptional regulator	SH83_RS13555
	Nucleoside triphosphate pyrophosphohydrolase family protein	SH83_RS13560
	Amino acid permease	SH83_RS13565
	50S ribosomal protein L1	SH83_RS13570
	PTS sugar transporter subunit IIA	SH83_RS13575
	FtsW/RodA/SpoVE family cell cycle protein	SH83_RS13580
	Ribosome biogenesis GTP-binding protein YihA/YsxC	SH83_RS13585
	UPF0223 family protein	SH83_RS13590
	Folate family ECF transporter S component	SH83_RS13595
	Hypothetical protein	SH83_RS13600
	LytTR family DNA-binding domain-containing protein	SH83_RS13605
	VanZ family protein	SH83_RS13610
	C69 family dipeptidase	SH83_RS13615
	Dyp-type peroxidase	SH83_RS13620
	Hypothetical protein	SH83_RS13625
	Hypothetical protein	SH83_RS13630
	Lactate dehydrogenase	SH83_RS13635
	Potassium-transporting ATPase subunit KdpA	SH83_RS13640
	ATP-dependent Clp protease ATP-binding subunit ClpX	SH83_RS13645
	Trigger factor	SH83_RS13650
	Elongation factor Tu	SH83_RS13655
	DUF2929 family protein	SH83_RS14645
	50S ribosomal protein L17	SH83_RS14650
	DUF6287 domain-containing protein	SH83_RS14655
	Hypothetical protein	SH83_RS14660
	Type II toxin-antitoxin system RelE/ParE family toxin	SH83_RS15815
	Hypothetical protein	SH83_RS15820
	Hypothetical protein	SH83_RS15830
	SH3 domain-containing protein	SH83_RS15835
	Threonine/serine exporter family protein	SH83_RS15840
	Ribonuclease J	SH83_RS15845
	Hypothetical protein	SH83_RS15850
	4-hydroxy-tetrahydrodipicolinate synthase	SH83_RS15855
	MFS transporter	SH83_RS15860
	Hypothetical protein	SH83_RS15865
	50S ribosomal protein L11	SH83_RS15870
	30S ribosomal protein S20	SH83_RS15875
	Multicopper oxidase domain-containing protein	SH83_RS15880
	DNA polymerase III subunit delta	SH83_RS15885
	LysR family transcriptional regulator	SH83_RS15890
	Type II CAAX endopeptidase family protein	SH83_RS15895
	Helix-turn-helix transcriptional regulator	SH83_RS15900
	Hypothetical protein	SH83_RS15905
	UbiX family flavin prenyltransferase	SH83_RS15915
	Energy-coupling factor transporter transmembrane component T	SH83_RS15920
	NUDIX hydrolase	SH83_RS15925
	DNA internalization-related competence protein ComEC/Rec2	SH83_RS15930

Strain	Product	Gene Locus
	ComE operon protein 2	SH83_RS15935
	Helix-hairpin-helix domain-containing protein	SH83_RS15940
	Hypothetical protein	SH83_RS15945
	SepM family pheromone-processing serine protease	SH83_RS16030
	Pantetheine-phosphate adenylyltransferase	SH83_RS16035
	Class I SAM-dependent methyltransferase	SH83_RS16045
	16S rRNA (guanine(966)-N(2))-methyltransferase RsmD	SH83_RS16125
KACC 92189	MFS transporter	COO33_RS00775
	MupG family TIM beta-alpha barrel fold protein	COO33_RS00970
	Hypothetical protein	COO33_RS00975
	DNA topoisomerase (ATP-hydrolyzing) subunit B	COO33_RS01640
	MFS transporter	COO33_RS01690
	Hypothetical protein	COO33_RS01695
	Hypothetical protein	COO33_RS01740
	D-alanyl-lipoteichoic acid biosynthesis protein DltD	COO33_RS01760
	AEC family transporter	COO33_RS01775
	ABC transporter permease	COO33_RS01810
	D-alanine--poly(phosphoribitol) ligase subunit DltC	COO33_RS01815
	Transcriptional repressor	COO33_RS01820
	SH3 domain-containing protein	COO33_RS02525
	Peptide chain release factor 1	COO33_RS02540
	Cation-translocating P-type ATPase	COO33_RS02570
	Hypothetical protein	COO33_RS02575
	S-ribosylhomocysteine lyase	COO33_RS02690
	CPBP family intramembrane metalloprotease	COO33_RS02705
	Proline-specific peptidase family protein	COO33_RS02715
	Hypothetical protein	COO33_RS02725
	D-alanyl-lipoteichoic acid biosynthesis protein DltB	COO33_RS02730
	Teichoic acid D-Ala incorporation-associated protein DltX	COO33_RS02735
	Serine hydrolase domain-containing protein	COO33_RS02740
	Molecular chaperone DnaJ	COO33_RS02750
	Class 1b ribonucleoside-diphosphate reductase subunit beta	COO33_RS03515
	Hypothetical protein	COO33_RS03520
	Hypothetical protein	COO33_RS03525
	Hypothetical protein	COO33_RS03530
	Hypothetical protein	COO33_RS03535
	Hypothetical protein	COO33_RS03540
	DHA2 family efflux MFS transporter permease subunit	COO33_RS03545
	Hypothetical protein	COO33_RS03680
	Phospho-sugar mutase	COO33_RS03690
	Phage holin family protein	COO33_RS05180
	Replication-associated recombination protein A	COO33_RS05550
	Hypothetical protein	COO33_RS05680
	Peptide ABC transporter substrate-binding protein	COO33_RS05685
	XkdX family protein	COO33_RS05810
	Endodeoxyribonuclease	COO33_RS05830
	DNA replication initiation control protein YabA	COO33_RS05835
	Helix-turn-helix transcriptional regulator	COO33_RS06895
	Molecular chaperone DnaK	COO33_RS06900
	Membrane protein insertase YidC	COO33_RS06920
	Phage gp6-like head-tail connector protein	COO33_RS07300
	Glycosyltransferase family 2 protein	COO33_RS07320
	GtrA family protein	COO33_RS07325
	Nucleotide exchange factor GrpE	COO33_RS07330
	Heat-inducible transcriptional repressor HrcA	COO33_RS07345
	XRE family transcriptional regulator	COO33_RS07350
	MarR family winged helix-turn-helix transcriptional regulator	COO33_RS07400
	Phage terminase small subunit P27 family	COO33_RS07405

Strain	Product	Gene Locus
	Single-stranded DNA-binding protein	COO33_RS07410
	Glutamate-5-semialdehyde dehydrogenase	COO33_RS07420
	Beta-phosphoglucomutase	COO33_RS07425
	Hypothetical protein	COO33_RS07430
	Hypothetical protein	COO33_RS07435
	TetM/TetW/TetO/TetS family tetracycline resistance ribosomal protection protein	COO33_RS07440
	Phage tail protein	COO33_RS07445
	Two-peptide bacteriocin plantaricin EF subunit PlnE	COO33_RS07455
	Hypothetical protein	COO33_RS07460
	Type B 50S ribosomal protein L31	COO33_RS07465
	DUF72 domain-containing protein	COO33_RS07475
	SH3 domain-containing protein	COO33_RS07490
	Helix-turn-helix transcriptional regulator	COO33_RS07930
	DUTP diphosphatase	COO33_RS07935
	PRD domain-containing protein	COO33_RS07940
	Glycosyltransferase	COO33_RS07945
	Peptide ABC transporter substrate-binding protein	COO33_RS07950
	L-ribulose-5-phosphate 4-epimerase	COO33_RS07955
	Acetolactate decarboxylase	COO33_RS07960
	FGGY-family carbohydrate kinase	COO33_RS08360
	MFS transporter	COO33_RS08365
	Hypothetical protein	COO33_RS08375
	DEAD/DEAH box helicase family protein	COO33_RS08380
	ABC transporter permease	COO33_RS08385
	Hypothetical protein	COO33_RS08390
	Hypothetical protein	COO33_RS08395
	Hypothetical protein	COO33_RS09500
	Hypothetical protein	COO33_RS09805
	Hypothetical protein	COO33_RS09865
	Hypothetical protein	COO33_RS09870
	Hypothetical protein	COO33_RS10590
	Hypothetical protein	COO33_RS10595
	Hypothetical protein	COO33_RS10600
	YslB family protein	COO33_RS10605
	Glutamate racemase	COO33_RS10610
	XTP/dITP diphosphatase	COO33_RS10615
	Riboflavin biosynthesis protein RibF	COO33_RS11080
	Hypothetical protein	COO33_RS11150
	Phage portal protein	COO33_RS12530
	ImmA/IrrE family metallo-endopeptidase	COO33_RS12535
	GntR family transcriptional regulator	COO33_RS12550
	Phage tail family protein	COO33_RS12555
	Phage tail family protein	COO33_RS12560
	Peptide cleavage/export ABC transporter	COO33_RS12565
	Bacterial Ig-like domain-containing protein	COO33_RS12575
	Phage tail protein	COO33_RS12585
	Phage tail spike protein	COO33_RS12590
	Phage tail tape measure protein	COO33_RS12600
	Phage tail tape measure protein	COO33_RS12605
	Phage tail tape measure protein	COO33_RS12610
	Shikimate kinase	COO33_RS12615
	PTS sugar transporter subunit IIC	COO33_RS14145
	Glycosyltransferase	COO33_RS15620
	Hypothetical protein	COO33_RS15625
	Prephenate dehydrogenase	COO33_RS15630
	Oligosaccharide flippase family protein	COO33_RS15635
	HNH endonuclease	COO33_RS15640

Strain	Product	Gene Locus
	3-phosphoshikimate 1-carboxyvinyltransferase	COO33_RS15650
	Phage tail tape measure protein	COO33_RS15655
	Hypothetical protein	COO33_RS15660
	Transcription termination factor Rho	COO33_RS15665
	Hypothetical protein	COO33_RS15670
	Hypothetical protein	COO33_RS15675
	Hypothetical protein	COO33_RS15680
	MIP/aquaporin family protein	COO33_RS15685
	Chorismate synthase	COO33_RS15695
	LysR family transcriptional regulator	COO33_RS15705
	NAD(P)H-binding protein	COO33_RS15725
	PTS sugar transporter subunit IIB	COO33_RS15740
	MFS transporter	COO33_RS15835
	30S ribosome-binding factor RbfA	COO33_RS15840
	DUF4428 domain-containing protein	COO33_RS15850
	Sugar phosphate isomerase/epimerase	COO33_RS15860
	PTS fructose-like transporter subunit IIBC	COO33_RS15865
	Class II fructose-1,6-bisphosphate aldolase	COO33_RS15870
	DNA repair protein RadA	COO33_RS15900
	Fructose-6-phosphate aldolase	COO33_RS15925
	Translation initiation factor IF-2	COO33_RS15930
	Bifunctional hydroxymethylpyrimidine kinase/phosphomethylpyrimidine kinase	COO33_RS16005
	MFS transporter	COO33_RS16010
	NAD-dependent succinate-semialdehyde dehydrogenase	COO33_RS16020
	Ribosomal L7Ae/L30e/S12e/Gadd45 family protein	COO33_RS16025
	MarR family transcriptional regulator	COO33_RS16030
	Hypothetical protein	COO33_RS16035
	Hypothetical protein	COO33_RS16050
	NCS2 family permease	COO33_RS16055
	Transcription termination factor NusA	COO33_RS16060
	ABC-F family ATP-binding cassette domain-containing protein	COO33_RS16085
	Xaa-Pro dipeptidyl-peptidase	COO33_RS16110
	Ribosome maturation factor RimP	COO33_RS16125
	Hypothetical protein	COO33_RS16130
	Metalloregulator ArsR/SmtB family transcription factor	COO33_RS16135
	Proline--tRNA ligase	COO33_RS16140
	Hypothetical protein	COO33_RS16145
	Phosphatidate cytidylyltransferase	COO33_RS16150
	Isoprenyl transferase	COO33_RS16155
	PIN/TRAM domain-containing protein	COO33_RS16160
	ISL3 family transposase	COO33_RS16165
	MFS transporter	COO33_RS16170
	Hypothetical protein	COO33_RS16190
	Ribosome recycling factor	COO33_RS16195
	UMP kinase	COO33_RS16200
	Transketolase	COO33_RS16205
	Cytosine permease	COO33_RS16255
	DNA-directed RNA polymerase subunit alpha	COO33_RS16260
	DNA cytosine methyltransferase	COO33_RS16270
	DNA topoisomerase	COO33_RS16275
	DNA/RNA non-specific endonuclease	COO33_RS16280
	DUF1351 domain-containing protein	COO33_RS16285
	DUF1617 family protein	COO33_RS16295
	DUF1792 domain-containing protein	COO33_RS16300
	DUF1972 domain-containing protein	COO33_RS16305
	Acetyl-CoA carboxylase biotin carboxyl carrier protein subunit	COO33_RS16310
	Acetyl-CoA carboxylase biotin carboxylase subunit	COO33_RS16315

Strain	Product	Gene Locus
	Acyltransferase	COO33_RS16320
	Acyltransferase	COO33_RS16325
	Acyltransferase	COO33_RS16330
	Alpha/beta fold hydrolase	COO33_RS16335
	Amidohydrolase family protein	COO33_RS16340
	Amino acid permease	COO33_RS16345
	Amino acid permease	COO33_RS16350
	AraC family transcriptional regulator	COO33_RS16355
	Hypothetical protein	COO33_RS16365
	Translation elongation factor Ts	COO33_RS16400
	Hypothetical protein	COO33_RS16415
	30S ribosomal protein S2	COO33_RS16420
	Ribose-phosphate diphosphokinase	COO33_RS16425
	Triphosphoribosyl-dephospho-CoA synthase	COO33_RS16430
	D-2-hydroxyacid dehydrogenase	COO33_RS16435
	GIY-YIG nuclease family protein	COO33_RS16440
	Restriction endonuclease subunit S	COO33_RS16445
	ATP-binding cassette domain-containing protein	COO33_RS16450
	TRNA1(Val) (adenine(37)-N6)-methyltransferase	COO33_RS16460
	1-acyl-sn-glycerol-3-phosphate acyltransferase	COO33_RS16465
	DUF4811 domain-containing protein	COO33_RS16470
	Minor capsid protein	COO33_RS16475
	YneF family protein	COO33_RS16480
	50S ribosomal protein L4	COO33_RS16485
	Hypothetical protein	COO33_RS16495
	Hypothetical protein	COO33_RS16500
	DUF896 domain-containing protein	COO33_RS16505
	Hypothetical protein	COO33_RS16510
	Thiamine phosphate synthase	COO33_RS16515
	MFS transporter	COO33_RS16525
	Transcriptional repressor LexA	COO33_RS16560
	Shikimate dehydrogenase	COO33_RS16565
	SDR family oxidoreductase	COO33_RS16570
	AAA family ATPase	COO33_RS16575
	Ribosomal-processing cysteine protease Prp	COO33_RS16580
	N-acetyltransferase GCN5	COO33_RS16595
	Hypothetical protein	COO33_RS16635
	Hydroxymethylglutaryl-CoA synthase	COO33_RS16650
	MobA/MobL family protein	COO33_RS16655
	HigA family addiction module antitoxin	COO33_RS16660
	Hypothetical protein	COO33_RS16700
	MDR family MFS transporter	COO33_RS16710
	HD domain-containing protein	COO33_RS16725
	D-2-hydroxyacid dehydrogenase	COO33_RS16755
	50S ribosomal protein L30	COO33_RS16760
	Hypothetical protein	COO33_RS16765
	Phosphoketolase family protein	COO33_RS16805
	Hypothetical protein	COO33_RS16810
	Glycosyltransferase family 2 protein	COO33_RS16815
	Hypothetical protein	COO33_RS16820
	Cysteine--tRNA ligase	COO33_RS16825
	Peptide ABC transporter substrate-binding protein	COO33_RS16830
	Glycosyl hydrolase family 65 protein	COO33_RS16835
	Hypothetical protein	COO33_RS16840
	LacI family DNA-binding transcriptional regulator	COO33_RS16845
	BglG family transcription antiterminator	COO33_RS16850
	ABC transporter ATP-binding protein	COO33_RS16855
	Hemolysin XhlA family protein	COO33_RS16860

Strain	Product	Gene Locus
	LysM peptidoglycan-binding domain-containing protein	COO33_RS16865
	DNA replication/repair protein RecF	COO33_RS16875
	1,4-alpha-glucan branching protein GlgB	COO33_RS16880
	Pyridoxamine 5-phosphate oxidase family protein	COO33_RS16885
	Hypothetical protein	COO33_RS16890
	Helix-turn-helix domain-containing protein	COO33_RS16895
	Cell wall hydrolase/muramidase	COO33_RS16900
	Mini-ribonuclease 3	COO33_RS16905
	23S rRNA (guanosine(2251)-2-O)-methyltransferase RlmB	COO33_RS16910
	Clp protease ClpP	COO33_RS16945
	Transcriptional regulator	COO33_RS16960
	Heavy metal-binding domain-containing protein	COO33_RS16970
	NYN domain-containing protein	COO33_RS16980
	Helix-turn-helix transcriptional regulator	COO33_RS16985
	50S ribosomal protein L33	COO33_RS16990
	Hypothetical protein	COO33_RS17030
	Hypothetical protein	COO33_RS17045
	Hypothetical protein	COO33_RS17050
KM2	SMI1/KNR4 family protein	JQC82_RS01020
	Class 1b ribonucleoside-diphosphate reductase subunit alpha	JQC82_RS01640
	ATP-binding cassette domain-containing protein	JQC82_RS01645
	Aspartate-semialdehyde dehydrogenase	JQC82_RS01660
	Hypothetical protein	JQC82_RS01720
	Gamma-glutamyl-gamma-aminobutyrate hydrolase family protein	JQC82_RS03595
	Oleate hydratase	JQC82_RS03600
	Hypothetical protein	JQC82_RS03605
	AAA family ATPase	JQC82_RS03775
	Alpha/beta fold hydrolase	JQC82_RS04465
	ABC transporter permease	JQC82_RS04750
	ABC transporter permease	JQC82_RS04755
	ABC transporter permease	JQC82_RS04760
	Hypothetical protein	JQC82_RS04765
	Hypothetical protein	JQC82_RS04770
	Major capsid protein	JQC82_RS04775
	MerR family transcriptional regulator	JQC82_RS04780
	Hypothetical protein	JQC82_RS04790
	Hypothetical protein	JQC82_RS04795
	Hypothetical protein	JQC82_RS04800
	GntR family transcriptional regulator	JQC82_RS04805
	Amino acid ABC transporter ATP-binding protein	JQC82_RS04815
	Hypothetical protein	JQC82_RS04820
	Glycine cleavage system protein H	JQC82_RS04850
	Acyltransferase family protein	JQC82_RS04855
	Hypothetical protein	JQC82_RS04860
	RNA polymerase sigma factor RpoD	JQC82_RS04870
	Hypothetical protein	JQC82_RS04875
	Hypothetical protein	JQC82_RS04880
	Hypothetical protein	JQC82_RS04890
	Sugar O-acetyltransferase	JQC82_RS04910
	HPr(Ser) kinase/phosphatase	JQC82_RS04925
	Methyltransferase domain-containing protein	JQC82_RS05635
	Matrixin family metalloprotease	JQC82_RS05640
	Glycine--tRNA ligase subunit beta	JQC82_RS05645
	Carboxymuconolactone decarboxylase family protein	JQC82_RS05675
	Recombinase RecT	JQC82_RS05695
	50S ribosomal protein L11 methyltransferase	JQC82_RS05730
	Transposase	JQC82_RS05740
	Glycine--tRNA ligase subunit alpha	JQC82_RS05760

Strain	Product	Gene Locus
	Minor capsid protein	JQC82_RS05765
	Transposase	JQC82_RS05870
	Nucleoside hydrolase	JQC82_RS05875
	DNA-3-methyladenine glycosylase	JQC82_RS08905
	Glutamate 5-kinase	JQC82_RS08910
	IS3 family transposase	JQC82_RS08915
	Metallophosphoesterase	JQC82_RS08935
	Ankyrin repeat domain-containing protein	JQC82_RS08940
	DNA repair protein RecO	JQC82_RS08965
	Citrate lyase acyl carrier protein	JQC82_RS08970
	GTPase Era	JQC82_RS08975
	Hypothetical protein	JQC82_RS08980
	DUF536 domain-containing protein	JQC82_RS09000
	DUF853 family protein	JQC82_RS09020
	DUF927 domain-containing protein	JQC82_RS09025
	RRNA maturation RNase YbeY	JQC82_RS09030
	PhoH family protein	JQC82_RS09035
	Aldo/keto reductase	JQC82_RS09050
	Aldo/keto reductase	JQC82_RS09060
	16S rRNA (uracil(1498)-N(3))-methyltransferase	JQC82_RS09065
	LCP family protein	JQC82_RS09075
	GatB/YqeY domain-containing protein	JQC82_RS10520
	30S ribosomal protein S21	JQC82_RS10525
	LBP_cg2779 family protein	JQC82_RS10530
	C1 family peptidase	JQC82_RS10690
	Ribonuclease H family protein	JQC82_RS10695
	Hypothetical protein	JQC82_RS10765
	Phosphoribosyltransferase family protein	JQC82_RS10790
	16S rRNA (adenine(1518)-N(6)/adenine(1519)-N(6))- dimethyltransferase RsmA	JQC82_RS10795
	Hypothetical protein	JQC82_RS10820
	Lipoprotein	JQC82_RS11820
	GntR family transcriptional regulator	JQC82_RS11910
	Peptide deformylase	JQC82_RS12005
	Pyruvate, water dikinase regulatory protein	JQC82_RS13195
	Hypothetical protein	JQC82_RS13200
	Hypothetical protein	JQC82_RS13205
	FAD-linked oxidase C-terminal domain-containing protein	JQC82_RS13210
	PTS ascorbate transporter subunit IIC	JQC82_RS13215
	Acyltransferase	JQC82_RS13230
	TRNA-dihydrouridine synthase family protein	JQC82_RS13440
	Hypothetical protein	JQC82_RS13660
	Helix-turn-helix transcriptional regulator	JQC82_RS13665
	Hypothetical protein	JQC82_RS13670
	Hypothetical protein	JQC82_RS13675
	RNA-binding cell elongation regulator Jag/EloR	JQC82_RS13680
	Glycosyltransferase	JQC82_RS13760
	GtrA family protein	JQC82_RS13765
	LysR family transcriptional regulator	JQC82_RS13770
	Kinase	JQC82_RS13775
	Subtype B tannase	JQC82_RS13875
	ABC transporter permease	JQC82_RS14655
	Spx/MgsR family RNA polymerase-binding regulatory protein	JQC82_RS14660
	DUF669 domain-containing protein	JQC82_RS14665
	Sugar ABC transporter permease	JQC82_RS14670
	Sugar ABC transporter permease	JQC82_RS14675
	HIRAN domain-containing protein	JQC82_RS14680
	UDP-glucose--hexose-1-phosphate uridylyltransferase	JQC82_RS15270

Strain	Product	Gene Locus
	Anaerobic ribonucleoside-triphosphate reductase	JQC82_RS15280
	Shikimate dehydrogenase	JQC82_RS15285
	NUDIX domain-containing protein	JQC82_RS15290
	Biotin--[acetyl-CoA-carboxylase] ligase	JQC82_RS15295
	Hypothetical protein	JQC82_RS15310
	Hypothetical protein	JQC82_RS15315
	PTS sugar transporter subunit IIA	JQC82_RS15345
	Choloylglycine hydrolase	JQC82_RS15350
	SDR family oxidoreductase	JQC82_RS15360
	YitT family protein	JQC82_RS15365
	Hypothetical protein	JQC82_RS15370
	Peptide-methionine (S)-S-oxide reductase MsrA	JQC82_RS15375
	ABC transporter permease	JQC82_RS15380
	Aspartate--tRNA ligase	JQC82_RS15385
	Sugar-phosphatase	JQC82_RS15390
	Helix-turn-helix domain-containing protein	JQC82_RS15395
	Helix-turn-helix transcriptional regulator	JQC82_RS15410
	Hypothetical protein	JQC82_RS15435
	Hypothetical protein	JQC82_RS15440
	Histidine--tRNA ligase	JQC82_RS15445
	Virulence protein	JQC82_RS15455
	Hypothetical protein	JQC82_RS15460
	FAD-dependent oxidoreductase	JQC82_RS15470
	Response regulator transcription factor	JQC82_RS15480
	Hypothetical protein	JQC82_RS15550
	IS256 family transposase	JQC82_RS15570
	CvpA family protein	JQC82_RS15595
	Phosphate acetyltransferase	JQC82_RS15690
	Sensor histidine kinase	JQC82_RS15700
	HTH domain-containing protein	JQC82_RS15705
	ABC transporter permease	JQC82_RS15710
	CtsR family transcriptional regulator	JQC82_RS15720
	2,3-diphosphoglycerate-dependent phosphoglycerate mutase	JQC82_RS15725
	M13-type metalloendopeptidase	JQC82_RS15740
	GNAT family N-acetyltransferase	JQC82_RS15750
	MarR family transcriptional regulator	JQC82_RS15755
	PTS sugar transporter subunit IIB	JQC82_RS15760
	N-acetylmuramoyl-L-alanine amidase	JQC82_RS15775
	SPFH domain-containing protein	JQC82_RS15790
	Hypothetical protein	JQC82_RS15810
	Phage tail tape measure protein	JQC82_RS15825
	Type II toxin-antitoxin system prevent-host-death family antitoxin	JQC82_RS15830
	Glutamine-hydrolyzing GMP synthase	JQC82_RS15855
	UDP-N-acetylglucosamine 1-carboxyvinyltransferase	JQC82_RS15860
	LPXTG cell wall anchor domain-containing protein	JQC82_RS15865
	Bifunctional DNA primase/polymerase	JQC82_RS15870
	PTS transporter subunit EIIC	JQC82_RS15875
	Hypothetical protein	JQC82_RS15880
	Preprotein translocase subunit SecY	JQC82_RS15885
	Rhamnulose-1-phosphate aldolase	JQC82_RS15890
	Oligosaccharide flippase family protein	JQC82_RS15895
	HAD family hydrolase	JQC82_RS15910
	D-aminoacyl-tRNA deacylase	JQC82_RS15915
	Bifunctional (p)ppGpp synthetase/guanosine-3,5-bis(diphosphate) 3-pyrophosphohydrolase	JQC82_RS15925
	LemA family protein	JQC82_RS15935
	LamG domain-containing protein	JQC82_RS15955
	Orotidine-5-phosphate decarboxylase	JQC82_RS16000

Strain	Product	Gene Locus
	L-serine ammonia-lyase, iron-sulfur-dependent subunit beta	JQC82_RS16015
	CrcB family protein	JQC82_RS16030
	N-acetyldiaminopimelate deacetylase	JQC82_RS16035
	2,3,4,5-tetrahydropyridine-2,6-dicarboxylate N-acetyltransferase	JQC82_RS16040
	Hypothetical protein	JQC82_RS16045
	Hypothetical protein	JQC82_RS16050
	Fructosamine kinase family protein	JQC82_RS16060
	WxL domain-containing protein	JQC82_RS16065
	MurR/RpiR family transcriptional regulator	JQC82_RS16070
	ATP-dependent Clp protease ATP-binding subunit	JQC82_RS16075
	Ribose-5-phosphate isomerase RpiA	JQC82_RS16080
	NADPH-dependent FMN reductase	JQC82_RS16095
	Type I 3-dehydroquinate dehydratase	JQC82_RS16100
	PTS transporter subunit EIIC	JQC82_RS16105
	Hypothetical protein	JQC82_RS16110
	Matrixin family metalloprotease	JQC82_RS16115
	Glycosyltransferase	JQC82_RS16120
	Hypothetical protein	JQC82_RS16125
	Hypothetical protein	JQC82_RS16130
	Hypothetical protein	JQC82_RS16135
	Hypothetical protein	JQC82_RS16140
	Hypothetical protein	JQC82_RS16145
	Protein rep	JQC82_RS16150
	Hypothetical protein	JQC82_RS16190
MF1298	Cold-shock protein	ASV54_RS00530
	DNA/RNA non-specific endonuclease	ASV54_RS01195
	AEC family transporter	ASV54_RS01635
	NAD(P)H-binding protein	ASV54_RS01650
	Alpha/beta hydrolase-fold protein	ASV54_RS01710
	Phosphoketolase family protein	ASV54_RS01715
	Aspartate--ammonia ligase	ASV54_RS01720
	Asparagine--tRNA ligase	ASV54_RS01725
	FMN-dependent NADH-azoreductase	ASV54_RS01730
	IS256 family transposase	ASV54_RS01735
	30S ribosomal protein S1	ASV54_RS01775
	Ketopantoate reductase family protein	ASV54_RS04785
	ATP-dependent sacrificial sulfur transferase LarE	ASV54_RS04790
	Lysis protein	ASV54_RS04800
	Putative holin-like toxin	ASV54_RS04810
	Prepilin peptidase	ASV54_RS04855
	GNAT family protein	ASV54_RS05460
	GntR family transcriptional regulator	ASV54_RS05500
	Biotin carboxylase N-terminal domain-containing protein	ASV54_RS05510
	(d)CMP kinase	ASV54_RS05515
	Amidohydrolase family protein	ASV54_RS05555
	Hypothetical protein	ASV54_RS05715
	GNAT family N-acetyltransferase	ASV54_RS05720
	Hypothetical protein	ASV54_RS05725
	PspC domain-containing protein	ASV54_RS06585
	CPBP family intramembrane metalloprotease	ASV54_RS07280
	ATP-binding cassette domain-containing protein	ASV54_RS07320
	Mevalonate kinase	ASV54_RS08210
	DUF2255 family protein	ASV54_RS08320
	DUF2798 domain-containing protein	ASV54_RS08325
	Cof-type HAD-IIB family hydrolase	ASV54_RS08330
	Cof-type HAD-IIB family hydrolase	ASV54_RS08910
	DTDP-glucose 4,6-dehydratase	ASV54_RS09050
	GNAT family N-acetyltransferase	ASV54_RS09225

Strain	Product	Gene Locus
	YbgA family protein	ASV54_RS10330
	Recombinase family protein	ASV54_RS10335
	Hypothetical protein	ASV54_RS10435
	DUF1398 family protein	ASV54_RS10455
	Hypothetical protein	ASV54_RS10460
	Helix-turn-helix transcriptional regulator	ASV54_RS10465
	Hypothetical protein	ASV54_RS10930
	Ectonucleotide pyrophosphatase/phosphodiesterase	ASV54_RS11090
	Excinuclease ABC subunit UvrA	ASV54_RS11890
	Hypothetical protein	ASV54_RS12150
	Hypothetical protein	ASV54_RS12265
	MFS transporter	ASV54_RS12625
	Energy-coupling factor ABC transporter ATP-binding protein	ASV54_RS13155
	FAD:protein FMN transferase	ASV54_RS13160
	Hypothetical protein	ASV54_RS13355
	LysM domain-containing protein	ASV54_RS13405
	Ribonucleoside hydrolase RihC	ASV54_RS13410
	LacI family DNA-binding transcriptional regulator	ASV54_RS13415
	ABC transporter ATP-binding protein	ASV54_RS13420
	ECF-type riboflavin transporter substrate-binding protein	ASV54_RS13425
	Hypothetical protein	ASV54_RS13430
	Phage tail tip lysozyme	ASV54_RS13435
	Hypothetical protein	ASV54_RS13440
	Oxidoreductase	ASV54_RS13445
	ABC transporter substrate-binding protein	ASV54_RS13450
	Mechanosensitive ion channel family protein	ASV54_RS13455
	Hypothetical protein	ASV54_RS13460
	LacI family DNA-binding transcriptional regulator	ASV54_RS13465
	Hypothetical protein	ASV54_RS13470
	Beta-galactosidase	ASV54_RS13480
	IS30-like element ISLsa1 family transposase	ASV54_RS14455
	DNA mismatch repair protein MutS	ASV54_RS14815
	RecQ family ATP-dependent DNA helicase	ASV54_RS14820
	Nuclear transport factor 2 family protein	ASV54_RS14825
	Gx transporter family protein	ASV54_RS14880
	LysR family transcriptional regulator	ASV54_RS15060
	LysR family transcriptional regulator	ASV54_RS15080
	FAD-dependent oxidoreductase	ASV54_RS15145
	NlpC/P60 family protein	ASV54_RS15240
	ROK family protein	ASV54_RS15245
	Hypothetical protein	ASV54_RS15250
	Major capsid protein	ASV54_RS15255
	Hypothetical protein	ASV54_RS15260
	Pseudouridine synthase	ASV54_RS15265
	Hypothetical protein	ASV54_RS15275
	TetR/AcrR family transcriptional regulator	ASV54_RS15280
	BspA family leucine-rich repeat surface protein	ASV54_RS15290
	Ig-like domain-containing protein	ASV54_RS15605
	ATP-binding protein	ASV54_RS15615
	YibE/F family protein	ASV54_RS15620
	Alkaline shock response membrane anchor protein AmaP	ASV54_RS15625
	Hypothetical protein	ASV54_RS15630
	Energy-coupling factor transporter transmembrane component T	ASV54_RS15635
	Primase C-terminal domain-containing protein	ASV54_RS15640
	Hypothetical protein	ASV54_RS15645
	SMC-Scp complex subunit ScpB	ASV54_RS15650
	Hypothetical protein	ASV54_RS15655
	Hypothetical protein	ASV54_RS15660

Strain	Product	Gene Locus
	Hypothetical protein	ASV54_RS15665
	Helix-turn-helix domain-containing protein	ASV54_RS15670
	Trehalose operon repressor	ASV54_RS15685
	Bacteriocin immunity protein	ASV54_RS15745
	Serine--tRNA ligase	ASV54_RS15760
	Segregation/condensation protein A	ASV54_RS15770
	Hypothetical protein	ASV54_RS15780
	30S ribosomal protein S5	ASV54_RS15790
	Hypothetical protein	ASV54_RS15795
	Hypothetical protein	ASV54_RS15810
	Hypothetical protein	ASV54_RS15815
	VanZ family protein	ASV54_RS15820
	Hypothetical protein	ASV54_RS15825
	MucBP domain-containing protein	ASV54_RS15835
	Putative metal homeostasis protein	ASV54_RS15840
	Helix-turn-helix transcriptional regulator	ASV54_RS15845
	Hypothetical protein	ASV54_RS15850
	Sulfite exporter TauE/SafE family protein	ASV54_RS15865
	Phosphoglycerate dehydrogenase	ASV54_RS15870
	Primase C-terminal domain-containing protein	ASV54_RS15875
	Plasmid recombination protein	ASV54_RS15880
	HAD family phosphatase	ASV54_RS15890
	MFS transporter	ASV54_RS15910
	CrcB family protein	ASV54_RS16015
	TRNA pseudouridine(38-40) synthase TruA	ASV54_RS16095
	Hypothetical protein	ASV54_RS16100
	Hypothetical protein	ASV54_RS16105
	Hypothetical protein	ASV54_RS16110
	Hypothetical protein	ASV54_RS16115
	Hypothetical protein	ASV54_RS16120
	Hypothetical protein	ASV54_RS16125
	50S ribosomal protein L13	ASV54_RS16130
	Hypothetical protein	ASV54_RS16135
	Hypothetical protein	ASV54_RS16140
	YibE/F family protein	ASV54_RS16145
	SGNH/GDSL hydrolase family protein	ASV54_RS16150
	Hypothetical protein	ASV54_RS16155
	Hypothetical protein	ASV54_RS16160
	DUF1648 domain-containing protein	ASV54_RS16170
	Rhamnulokinase	ASV54_RS16180
	C69 family dipeptidase	ASV54_RS16185
	Hypothetical protein	ASV54_RS16190
	Site-specific tyrosine recombinase XerD	ASV54_RS16195
	NAD-dependent protein deacylase	ASV54_RS16210
	AzlC family ABC transporter permease	ASV54_RS16255
	Hypothetical protein	ASV54_RS16275
	MFS transporter	ASV54_RS16280
	Hypothetical protein	ASV54_RS16285
	TSUP family transporter	ASV54_RS16315
	4-hydroxy-tetrahydrodipicolinate synthase	ASV54_RS16320
	Helix-turn-helix domain-containing protein	ASV54_RS16325
	LysR family transcriptional regulator	ASV54_RS16330
	GntR family transcriptional regulator	ASV54_RS16355
	Glycyl radical protein	ASV54_RS16370
	Diadenylate cyclase CdaA	ASV54_RS16400
	Hypothetical protein	ASV54_RS16440
	Zinc ribbon domain-containing protein	ASV54_RS16445
	Acetyl-CoA carboxylase carboxyltransferase subunit beta	ASV54_RS16460

Strain	Product	Gene Locus
	ABC-2 transporter permease	ASV54_RS16470
	MutH/Sau3AI family endonuclease	ASV54_RS16525
	N4-gp56 family major capsid protein	ASV54_RS16530
	ATP-binding protein	ASV54_RS16545
	Helix-turn-helix domain-containing protein	ASV54_RS16555
	Amino acid ABC transporter permease	ASV54_RS16560
	Phage tail protein	ASV54_RS16565
	TrsD/TraD family conjugative transfer protein	ASV54_RS16570
	DHA2 family efflux MFS transporter permease subunit	ASV54_RS16575
	FAD-dependent oxidoreductase	ASV54_RS16580
	MFS transporter	ASV54_RS16585
	YxeA family protein	ASV54_RS16605
	PTS sugar transporter subunit IIB	ASV54_RS16620
	Putative quinol monooxygenase	ASV54_RS16625
	L,D-transpeptidase	ASV54_RS16635
	Hypothetical protein	ASV54_RS16660
	S1-like domain-containing RNA-binding protein	ASV54_RS16665
	Hypothetical protein	ASV54_RS16670
	AAA family ATPase	ASV54_RS16680
	Aminotransferase class I/II-fold pyridoxal phosphate-dependent enzyme	ASV54_RS16710
	DUF441 domain-containing protein	ASV54_RS16715
	Sugar O-acetyltransferase	ASV54_RS16735
	IS3 family transposase	ASV54_RS16740
	Terminase large subunit	ASV54_RS16745
	Single-stranded DNA-binding protein	ASV54_RS16755
	Hypothetical protein	ASV54_RS16775
	MFS transporter	ASV54_RS16785
	6-phosphofructokinase	ASV54_RS16800
	50S ribosomal protein L14	ASV54_RS16810
	Hypothetical protein	ASV54_RS16815
	PD-(D/E)XK nuclease-like domain-containing protein	ASV54_RS16820
	Type 1 glycerol-3-phosphate oxidase	ASV54_RS16835
	Rrf2 family transcriptional regulator	ASV54_RS16840
	Nitronate monooxygenase	ASV54_RS16845
	ABC transporter ATP-binding protein	ASV54_RS16850
	MurR/RpiR family transcriptional regulator	ASV54_RS16855
	Hypothetical protein	ASV54_RS16860
	DNA polymerase III subunit alpha	ASV54_RS16905
	Hypothetical protein	ASV54_RS16910
	HAD hydrolase-like protein	ASV54_RS16915
	Asparagine synthase (glutamine-hydrolyzing)	ASV54_RS16920
	ABC transporter permease	ASV54_RS16925
	ABC transporter ATP-binding protein	ASV54_RS16930
	DUF2252 domain-containing protein	ASV54_RS16935
	MarR family winged helix-turn-helix transcriptional regulator	ASV54_RS16950
	YjzD family protein	ASV54_RS16955
	MarR family transcriptional regulator	ASV54_RS16960
	AAA family ATPase	ASV54_RS16965
	Amino acid ABC transporter ATP-binding protein	ASV54_RS16970
	Immunity 63 family protein	ASV54_RS16975
	Hypothetical protein	ASV54_RS16980
	ATP-dependent chaperone ClpB	ASV54_RS16985
	Potassium-transporting ATPase subunit KdpC	ASV54_RS16990
	Phage antirepressor KilAC domain-containing protein	ASV54_RS16995
	Ribonucleotide-diphosphate reductase subunit beta	ASV54_RS17000
	Hypothetical protein	ASV54_RS17005
	Response regulator transcription factor	ASV54_RS17010
	Restriction endonuclease subunit S	ASV54_RS17015

Strain	Product	Gene Locus
	Alpha/beta hydrolase	ASV54_RS17020
	Peptidase T	ASV54_RS17025
	PTS glucose transporter subunit IIABC	ASV54_RS17060
	MerR family transcriptional regulator	ASV54_RS17075
	ABC transporter ATP-binding protein	ASV54_RS17080
	Hypothetical protein	ASV54_RS17085
	DUF4355 domain-containing protein	ASV54_RS17090
	Nif3-like dinuclear metal center hexameric protein	ASV54_RS17115
	L-lactate permease	ASV54_RS17120
	GNAT family N-acetyltransferase	ASV54_RS17125
	FAD:protein FMN transferase	ASV54_RS17130
	PTS beta-glucoside transporter subunit IIBCA	ASV54_RS17135
	LCP family protein	ASV54_RS17140
	Hypothetical protein	ASV54_RS17150
	30S ribosomal protein S3	ASV54_RS17155
	LytTR family DNA-binding domain-containing protein	ASV54_RS17160
	Beta-glucoside-specific PTS transporter subunit IIABC	ASV54_RS17165
	DeoR/GlpR family DNA-binding transcription regulator	ASV54_RS17170
	Dihydroorotate dehydrogenase	ASV54_RS17175
	M15 family metallopeptidase	ASV54_RS17180
	Y-family DNA polymerase	ASV54_RS17190
	DeoR/GlpR family DNA-binding transcription regulator	ASV54_RS17195
	MarR family transcriptional regulator	ASV54_RS17215
	D-ribose pyranase	ASV54_RS17220
	YueI family protein	ASV54_RS17225
	Restriction endonuclease subunit S	ASV54_RS17265
	Type VII secretion protein EssB/YukC	ASV54_RS17280
	TRNA (adenine(22)-N(1))-methyltransferase TrmK	ASV54_RS17285
	Phage terminase small subunit P27 family	ASV54_RS17320
	Hypothetical protein	ASV54_RS17325
	Histidine phosphatase family protein	ASV54_RS17330
	Hypothetical protein	ASV54_RS17335
	Carboxyltransferase subunit alpha	ASV54_RS17360
	MurR/RpiR family transcriptional regulator	ASV54_RS17365
	Hypothetical protein	ASV54_RS17375
	Cobalt transporter CbiM	ASV54_RS17380
	Site-specific integrase	ASV54_RS17405
	DNA-directed RNA polymerase subunit beta	ASV54_RS17415
	PAS domain-containing protein	ASV54_RS17420
	O-antigen ligase family protein	ASV54_RS17430
	Multidrug effflux MFS transporter	ASV54_RS17435
	Hsp20/alpha crystallin family protein	ASV54_RS17440
	ABC transporter permease	ASV54_RS17445
	Crp/Fnr family transcriptional regulator	ASV54_RS17455
	NAD(P)H-dependent oxidoreductase	ASV54_RS17465
	MarR family winged helix-turn-helix transcriptional regulator	ASV54_RS17470
	6-phospho-beta-glucosidase	ASV54_RS17475
	Hypothetical protein	ASV54_RS17485
	Thiamine pyrophosphate-binding protein	ASV54_RS17490
	Phage portal protein	ASV54_RS17495
	Phosphoenolpyruvate synthase	ASV54_RS17505
	Hypothetical protein	ASV54_RS17515
	Rhodanese-like domain-containing protein	ASV54_RS17545
	Hypothetical protein	ASV54_RS17560
	Acyltransferase	ASV54_RS17590
	KUP/HAK/KT family potassium transporter	ASV54_RS17605
	Zinc-binding alcohol dehydrogenase family protein	ASV54_RS17620
	Sulfite exporter TauE/SafE family protein	ASV54_RS17645

Strain	Product	Gene Locus
	NUDIX hydrolase N-terminal domain-containing protein	ASV54_RS17650
	LCP family protein	ASV54_RS17655
	NAD(P)H-binding protein	ASV54_RS17675
	TetR/AcrR family transcriptional regulator	ASV54_RS17680
	HAD-IC family P-type ATPase	ASV54_RS17690
	Gp58-like family protein	ASV54_RS17705
	Phosphopyruvate hydratase	ASV54_RS17745
RI-113	Dihydroxyacetone kinase phosphoryl donor subunit DhaM	BJD88_RS00005
	DHA2 family efflux MFS transporter permease subunit	BJD88_RS00010
	Aldo/keto reductase	BJD88_RS00015
	Asp23/Gls24 family envelope stress response protein	BJD88_RS00020
	Hypothetical protein	BJD88_RS00030
	AAA family ATPase	BJD88_RS00035
	Glycerol kinase GlpK	BJD88_RS00040
	Phosphoribosylformylglycinamidine synthase subunit PurQ	BJD88_RS00045
	Nickel-dependent lactate racemase	BJD88_RS02355
	Metal ABC transporter substrate-binding protein	BJD88_RS02360
	Alpha-rhamnosidase	BJD88_RS02365
	Hypothetical protein	BJD88_RS03085
	ATP phosphoribosyltransferase regulatory subunit	BJD88_RS03695
	D-serine ammonia-lyase	BJD88_RS04910
	4-oxalocrotonate tautomerase	BJD88_RS05565
	Hypothetical protein	BJD88_RS05910
	Histidine phosphatase family protein	BJD88_RS06370
	Hypothetical protein	BJD88_RS06965
	Metallophosphoesterase	BJD88_RS06980
	DUF1002 domain-containing protein	BJD88_RS07610
	[citrate (pro-3S)-lyase] ligase	BJD88_RS07615
	Hypothetical protein	BJD88_RS07880
	YozE family protein	BJD88_RS07920
	YpmS family protein	BJD88_RS07925
	Hypothetical protein	BJD88_RS08005
	DUF3775 domain-containing protein	BJD88_RS08015
	DUF4428 domain-containing protein	BJD88_RS08110
	DUF4430 domain-containing protein	BJD88_RS08115
	MarR family transcriptional regulator	BJD88_RS08190
	Sugar phosphate isomerase/epimerase	BJD88_RS08215
	Enolase C-terminal domain-like protein	BJD88_RS08230
	Cof-type HAD-IIB family hydrolase	BJD88_RS08235
	Hypothetical protein	BJD88_RS10275
	Capsid protein	BJD88_RS10345
	Peptide-methionine (R)-S-oxide reductase MsrB	BJD88_RS10350
	Manganese-dependent inorganic pyrophosphatase	BJD88_RS10355
	LysR family transcriptional regulator	BJD88_RS10360
	Hypothetical protein	BJD88_RS10365
	VanZ family protein	BJD88_RS10375
	DUF3324 domain-containing protein	BJD88_RS10390
	Cof-type HAD-IIB family hydrolase	BJD88_RS10400
	Alpha/beta hydrolase	BJD88_RS10410
	TRNA uridine-5-carboxymethylaminomethyl(34) synthesis enzyme MnmG	BJD88_RS10415
	Hypothetical protein	BJD88_RS10420
	Hypothetical protein	BJD88_RS10425
	4-hydroxyphenylacetate 3-hydroxylase N-terminal domain-containing protein	BJD88_RS10430
	Cof-type HAD-IIB family hydrolase	BJD88_RS10435
	LacI family DNA-binding transcriptional regulator	BJD88_RS10440
	MurR/RpiR family transcriptional regulator	BJD88_RS10445
	Gfo/Idh/MocA family oxidoreductase	BJD88_RS10450

Strain	Product	Gene Locus
	Gfo/Idh/MocA family oxidoreductase	BJD88_RS10455
	Na+/H+ antiporter	BJD88_RS10460
	1-deoxy-D-xylulose-5-phosphate synthase	BJD88_RS10465
	Isochorismatase family cysteine hydrolase	BJD88_RS10470
	N-acetylmannosamine-6-phosphate 2-epimerase	BJD88_RS10475
	ABC transporter permease	BJD88_RS10480
	ABC transporter ATP-binding protein	BJD88_RS10485
	Hypothetical protein	BJD88_RS10490
	C1 family peptidase	BJD88_RS10495
	50S ribosomal protein L34	BJD88_RS10545
	Multicopper oxidase domain-containing protein	BJD88_RS10560
	Hypothetical protein	BJD88_RS10565
	Pentapeptide repeat-containing protein	BJD88_RS10570
	Hypothetical protein	BJD88_RS10585
	Polyphosphate kinase 2 family protein	BJD88_RS10590
	Nucleoside hydrolase	BJD88_RS10595
	Nitroreductase family protein	BJD88_RS10600
	DUF1801 domain-containing protein	BJD88_RS10605
	Alpha-galactosidase	BJD88_RS10610
	Sugar kinase	BJD88_RS10615
	Lrp/AsnC family transcriptional regulator	BJD88_RS10625
	Hypothetical protein	BJD88_RS10635
	DNA topoisomerase IV subunit A	BJD88_RS10640
	50S ribosomal protein L18	BJD88_RS10645
	LytTR family transcriptional regulator	BJD88_RS10935
	Hypothetical protein	BJD88_RS10990
	TRNA (adenosine(37)-N6)-threonylcarbamoyltransferase complex dimerization subunit type 1 TsaB	BJD88_RS11040
	Phage tail family protein	BJD88_RS11045
	Glycosyltransferase	BJD88_RS11060
	ABC transporter ATP-binding protein	BJD88_RS11065
	YhgE/Pip family protein	BJD88_RS11075
	LLM class flavin-dependent oxidoreductase	BJD88_RS11080
	Aldo/keto reductase	BJD88_RS11090
	DNA topoisomerase IV subunit B	BJD88_RS11095
	Hypothetical protein	BJD88_RS11110
	Hypothetical protein	BJD88_RS11385
	MHS family MFS transporter	BJD88_RS13855
	Bacteriocin immunity protein	BJD88_RS13890
	MFS transporter	BJD88_RS13905
	MFS transporter	BJD88_RS13910
	MFS transporter	BJD88_RS13915
	MFS transporter	BJD88_RS13920
	MFS transporter	BJD88_RS13925
	MFS transporter	BJD88_RS13930
	MFS transporter	BJD88_RS13940
	MFS transporter	BJD88_RS13945
	MFS transporter	BJD88_RS13950
	MFS transporter	BJD88_RS13955
	MFS transporter	BJD88_RS13960
	Fructokinase	BJD88_RS14200
	Nucleoside 2-deoxyribosyltransferase	BJD88_RS14205
	Amidophosphoribosyltransferase	BJD88_RS14620
	Histidine phosphatase family protein	BJD88_RS14655
	YdcF family protein	BJD88_RS14810
	Transcriptional regulator Spx	BJD88_RS14885
	MerR family transcriptional regulator	BJD88_RS14890
	ATP-dependent DNA helicase	BJD88_RS14895

Strain	Product	Gene Locus
	Phosphoglycerate mutase family protein	BJD88_RS14900
	Hypothetical protein	BJD88_RS14910
	Hypothetical protein	BJD88_RS14915
	NusG domain II-containing protein	BJD88_RS14925
	Phosphate ABC transporter permease PstA	BJD88_RS14930
	ABC transporter ATP-binding protein	BJD88_RS14935
	Sugar O-acetyltransferase	BJD88_RS14940
	ABC-F family ATP-binding cassette domain-containing protein	BJD88_RS14945
	Ribosome biogenesis GTPase Der	BJD88_RS15740
	IS30-like element ISLsa1 family transposase	BJD88_RS15860
	Alpha-amylase family glycosyl hydrolase	BJD88_RS15905
	Glycoside hydrolase family 65 protein	BJD88_RS15910
	Hypothetical protein	BJD88_RS15955
	Hypothetical protein	BJD88_RS15960
	M20/M25/M40 family metallo-hydrolase	BJD88_RS15975
	Hypothetical protein	BJD88_RS15985
	DUF916 and DUF3324 domain-containing protein	BJD88_RS15995
	Zinc-binding dehydrogenase	BJD88_RS16000
	Glycerol-3-phosphate 1-O-acyltransferase PlsY	BJD88_RS16010
	YtxH domain-containing protein	BJD88_RS16025
	PTS glucose transporter subunit IIA	BJD88_RS16070
	MarR family transcriptional regulator	BJD88_RS16075
	Hypothetical protein	BJD88_RS16080
	Hypothetical protein	BJD88_RS16085
	Aldose 1-epimerase family protein	BJD88_RS16095
	Hypothetical protein	BJD88_RS16120
	Iron export ABC transporter permease subunit FetB	BJD88_RS16140
	Excinuclease ABC subunit UvrB	BJD88_RS16145
	Helix-turn-helix transcriptional regulator	BJD88_RS16150
	ABC transporter transmembrane domain-containing protein	BJD88_RS16155
	Hypothetical protein	BJD88_RS16160
	ATP-dependent Clp protease ATP-binding subunit	BJD88_RS16165
	PTS sugar transporter subunit IIB	BJD88_RS16170
	PBSX family phage terminase large subunit	BJD88_RS16175
	ATP-binding cassette domain-containing protein	BJD88_RS16200
	Glycosyltransferase family 2 protein	BJD88_RS16215
	Zinc-binding alcohol dehydrogenase family protein	BJD88_RS16220
	DUF2187 domain-containing protein	BJD88_RS16230
	Pyridoxamine 5-phosphate oxidase family protein	BJD88_RS16245
	IS256 family transposase	BJD88_RS16250
	Hypothetical protein	BJD88_RS16270
	Helix-turn-helix domain-containing protein	BJD88_RS16275
	Hypothetical protein	BJD88_RS16280
	Alpha-glucosidase	BJD88_RS16295
	Hypothetical protein	BJD88_RS16300
	Metal-sulfur cluster assembly factor	BJD88_RS16305
	DNA-directed RNA polymerase subunit delta	BJD88_RS16315
	VanZ family protein	BJD88_RS16320
	Spx/MgsR family RNA polymerase-binding regulatory protein	BJD88_RS16340
	Hypothetical protein	BJD88_RS16345
	FAD-dependent oxidoreductase	BJD88_RS16370
	Terminase large subunit	BJD88_RS16380
	ParA family protein	BJD88_RS16385
	Lactate oxidase	BJD88_RS16390
	Alpha-mannosidase	BJD88_RS16395
	Hypothetical protein	BJD88_RS16400
	SGNH/GDSL hydrolase family protein	BJD88_RS16430
	Transcriptional regulator GutM	BJD88_RS16435

Strain	Product	Gene Locus
	23S rRNA (uracil(1939)-C(5))-methyltransferase RlmD	BJD88_RS16450
	DNA-binding protein WhiA	BJD88_RS16465
	Hypothetical protein	BJD88_RS16475
	Hypothetical protein	BJD88_RS16480
	Hypothetical protein	BJD88_RS16485
	Fumarylacetoacetate hydrolase family protein	BJD88_RS16490
	ATP-dependent protease ATPase subunit HslU	BJD88_RS16510
	MIP/aquaporin family protein	BJD88_RS16515
	Asp23/Gls24 family envelope stress response protein	BJD88_RS16530
	M1 family metallopeptidase	BJD88_RS16545
	Glutamate decarboxylase	BJD88_RS16550
	Replication initiator protein A	BJD88_RS16555
	Putative HNHc nuclease	BJD88_RS16570
	SpaA isopeptide-forming pilin-related protein	BJD88_RS16575
	DNA starvation/stationary phase protection protein	BJD88_RS16580
	ISL3-like element ISP1 family transposase	BJD88_RS16590
	Hypothetical protein	BJD88_RS16595
	Hypothetical protein	BJD88_RS16600
	Type I DNA topoisomerase	BJD88_RS16605
	FAD-dependent oxidoreductase	BJD88_RS16630
	DegV family protein	BJD88_RS16635
	DNA-processing protein DprA	BJD88_RS16670
	Dihydrofolate reductase	BJD88_RS16675
	AzlD domain-containing protein	BJD88_RS16685
	Bifunctional DNA primase/polymerase	BJD88_RS16710
	Bacterial Ig-like domain-containing protein	BJD88_RS16715
	Ribonuclease HII	BJD88_RS16825
	DNA-3-methyladenine glycosylase I	BJD88_RS16835
	3-5 exonuclease	BJD88_RS16840
	Ribosome biogenesis GTPase YlqF	BJD88_RS16850
	PadR family transcriptional regulator	BJD88_RS16860
	4-hydroxy-tetrahydrodipicolinate reductase	BJD88_RS16865
	Deoxynucleoside kinase	BJD88_RS16880
	Biotin/lipoyl-containing protein	BJD88_RS16885
	Hypothetical protein	BJD88_RS16940
	Type 1 glutamine amidotransferase domain-containing protein	BJD88_RS16945
	NADP-dependent oxidoreductase	BJD88_RS16950
	Metalloregulator ArsR/SmtB family transcription factor	BJD88_RS16955
	MATE family efflux transporter	BJD88_RS16960
	Hypothetical protein	BJD88_RS16965
	Cobalamin-independent methionine synthase II family protein	BJD88_RS16970
	Single-stranded DNA-binding protein	BJD88_RS16990
	C69 family dipeptidase	BJD88_RS16995
	Translation initiation factor IF-1	BJD88_RS17000
	VanZ family protein	BJD88_RS17005
	Hypothetical protein	BJD88_RS17015
	LacI family DNA-binding transcriptional regulator	BJD88_RS17035
	Metalloregulator ArsR/SmtB family transcription factor	BJD88_RS17065
	ROK family protein	BJD88_RS17075
	Thymidylate synthase	BJD88_RS17090
	LysR family transcriptional regulator	BJD88_RS17135
	Nucleoside-diphosphate kinase	BJD88_RS17155
	Helix-turn-helix transcriptional regulator	BJD88_RS17160
	Cell surface protein	BJD88_RS17175
	Hypothetical protein	BJD88_RS17185
	DUF948 domain-containing protein	BJD88_RS17210
	SDR family oxidoreductase	BJD88_RS17215
	Phytoene/squalene synthase family protein	BJD88_RS17245

Strain	Product	Gene Locus
	Alpha/beta hydrolase	BJD88_RS17300
	Glycerate kinase	BJD88_RS17335
	Terminase small subunit	BJD88_RS17370
	Hypothetical protein	BJD88_RS17405
	Ldh family oxidoreductase	BJD88_RS17410
	Ketoacyl-ACP synthase III	BJD88_RS17420
	C40 family peptidase	BJD88_RS17430
	Serine hydroxymethyltransferase	BJD88_RS17435
	S41 family peptidase	BJD88_RS17440
	Peptide chain release factor N(5)-glutamine methyltransferase	BJD88_RS17455
	MFS transporter	BJD88_RS17475
	Cof-type HAD-IIB family hydrolase	BJD88_RS17490
	Histidine phosphatase family protein	BJD88_RS17495
	Bifunctional DNA primase/polymerase	BJD88_RS17520
	SRPBCC family protein	BJD88_RS17565
	Asparagine synthase (glutamine-hydrolyzing)	BJD88_RS17570
	Hypothetical protein	BJD88_RS17575
	Alpha-glucosidase	BJD88_RS17580
	Tetratricopeptide repeat protein	BJD88_RS17590
	Preprotein translocase subunit SecA	BJD88_RS17605
	Bacterial Ig-like domain-containing protein	BJD88_RS17625
	Restriction endonuclease	BJD88_RS17635
	Hypothetical protein	BJD88_RS17645
	L-threonylcarbamoyladenylate synthase	BJD88_RS17660
	Histidinol-phosphatase HisJ	BJD88_RS17670
	HU family DNA-binding protein	BJD88_RS17685
	NAD(P)-binding domain-containing protein	BJD88_RS17720
	Hypothetical protein	BJD88_RS17725
BLS41	Chloramphenicol acetyltransferase CAT	BOQ65_RS00005
	GIY-YIG nuclease family protein	BOQ65_RS00010
	Nitroreductase	BOQ65_RS00020
	DUF2087 domain-containing protein	BOQ65_RS00025
	NUDIX hydrolase	BOQ65_RS00030
	Threonine/serine exporter family protein	BOQ65_RS00035
	Aldose epimerase family protein	BOQ65_RS00040
	Thioredoxin family protein	BOQ65_RS00050
	ABC transporter permease	BOQ65_RS00055
	NFACT RNA binding domain-containing protein	BOQ65_RS00060
	MarR family transcriptional regulator	BOQ65_RS00065
	WxL domain-containing protein	BOQ65_RS00070
	Transposase	BOQ65_RS00075
	LysR family transcriptional regulator	BOQ65_RS00515
	NADPH-dependent FMN reductase	BOQ65_RS00520
	Flavocytochrome c	BOQ65_RS00525
	Hypothetical protein	BOQ65_RS00530
	Nucleoside 2-deoxyribosyltransferase	BOQ65_RS00535
	Glycosyltransferase family 2 protein	BOQ65_RS00540
	Hypothetical protein	BOQ65_RS00545
	NUDIX hydrolase	BOQ65_RS00550
	Riboflavin synthase	BOQ65_RS00565
	Bifunctional 3,4-dihydroxy-2-butanone-4-phosphate synthase/GTP cyclohydrolase II	BOQ65_RS00570
	6,7-dimethyl-8-ribityllumazine synthase	BOQ65_RS00575
	Hypothetical protein	BOQ65_RS00580
	Crp/Fnr family transcriptional regulator	BOQ65_RS00585
	TetR family transcriptional regulator	BOQ65_RS00590
	WxL domain-containing protein	BOQ65_RS00595
	LPXTG cell wall anchor domain-containing protein	BOQ65_RS00600

Strain	Product	Gene Locus
	DUF916 and DUF3324 domain-containing protein	BOQ65_RS00605
	WxL domain-containing protein	BOQ65_RS00610
	WxL domain-containing protein	BOQ65_RS00615
	Peptidylprolyl isomerase	BOQ65_RS00620
	YtxH domain-containing protein	BOQ65_RS00625
	HIT family protein	BOQ65_RS00630
	ABC transporter ATP-binding protein	BOQ65_RS00635
	ABC transporter permease	BOQ65_RS00640
	Phosphotransferase family protein	BOQ65_RS00645
	TRNA (guanosine(46)-N7)-methyltransferase TrmB	BOQ65_RS00650
	PepSY domain-containing protein	BOQ65_RS00655
	DUF4479 and tRNA-binding domain-containing protein	BOQ65_RS00660
	UDP-N-acetylmuramate--L-alanine ligase	BOQ65_RS00670
	Ferrous iron transport protein B	BOQ65_RS00680
	Transglycosylase domain-containing protein	BOQ65_RS01285
	Hypothetical protein	BOQ65_RS01500
	Helix-turn-helix transcriptional regulator	BOQ65_RS03060
	Glutamate 5-kinase	BOQ65_RS03155
	IS3 family transposase	BOQ65_RS03160
	DUF4868 domain-containing protein	BOQ65_RS03190
	DNA repair protein RecO	BOQ65_RS03210
	DUF536 domain-containing protein	BOQ65_RS03240
	RRNA maturation RNase YbeY	BOQ65_RS03270
	PhoH family protein	BOQ65_RS03275
	Hypothetical protein	BOQ65_RS03280
	Aldo/keto reductase	BOQ65_RS03290
	Alpha/beta hydrolase	BOQ65_RS03295
	LCP family protein	BOQ65_RS03310
	Hypothetical protein	BOQ65_RS03875
	GatB/YqeY domain-containing protein	BOQ65_RS04805
	30S ribosomal protein S21	BOQ65_RS04810
	Hypothetical protein	BOQ65_RS04955
	Capsid protein	BOQ65_RS05085
	Transcriptional regulator	BOQ65_RS05110
	LysR family transcriptional regulator	BOQ65_RS05350
	Hypothetical protein	BOQ65_RS05500
	Lipoprotein	BOQ65_RS06155
	Hypothetical protein	BOQ65_RS06250
	Peptide deformylase	BOQ65_RS06345
	Pyruvate, water dikinase regulatory protein	BOQ65_RS07595
	Hypothetical protein	BOQ65_RS07605
	FAD-linked oxidase C-terminal domain-containing protein	BOQ65_RS07610
	PTS ascorbate transporter subunit IIC	BOQ65_RS07615
	Acyltransferase	BOQ65_RS07630
	UDP-glucose 4-epimerase GalE	BOQ65_RS07640
	TRNA-dihydrouridine synthase family protein	BOQ65_RS07845
	Hypothetical protein	BOQ65_RS08070
	Helix-turn-helix transcriptional regulator	BOQ65_RS08075
	Hypothetical protein	BOQ65_RS08080
	Hypothetical protein	BOQ65_RS08085
	RNA-binding cell elongation regulator Jag/EloR	BOQ65_RS08090
	Glycosyltransferase	BOQ65_RS08165
	LysR family transcriptional regulator	BOQ65_RS08175
	Kinase	BOQ65_RS08180
	Sugar ABC transporter	BOQ65_RS09335
	SemiSWEET family transporter	BOQ65_RS09340
	GntR family transcriptional regulator	BOQ65_RS09345
	LPXTG cell wall anchor domain-containing protein	BOQ65_RS09355

Strain	Product	Gene Locus
	PTS sugar transporter subunit IIC	BOQ65_RS09360
	GH25 family lysozyme	BOQ65_RS09365
	GIY-YIG nuclease family protein	BOQ65_RS09370
	GNAT family N-acetyltransferase	BOQ65_RS09375
	YjjG family noncanonical pyrimidine nucleotidase	BOQ65_RS09380
	NAD(P)-binding domain-containing protein	BOQ65_RS09390
	Hypothetical protein	BOQ65_RS09395
	Accessory gene regulator AgrB	BOQ65_RS09400
	Glucosamine-6-phosphate deaminase	BOQ65_RS09405
	Restriction endonuclease subunit S	BOQ65_RS09410
	SDR family oxidoreductase	BOQ65_RS09415
	Hypothetical protein	BOQ65_RS09420
	ABC transporter ATP-binding protein	BOQ65_RS09425
	Acyltransferase family protein	BOQ65_RS09430
	Hypothetical protein	BOQ65_RS09435
	OsmC family protein	BOQ65_RS09440
	RNA polymerase recycling motor HelD	BOQ65_RS09450
	Bacteriocin	BOQ65_RS09455
	Hypothetical protein	BOQ65_RS09460
	Zeta toxin family protein	BOQ65_RS09465
	Acetyl-CoA carboxylase biotin carboxyl carrier protein subunit	BOQ65_RS09920
	Hypothetical protein	BOQ65_RS09925
	TatD family hydrolase	BOQ65_RS09930
	Transposase	BOQ65_RS09935
	Thioesterase	BOQ65_RS09940
	Transporter substrate-binding domain-containing protein	BOQ65_RS09945
	Terminase small subunit	BOQ65_RS10870
	Terminase small subunit	BOQ65_RS10875
	Terminase small subunit	BOQ65_RS10880
	Aldo/keto reductase	BOQ65_RS11220
	ATP-binding cassette domain-containing protein	BOQ65_RS11400
	Aspartate-semialdehyde dehydrogenase	BOQ65_RS11415
	Y-family DNA polymerase	BOQ65_RS11430
	YfhO family protein	BOQ65_RS11435
	YfhO family protein	BOQ65_RS11440
	YhgE/Pip domain-containing protein	BOQ65_RS11445
	Hypothetical protein	BOQ65_RS11480
	Amidohydrolase family protein	BOQ65_RS12000
	PspC domain-containing protein	BOQ65_RS12005
	Gamma-glutamyl-gamma-aminobutyrate hydrolase family protein	BOQ65_RS13420
	Oleate hydratase	BOQ65_RS13425
	Hypothetical protein	BOQ65_RS13430
	HNH endonuclease	BOQ65_RS13575
	Hypothetical protein	BOQ65_RS13580
	Sensor histidine kinase KdpD	BOQ65_RS13585
	AAA family ATPase	BOQ65_RS13590
	AAA family ATPase	BOQ65_RS13600
	Alpha/beta fold hydrolase	BOQ65_RS14310
	ABC transporter permease	BOQ65_RS14600
	ABC transporter permease	BOQ65_RS14605
	ABC transporter permease	BOQ65_RS14610
	Hypothetical protein	BOQ65_RS14615
	Hypothetical protein	BOQ65_RS14620
	Major capsid protein	BOQ65_RS14625
	MerR family transcriptional regulator	BOQ65_RS14630
	Hypothetical protein	BOQ65_RS14640
	Hypothetical protein	BOQ65_RS14645
	Hypothetical protein	BOQ65_RS14650

Strain	Product	Gene Locus
	GntR family transcriptional regulator	BOQ65_RS14655
	Amino acid ABC transporter ATP-binding protein	BOQ65_RS14665
	Hypothetical protein	BOQ65_RS14670
	Hypothetical protein	BOQ65_RS14710
	RNA polymerase sigma factor RpoD	BOQ65_RS14720
	Hypothetical protein	BOQ65_RS14725
	Sugar O-acetyltransferase	BOQ65_RS14760
	Anaerobic ribonucleoside-triphosphate reductase	BOQ65_RS14785
	GtrA family protein	BOQ65_RS14845
	30S ribosomal protein S17	BOQ65_RS15515
	DUF1642 domain-containing protein	BOQ65_RS15520
	DegV family protein	BOQ65_RS15525
	GNAT family N-acetyltransferase	BOQ65_RS15530
	DUF975 family protein	BOQ65_RS15535
	Phage portal protein	BOQ65_RS15540
	Hypothetical protein	BOQ65_RS15545
	RNA-binding protein	BOQ65_RS15550
	CDP-glycerol glycerophosphotransferase family protein	BOQ65_RS15555
	DUF956 family protein	BOQ65_RS15560
	MFS transporter	BOQ65_RS15565
	Amino acid permease	BOQ65_RS15570
	Phage tail protein	BOQ65_RS15575
	DNA replication protein DnaD	BOQ65_RS15580
	Glycosyltransferase family 2 protein	BOQ65_RS15585
	Hypothetical protein	BOQ65_RS15590
	Hypothetical protein	BOQ65_RS15595
	Glycosyltransferase	BOQ65_RS15600
	NAD(P)/FAD-dependent oxidoreductase	BOQ65_RS15605
	DUF960 domain-containing protein	BOQ65_RS15615
	Transposase	BOQ65_RS15620
	DUF1003 domain-containing protein	BOQ65_RS15625
	DUF368 domain-containing protein	BOQ65_RS15630
	Glycine--tRNA ligase subunit alpha	BOQ65_RS15640
	Hypothetical protein	BOQ65_RS15645
	LytTR family transcriptional regulator	BOQ65_RS15685
	Amino acid permease	BOQ65_RS15700
	Hypothetical protein	BOQ65_RS15705
	Hypothetical protein	BOQ65_RS15710
	6-carboxytetrahydropterin synthase	BOQ65_RS15715
	Hypothetical protein	BOQ65_RS15720
	2-C-methyl-D-erythritol 4-phosphate cytidylyltransferase	BOQ65_RS15725
	Tyrosine-protein phosphatase	BOQ65_RS15730
	MBL fold metallo-hydrolase	BOQ65_RS15735
	TIGR02328 family protein	BOQ65_RS15745
	CDP-glycerol glycerophosphotransferase family protein	BOQ65_RS15750
	Minor capsid protein	BOQ65_RS15800
	CDP-glycerol glycerophosphotransferase family protein	BOQ65_RS15805
	2-oxo acid dehydrogenase subunit E2	BOQ65_RS15820
	Transposase	BOQ65_RS15825
	Dihydrolipoyl dehydrogenase	BOQ65_RS15835
	Recombinase RecT	BOQ65_RS15870
	Carboxymuconolactone decarboxylase family protein	BOQ65_RS15890
	Cupin domain-containing protein	BOQ65_RS15905
	Glycine--tRNA ligase subunit beta	BOQ65_RS15920
	Matrixin family metalloprotease	BOQ65_RS15925
	Methyltransferase domain-containing protein	BOQ65_RS15930
	Transposase	BOQ65_RS15965
	KxYKxGKxW signal peptide domain-containing protein	BOQ65_RS16055

Strain	Product	Gene Locus
	GNAT family N-acetyltransferase	BOQ65_RS16060
	Hypothetical protein	BOQ65_RS16075
	Preprotein translocase subunit SecY	BOQ65_RS16080
	Rhamnulose-1-phosphate aldolase	BOQ65_RS16085
	Oligosaccharide flippase family protein	BOQ65_RS16090
	PucR family transcriptional regulator ligand-binding domain-containing protein	BOQ65_RS16110
	IS256 family transposase	BOQ65_RS16180
	GNAT family N-acetyltransferase	BOQ65_RS16260
	GNAT family N-acetyltransferase	BOQ65_RS16290
	PTS sugar transporter subunit IIB	BOQ65_RS16310
	N-acetylmuramoyl-L-alanine amidase	BOQ65_RS16330
	SPFH domain-containing protein	BOQ65_RS16345
	DUF2089 family protein	BOQ65_RS16350
	Hypothetical protein	BOQ65_RS16365
	Phage tail tape measure protein	BOQ65_RS16375
	Type II toxin-antitoxin system prevent-host-death family antitoxin	BOQ65_RS16380
	Glucose-6-phosphate dehydrogenase	BOQ65_RS16400
	Helicase-exonuclease AddAB subunit AddA	BOQ65_RS16415
	Helix-turn-helix domain-containing protein	BOQ65_RS16420
	Hypothetical protein	BOQ65_RS16430
	Hypothetical protein	BOQ65_RS16440
	Orotidine-5-phosphate decarboxylase	BOQ65_RS16460
	L-serine ammonia-lyase, iron-sulfur-dependent subunit beta	BOQ65_RS16475
	Thioredoxin	BOQ65_RS16480
	N-acetyldiaminopimelate deacetylase	BOQ65_RS16495
	2,3,4,5-tetrahydropyridine-2,6-dicarboxylate N-acetyltransferase	BOQ65_RS16500
	Hypothetical protein	BOQ65_RS16505
	Hypothetical protein	BOQ65_RS16510
	Fructosamine kinase family protein	BOQ65_RS16525
	WxL domain-containing protein	BOQ65_RS16530
	Ribose-5-phosphate isomerase RpiA	BOQ65_RS16545
	Glycoside hydrolase family 1 protein	BOQ65_RS16550
	PTS transporter subunit EIIC	BOQ65_RS16565
	PTS transporter subunit EIIC	BOQ65_RS16570
	Hypothetical protein	BOQ65_RS16575
	Matrixin family metalloprotease	BOQ65_RS16580
	Glycosyltransferase	BOQ65_RS16585
	Hypothetical protein	BOQ65_RS16590
	Hypothetical protein	BOQ65_RS16595
	Hypothetical protein	BOQ65_RS16600
	Hypothetical protein	BOQ65_RS16605
	Hypothetical protein	BOQ65_RS16610
	Protein rep	BOQ65_RS16615
	6-phospho-beta-glucosidase	BOQ65_RS16625
	Glycosyltransferase	BOQ65_RS16630
	Hypothetical protein	BOQ65_RS16635
	MarR family transcriptional regulator	BOQ65_RS16640
	NAD(P)/FAD-dependent oxidoreductase	BOQ65_RS16645
	Cellulose biosynthesis cyclic di-GMP-binding regulatory protein BcsB	BOQ65_RS16665
	Translation elongation factor 4	BOQ65_RS16670
	MFS transporter	BOQ65_RS16675
	PTS sugar transporter subunit IIA	BOQ65_RS16680
	Ribokinase	BOQ65_RS16685
	Hypothetical protein	BOQ65_RS16690
	Glycoside-pentoside-hexuronide (GPH):cation symporter	BOQ65_RS16695
	Hypothetical protein	BOQ65_RS16700
	Biotin--[acetyl-CoA-carboxylase] ligase	BOQ65_RS16710

Strain	Product	Gene Locus
	NUDIX domain-containing protein	BOQ65_RS16715
	Shikimate dehydrogenase	BOQ65_RS16720
	Anaerobic ribonucleoside-triphosphate reductase activating protein	BOQ65_RS16725
	UDP-glucose--hexose-1-phosphate uridylyltransferase	BOQ65_RS16735
	Histidine--tRNA ligase	BOQ65_RS16745
	Hypothetical protein	BOQ65_RS16750
	Hypothetical protein	BOQ65_RS16755
	Divalent metal cation transporter	BOQ65_RS16765
	DMT family transporter	BOQ65_RS16770
	Helix-turn-helix transcriptional regulator	BOQ65_RS16780
	NUDIX domain-containing protein	BOQ65_RS16785
	Helix-turn-helix domain-containing protein	BOQ65_RS16795
	Sugar-phosphatase	BOQ65_RS16800
	Aspartate--tRNA ligase	BOQ65_RS16810
	ABC transporter permease	BOQ65_RS16815
	Peptide-methionine (S)-S-oxide reductase MsrA	BOQ65_RS16820
	Hypothetical protein	BOQ65_RS16825
	YitT family protein	BOQ65_RS16830
	SDR family oxidoreductase	BOQ65_RS16835
	Hypothetical protein	BOQ65_RS16840
	Aldose epimerase family protein	BOQ65_RS16845
	Hypothetical protein	BOQ65_RS16850
	Hypothetical protein	BOQ65_RS16860
	HD domain-containing protein	BOQ65_RS16865
	Aldo/keto reductase	BOQ65_RS16870
	DUF2785 domain-containing protein	BOQ65_RS16880
	Phage portal protein	BOQ65_RS16890
	HAD family hydrolase	BOQ65_RS16895
	D-aminoacyl-tRNA deacylase	BOQ65_RS16900
	Hypothetical protein	BOQ65_RS16910
	Bifunctional (p)ppGpp synthetase/guanosine-3,5-bis(diphosphate) 3-pyrophosphohydrolase	BOQ65_RS16915
	Amidohydrolase/deacetylase family metallohydrolase	BOQ65_RS16920
	LemA family protein	BOQ65_RS16925
	30S ribosomal protein S12	BOQ65_RS16930
	GNAT family N-acetyltransferase	BOQ65_RS16935
	LPXTG cell wall anchor domain-containing protein	BOQ65_RS16945
	Hypothetical protein	BOQ65_RS16965
	Class II fumarate hydratase	BOQ65_RS16970
	Site-specific integrase	BOQ65_RS16975
	Glycosyltransferase family 2 protein	BOQ65_RS16980
	Hypothetical protein	BOQ65_RS16985
	Hypothetical protein	BOQ65_RS16995
	PTS sugar transporter subunit IIA	BOQ65_RS17000
	DNA translocase FtsK	BOQ65_RS17010
	FeoB-associated Cys-rich membrane protein	BOQ65_RS17015
	GTPase Era	BOQ65_RS17035
	Class 1b ribonucleoside-diphosphate reductase subunit alpha	BOQ65_RS17095
	YdeI/OmpD-associated family protein	BOQ65_RS17100
	Phage integrase N-terminal SAM-like domain-containing protein	BOQ65_RS17125
	LysM domain-containing protein	BOQ65_RS17140
	OsmC family protein	BOQ65_RS17160
	Acetate kinase	BOQ65_RS17175
	C1 family peptidase	BOQ65_RS17240
	Hypothetical protein	BOQ65_RS17260
	Peptide-methionine (S)-S-oxide reductase MsrA	BOQ65_RS17290
DSR_M2	Metalloregulator ArsR/SmtB family transcription factor	CFN49_RS00020
	Class II fructose-1,6-bisphosphate aldolase	CFN49_RS00025

Strain	Product	Gene Locus
	PTS fructose-like transporter subunit IIBC	CFN49_RS00030
	Sugar phosphate isomerase/epimerase	CFN49_RS00035
	DUF4428 domain-containing protein	CFN49_RS00045
	PTS sugar transporter subunit IIC	CFN49_RS00055
	PTS sugar transporter subunit IIB	CFN49_RS00060
	Phosphotransferase system, mannose/fructose-specific component IIA	CFN49_RS00070
	NFACT RNA binding domain-containing protein	CFN49_RS00145
	Winged helix-turn-helix transcriptional regulator	CFN49_RS00150
	Transketolase	CFN49_RS00235
	Phage baseplate upper protein	CFN49_RS00240
	MurR/RpiR family transcriptional regulator	CFN49_RS00260
	Hypothetical protein	CFN49_RS00265
	Sugar O-acetyltransferase	CFN49_RS00285
	IS3 family transposase	CFN49_RS00290
	Rrf2 family transcriptional regulator	CFN49_RS00360
	Nitronate monooxygenase	CFN49_RS00365
	ABC transporter ATP-binding protein	CFN49_RS00370
	Amino acid permease	CFN49_RS01995
	DUF3800 domain-containing protein	CFN49_RS02145
	Hypothetical protein	CFN49_RS02165
	Y-family DNA polymerase	CFN49_RS02170
	Hypothetical protein	CFN49_RS04340
	DUF4422 domain-containing protein	CFN49_RS05145
	LysR family transcriptional regulator	CFN49_RS05150
	Aldo/keto reductase	CFN49_RS05155
	Aldo/keto reductase	CFN49_RS05160
	Hypothetical protein	CFN49_RS05165
	Hypothetical protein	CFN49_RS05170
	IS1182 family transposase	CFN49_RS05175
	P27 family phage terminase small subunit	CFN49_RS05180
	Folate family ECF transporter S component	CFN49_RS05185
	Membrane protein	CFN49_RS05200
	IS1182 family transposase	CFN49_RS05205
	YdcF family protein	CFN49_RS05260
	50S ribosomal protein L5	CFN49_RS07255
	Hypothetical protein	CFN49_RS07260
	ROK family protein	CFN49_RS07265
	Beta-galactosidase small subunit	CFN49_RS07960
	ISL3 family transposase	CFN49_RS08970
	Winged helix-turn-helix domain-containing protein	CFN49_RS08975
	ABC transporter substrate-binding protein	CFN49_RS08990
	Endodeoxyribonuclease	CFN49_RS09075
	Energy-coupling factor transporter transmembrane protein EcfT	CFN49_RS09095
	Glycosyltransferase	CFN49_RS10005
	GNAT family N-acetyltransferase	CFN49_RS10010
	50S ribosomal protein L29	CFN49_RS10015
	Hypothetical protein	CFN49_RS10020
	Mannose/fructose/sorbose PTS transporter subunit IIA	CFN49_RS10025
	Hypothetical protein	CFN49_RS10030
	ATP-binding cassette domain-containing protein	CFN49_RS10035
	Hypothetical protein	CFN49_RS10040
	Alpha/beta hydrolase	CFN49_RS10045
	FAD-dependent oxidoreductase	CFN49_RS10050
	Ribonuclease HI family protein	CFN49_RS10060
	EbsA family protein	CFN49_RS10065
	Formate--tetrahydrofolate ligase	CFN49_RS10070
	Signal peptidase II	CFN49_RS10075
	RluA family pseudouridine synthase	CFN49_RS10080

Strain	Product	Gene Locus
	Hypothetical protein	CFN49_RS10085
	Preprotein translocase subunit SecG	CFN49_RS10090
	Bifunctional pyr operon transcriptional regulator/uracil phosphoribosyltransferase PyrR	CFN49_RS10095
	Mannitol-1-phosphate 5-dehydrogenase	CFN49_RS10100
	Ribonuclease H	CFN49_RS10110
	Carbamoyl phosphate synthase small subunit	CFN49_RS10115
	ATP-grasp domain-containing protein	CFN49_RS10125
	Histidine phosphatase family protein	CFN49_RS10130
	ABC transporter permease/substrate-binding protein	CFN49_RS10135
	DUF1801 domain-containing protein	CFN49_RS11030
	DUF1054 domain-containing protein	CFN49_RS11070
	Hypothetical protein	CFN49_RS11075
	Dihydroxyacetone kinase subunit DhaL	CFN49_RS11710
	Hypothetical protein	CFN49_RS11715
	Hypothetical protein	CFN49_RS11720
	Helix-turn-helix transcriptional regulator	CFN49_RS11725
	Hypothetical protein	CFN49_RS11730
	Sce7725 family protein	CFN49_RS12770
	Signal peptidase I	CFN49_RS13795
	Hypothetical protein	CFN49_RS15580
	NAD(P)H-binding protein	CFN49_RS15820
	Hypothetical protein	CFN49_RS15905
IDCC3501	ABC transporter permease	D0Y51_RS02925
	Malic enzyme-like NAD(P)-binding protein	D0Y51_RS02930
	Cytochrome b5 domain-containing protein	D0Y51_RS03790
	Hypothetical protein	D0Y51_RS04945
	Cof-type HAD-IIB family hydrolase	D0Y51_RS05055
	SLC13 family permease	D0Y51_RS05075
	GNAT family N-acetyltransferase	D0Y51_RS05080
	ABC transporter ATP-binding protein/permease	D0Y51_RS05095
	Hypothetical protein	D0Y51_RS05125
	DUF5655 domain-containing protein	D0Y51_RS05135
	DNA polymerase III subunit delta	D0Y51_RS05160
	Cyclic-di-AMP receptor	D0Y51_RS05165
	Sugar phosphate isomerase/epimerase	D0Y51_RS05195
	Proline iminopeptidase-family hydrolase	D0Y51_RS07170
	Glycosyltransferase	D0Y51_RS07175
	Hypothetical protein	D0Y51_RS07235
	Dihydroxyacetone kinase subunit DhaK	D0Y51_RS07240
	MMPL family transporter	D0Y51_RS08295
	Peptide chain release factor N(5)-glutamine methyltransferase	D0Y51_RS08350
	Veg family protein	D0Y51_RS09050
	LicD family protein	D0Y51_RS10545
	Ldh family oxidoreductase	D0Y51_RS10555
	Fructokinase	D0Y51_RS10560
	Nucleoside 2-deoxyribosyltransferase	D0Y51_RS10565
	Sugar O-acetyltransferase	D0Y51_RS10635
	FAD-dependent oxidoreductase	D0Y51_RS10640
	GNAT family N-acetyltransferase	D0Y51_RS10645
	Sugar porter family MFS transporter	D0Y51_RS10650
	TetR/AcrR family transcriptional regulator	D0Y51_RS10655
	Hypothetical protein	D0Y51_RS10660
	Aromatic compound hydratase/decarboxylase	D0Y51_RS10670
	Aldo/keto reductase	D0Y51_RS10675
	PRD domain-containing protein	D0Y51_RS11525
	Type II CRISPR RNA-guided endonuclease Cas9	D0Y51_RS14910
	DUF3284 domain-containing protein	D0Y51_RS14915

Strain	Product	Gene Locus
	Hypothetical protein	D0Y51_RS14950
	Hypothetical protein	D0Y51_RS15275
	Hypothetical protein	D0Y51_RS15280
	PTS lactose/cellobiose transporter subunit IIA	D0Y51_RS15290
	PTS sugar transporter subunit IIB	D0Y51_RS15295
	PTS transporter subunit EIIC	D0Y51_RS15300
	Hypothetical protein	D0Y51_RS15310
	Family 1 glycosylhydrolase	D0Y51_RS15315
	DUF4767 domain-containing protein	D0Y51_RS15330
	Mevalonate kinase	D0Y51_RS15335
	Hypothetical protein	D0Y51_RS15365
	Type IV secretory system conjugative DNA transfer family protein	D0Y51_RS15370
	Hypothetical protein	D0Y51_RS15400
	Hypothetical protein	D0Y51_RS15405
	DUF1836 domain-containing protein	D0Y51_RS15410
	Hypothetical protein	D0Y51_RS15415
	Hypothetical protein	D0Y51_RS15420
	DUF3658 domain-containing protein	D0Y51_RS15425
	FAD-dependent oxidoreductase	D0Y51_RS15430
	Large-conductance mechanosensitive channel protein MscL	D0Y51_RS15435
	Hypothetical protein	D0Y51_RS15465
	Ribosomal protein S18-alanine N-acetyltransferase	D0Y51_RS15745
	ABC transporter ATP-binding protein	D0Y51_RS15760
KC28	BspA family leucine-rich repeat surface protein	C4O30_RS00005
	Bacterial Ig-like domain-containing protein	C4O30_RS00010
	Two-component system activity regulator YycH	C4O30_RS00015
	23S rRNA (pseudouridine(1915)-N(3))-methyltransferase RlmH	C4O30_RS00020
	Glycoside hydrolase family 1 protein	C4O30_RS00025
	ROK family protein	C4O30_RS00030
	Substrate-binding domain-containing protein	C4O30_RS00035
	Cell surface protein	C4O30_RS00040
	TetR/AcrR family transcriptional regulator	C4O30_RS00050
	Cof-type HAD-IIB family hydrolase	C4O30_RS00055
	Transketolase	C4O30_RS00060
	Glycosyltransferase	C4O30_RS00080
	L-serine ammonia-lyase, iron-sulfur-dependent, subunit alpha	C4O30_RS00085
	50S ribosomal protein L18	C4O30_RS00090
	Helix-turn-helix domain-containing protein	C4O30_RS00095
	Hypothetical protein	C4O30_RS00115
	Hypothetical protein	C4O30_RS00120
	Coniferyl aldehyde dehydrogenase	C4O30_RS00125
	HK97 gp10 family phage protein	C4O30_RS00135
	Phage head closure protein	C4O30_RS00140
	DUF1801 domain-containing protein	C4O30_RS00145
	DMT family transporter	C4O30_RS00150
	YbgA family protein	C4O30_RS00155
	Hypothetical protein	C4O30_RS00160
	Hypothetical protein	C4O30_RS00165
	Nitroreductase family protein	C4O30_RS00175
	Nucleoside hydrolase	C4O30_RS00180
	Polyphosphate kinase 2 family protein	C4O30_RS00185
	Threonine synthase	C4O30_RS00190
	Peroxide stress protein YaaA	C4O30_RS00255
	Peptide ABC transporter substrate-binding protein	C4O30_RS00265
	Imidazoleglycerol-phosphate dehydratase HisB	C4O30_RS00270
	GNAT family N-acetyltransferase	C4O30_RS00280
	Cof-type HAD-IIB family hydrolase	C4O30_RS00285
	Beta-glucoside-specific PTS transporter subunit IIABC	C4O30_RS00290

Strain	Product	Gene Locus
	DUF4041 domain-containing protein	C4O30_RS00295
	MFS transporter	C4O30_RS00300
	DUF916 and DUF3324 domain-containing protein	C4O30_RS00305
	30S ribosomal protein S6	C4O30_RS00310
	Hypothetical protein	C4O30_RS00315
	MFS transporter	C4O30_RS00320
	Cation-transporting P-type ATPase	C4O30_RS00325
	Hypothetical protein	C4O30_RS00330
	Hypothetical protein	C4O30_RS00335
	Hypothetical protein	C4O30_RS00340
	Hypothetical protein	C4O30_RS00345
	Hypothetical protein	C4O30_RS00350
	Two-component system regulatory protein YycI	C4O30_RS00355
	Uracil-DNA glycosylase family protein	C4O30_RS00360
	Two-peptide bacteriocin plantaricin JK subunit PlnK	C4O30_RS00365
	Two-peptide bacteriocin plantaricin JK subunit PlnJ	C4O30_RS00370
	Chromosomal replication initiator protein DnaA	C4O30_RS00375
	Bacteriocin immunity protein	C4O30_RS00380
	Bacteriocin	C4O30_RS00385
	DegV family protein	C4O30_RS00390
	Metal ABC transporter permease	C4O30_RS00395
	Site-specific integrase	C4O30_RS00405
	Glycosyltransferase family 2 protein	C4O30_RS00410
	Phosphatase PAP2 family protein	C4O30_RS00415
	GntR family transcriptional regulator	C4O30_RS02565
	Helix-turn-helix domain-containing protein	C4O30_RS02570
	Ketopantoate reductase family protein	C4O30_RS02575
	ATP-dependent sacrificial sulfur transferase LarE	C4O30_RS02580
	Lysis protein	C4O30_RS02590
	Putative holin-like toxin	C4O30_RS02600
	AraC family transcriptional regulator	C4O30_RS04565
	Alpha-galactosidase	C4O30_RS04610
	CPBP family intramembrane metalloprotease	C4O30_RS04615
	ATP-binding protein	C4O30_RS04620
	Replisome organizer	C4O30_RS04625
	Hypothetical protein	C4O30_RS04630
	Hypothetical protein	C4O30_RS04635
	Hypothetical protein	C4O30_RS04640
	1-(5-phosphoribosyl)-5-[(5-phosphoribosylamino)methylideneamino]imidazole-4-carboxamide isomerase	C4O30_RS04645
	Hypothetical protein	C4O30_RS04660
	Helix-turn-helix domain-containing protein	C4O30_RS04665
	Glycerol-3-phosphate cytidylyltransferase	C4O30_RS04670
	SH3 domain-containing protein	C4O30_RS04675
	Hypothetical protein	C4O30_RS04680
	2-aminoethylphosphonate--pyruvate transaminase	C4O30_RS04700
	SPJ_0845 family protein	C4O30_RS04705
	Cold-shock protein	C4O30_RS04715
	PRD domain-containing protein	C4O30_RS04720
	PspC domain-containing protein	C4O30_RS04725
	Glutamine--fructose-6-phosphate transaminase (isomerizing)	C4O30_RS04735
	Alpha/beta hydrolase	C4O30_RS04740
	Flavodoxin domain-containing protein	C4O30_RS04745
	LTA synthase family protein	C4O30_RS04750
	LysR family transcriptional regulator	C4O30_RS04755
	Amino acid ABC transporter permease	C4O30_RS04760
	GNAT family N-acetyltransferase	C4O30_RS04765

Strain	Product	Gene Locus
	Hypothetical protein	C4O30_RS04770
	DASS family sodium-coupled anion symporter	C4O30_RS04775
	Beta-phosphoglucomutase	C4O30_RS04780
	ABC transporter ATP-binding protein	C4O30_RS04785
	DNA polymerase III subunit beta	C4O30_RS04790
	SpaA isopeptide-forming pilin-related protein	C4O30_RS04795
	Hypothetical protein	C4O30_RS04800
	3-deoxy-7-phosphoheptulonate synthase	C4O30_RS04805
	GNAT family N-acetyltransferase	C4O30_RS04810
	Hypothetical protein	C4O30_RS04815
	VanZ family protein	C4O30_RS04820
	Histidinol dehydrogenase	C4O30_RS04825
	Imidazole glycerol phosphate synthase subunit HisF	C4O30_RS04830
	Metal-sensitive transcriptional regulator	C4O30_RS04835
	Type II-A CRISPR-associated protein Csn2	C4O30_RS04840
	Bacterial Ig-like domain-containing protein	C4O30_RS05475
	Fructose PTS transporter subunit IIA	C4O30_RS05480
	30S ribosomal protein S4	C4O30_RS05505
	MFS transporter	C4O30_RS05560
	Sugar transferase	C4O30_RS05570
	CAP domain-containing protein	C4O30_RS05595
	DMT family transporter	C4O30_RS05605
	DUF6198 family protein	C4O30_RS05610
	ABC transporter substrate-binding protein	C4O30_RS05615
	Glycoside hydrolase family 13 protein	C4O30_RS05620
	Hypothetical protein	C4O30_RS05625
	Glycerol kinase GlpK	C4O30_RS05635
	Gluconate:H+ symporter	C4O30_RS08140
	LBP_cg2779 family protein	C4O30_RS08145
	DNA-directed RNA polymerase subunit beta	C4O30_RS08155
	Oleate hydratase	C4O30_RS08275
	Hypothetical protein	C4O30_RS08290
	Hypothetical protein	C4O30_RS08295
	ABC transporter ATP-binding protein	C4O30_RS08330
	Pentapeptide repeat-containing protein	C4O30_RS08345
	Cof-type HAD-IIB family hydrolase	C4O30_RS08350
	Twin-arginine translocation pathway signal protein	C4O30_RS08395
	Hypothetical protein	C4O30_RS08420
	Restriction endonuclease subunit S	C4O30_RS08435
	Zinc-binding dehydrogenase	C4O30_RS08440
	Crp/Fnr family transcriptional regulator	C4O30_RS08445
	Hypothetical protein	C4O30_RS09860
	Amino acid permease	C4O30_RS11205
	Hypothetical protein	C4O30_RS11210
	Hypothetical protein	C4O30_RS11215
	Phosphonate ABC transporter, permease protein PhnE	C4O30_RS11230
	Hypothetical protein	C4O30_RS11235
	Hypothetical protein	C4O30_RS11240
	Branched-chain amino acid aminotransferase	C4O30_RS11455
	DUF4428 domain-containing protein	C4O30_RS13215
	Hypothetical protein	C4O30_RS13220
	AEC family transporter	C4O30_RS14970
	NAD(P)H-binding protein	C4O30_RS14985
	Alpha/beta hydrolase-fold protein	C4O30_RS15045
	Phosphoketolase family protein	C4O30_RS15050
	Asparagine--tRNA ligase	C4O30_RS15055
	IS256 family transposase	C4O30_RS15060
	50S ribosomal protein L16	C4O30_RS15975

Strain	Product	Gene Locus
	Amidohydrolase family protein	C4O30_RS15980
	Hypothetical protein	C4O30_RS16000
	Iron ABC transporter permease	C4O30_RS16005
	Flagellar biosynthetic protein FlhB	C4O30_RS16010
	Phosphate ABC transporter ATP-binding protein PstB	C4O30_RS16015
	Hypothetical protein	C4O30_RS16020
	Phage tail protein	C4O30_RS16025
	ATP-grasp domain-containing protein	C4O30_RS16030
	LPXTG cell wall anchor domain-containing protein	C4O30_RS16035
	YdhK family protein	C4O30_RS16040
	Phage tail family protein	C4O30_RS16045
	Peptidoglycan DD-metalloendopeptidase family protein	C4O30_RS16050
	LysR family transcriptional regulator	C4O30_RS16055
	MarR family transcriptional regulator	C4O30_RS16060
	MFS transporter	C4O30_RS16065
	Stealth conserved region 3 domain-containing protein	C4O30_RS16070
	Phosphonoacetaldehyde hydrolase	C4O30_RS16075
	SDR family NAD(P)-dependent oxidoreductase	C4O30_RS16080
	Pyruvate oxidase	C4O30_RS16085
	Hypothetical protein	C4O30_RS16090
	Stealth CR1 domain-containing protein	C4O30_RS16095
	Sugar transferase	C4O30_RS16100
	Type II toxin-antitoxin system RelB/DinJ family antitoxin	C4O30_RS16105
	Glycosyltransferase family 2 protein	C4O30_RS16110
	Glycosyltransferase family 2 protein	C4O30_RS16115
	Transposase	C4O30_RS16120
	UDP-galactopyranose mutase	C4O30_RS16140
	Lp_1303a family serine-rich repeat glycoprotein adhesin	C4O30_RS16150
	Hypothetical protein	C4O30_RS16155
	Hypothetical protein	C4O30_RS16160
	Hypothetical protein	C4O30_RS16165
	Polysaccharide biosynthesis protein	C4O30_RS16170
	Imidazole glycerol phosphate synthase subunit HisH	C4O30_RS16195
	Aspartate--ammonia ligase	C4O30_RS16200
	FMN-dependent NADH-azoreductase	C4O30_RS16205
	DUF4422 domain-containing protein	C4O30_RS16210
	Helix-turn-helix transcriptional regulator	C4O30_RS16215
LMT1-48	Hypothetical protein	EGU28_RS00010
	Methylated-DNA--[protein]-cysteine S-methyltransferase	EGU28_RS00030
	Lytic polysaccharide monooxygenase	EGU28_RS00085
	Replicative DNA helicase	EGU28_RS00090
	Helix-turn-helix domain-containing protein	EGU28_RS00140
	SDR family oxidoreductase	EGU28_RS00145
	DUF2207 domain-containing protein	EGU28_RS00150
	Hypothetical protein	EGU28_RS00165
	Type I restriction endonuclease subunit R	EGU28_RS00170
	Glycosyltransferase	EGU28_RS00180
	Hypothetical protein	EGU28_RS00240
	Phospho-sugar mutase	EGU28_RS00250
	Iron-containing alcohol dehydrogenase	EGU28_RS00255
	DNA starvation/stationary phase protection protein	EGU28_RS00275
	TetR/AcrR family transcriptional regulator	EGU28_RS00875
	Hypothetical protein	EGU28_RS00880
	GNAT family N-acetyltransferase	EGU28_RS00910
	DUF916 and DUF3324 domain-containing protein	EGU28_RS00940
	ABC transporter substrate-binding protein	EGU28_RS00945
	Amidohydrolase family protein	EGU28_RS00960
	Hypothetical protein	EGU28_RS00980

Strain	Product	Gene Locus
	Matrixin family metalloprotease	EGU28_RS00985
	MFS transporter	EGU28_RS00990
	MFS transporter	EGU28_RS01005
	ABC transporter permease subunit	EGU28_RS01010
	NAD(P)H-dependent oxidoreductase	EGU28_RS01015
	Hypothetical protein	EGU28_RS01020
	Sugar ABC transporter permease	EGU28_RS01030
	Glycogen/starch/alpha-glucan phosphorylase	EGU28_RS01035
	S4 domain-containing protein YaaA	EGU28_RS01050
	DNA topoisomerase (ATP-hydrolyzing) subunit B	EGU28_RS01055
	Cys-tRNA(Pro) deacylase	EGU28_RS01060
	Hypothetical protein	EGU28_RS01065
	Arsenate reductase (thioredoxin)	EGU28_RS01070
	GntR family transcriptional regulator	EGU28_RS01075
	TMEM175 family protein	EGU28_RS01080
	Hypothetical protein	EGU28_RS01085
	Cell wall metabolism sensor histidine kinase WalK	EGU28_RS01090
	Zinc ribbon domain-containing protein	EGU28_RS02725
	Transporter substrate-binding domain-containing protein	EGU28_RS04000
	TfoX/Sxy family protein	EGU28_RS05620
	Nuclear transport factor 2 family protein	EGU28_RS05655
	DHA2 family efflux MFS transporter permease subunit	EGU28_RS05735
	Uracil phosphoribosyltransferase	EGU28_RS05740
	DUF916 and DUF3324 domain-containing protein	EGU28_RS05755
	50S ribosomal protein L34	EGU28_RS05770
	LCP family protein	EGU28_RS05835
	Hypothetical protein	EGU28_RS05850
	N-acetylneuraminate lyase	EGU28_RS05855
	LVIS_2131 family protein	EGU28_RS05860
	Alpha/beta hydrolase	EGU28_RS05895
	HIRAN domain-containing protein	EGU28_RS05900
	Phage terminase small subunit P27 family	EGU28_RS05930
	Phage tail tape measure protein	EGU28_RS05935
	Phage tail tape measure protein	EGU28_RS05945
	Phage tail tape measure protein	EGU28_RS05950
	Hypothetical protein	EGU28_RS05955
	Phage tail spike protein	EGU28_RS05960
	Phage tail protein	EGU28_RS05965
	Phage tail protein	EGU28_RS05970
	Phage tail protein	EGU28_RS05975
	Universal stress protein	EGU28_RS05980
	Hypothetical protein	EGU28_RS05985
	Phage tail family protein	EGU28_RS05990
	Phage tail family protein	EGU28_RS05995
	GntR family transcriptional regulator	EGU28_RS06000
	Phage tail protein	EGU28_RS06015
	Phage portal protein	EGU28_RS06020
	LysR family transcriptional regulator	EGU28_RS06105
	WxL domain-containing protein	EGU28_RS06615
	PLP-dependent aminotransferase family protein	EGU28_RS07070
	IMP dehydrogenase	EGU28_RS07805
	FAD-dependent oxidoreductase	EGU28_RS08285
	DTMP kinase	EGU28_RS08650
	Hypothetical protein	EGU28_RS08665
	Hypothetical protein	EGU28_RS08670
	Hypothetical protein	EGU28_RS08675
	Hypothetical protein	EGU28_RS08680
	Hypothetical protein	EGU28_RS08695

Strain	Product	Gene Locus
	Hypothetical protein	EGU28_RS08700
	DUF3781 domain-containing protein	EGU28_RS09790
	4-(cytidine 5-diphospho)-2-C-methyl-D-erythritol kinase	EGU28_RS10800
	RNase adapter RapZ	EGU28_RS10825
	Zinc-binding dehydrogenase	EGU28_RS10975
	Putative holin-like toxin	EGU28_RS11110
	Hypothetical protein	EGU28_RS11270
	Hypothetical protein	EGU28_RS11295
	Carbon-nitrogen family hydrolase	EGU28_RS11300
	ImmA/IrrE family metallo-endopeptidase	EGU28_RS11305
	Hypothetical protein	EGU28_RS11310
	TetR/AcrR family transcriptional regulator	EGU28_RS11355
	Transporter substrate-binding domain-containing protein	EGU28_RS11590
	LysR family transcriptional regulator	EGU28_RS12235
	Hypothetical protein	EGU28_RS12915
	Bacterial Ig-like domain-containing protein	EGU28_RS12930
	Cellulase family glycosylhydrolase	EGU28_RS12945
	ABC transporter ATP-binding protein	EGU28_RS12950
	Phage tail family protein	EGU28_RS12960
	TRNA (adenosine(37)-N6)-threonylcarbamoyltransferase complex dimerization subunit type 1 TsaB	EGU28_RS12965
	DUF1617 family protein	EGU28_RS12985
	Hypothetical protein	EGU28_RS13030
	Phosphoribosyl-ATP diphosphatase	EGU28_RS13045
	16S rRNA (guanine(527)-N(7))-methyltransferase RsmG	EGU28_RS13050
	Nucleoside transporter C-terminal domain-containing protein	EGU28_RS13055
	LytTR family transcriptional regulator	EGU28_RS13095
	Phosphopyruvate hydratase	EGU28_RS13150
	DUF1129 domain-containing protein	EGU28_RS13635
	Hypothetical protein	EGU28_RS15440
	DUF806 family protein	EGU28_RS15450
	Hypothetical protein	EGU28_RS15490
	LacI family DNA-binding transcriptional regulator	EGU28_RS15495
	Citrate lyase holo-[acyl-carrier protein] synthase	EGU28_RS15510
	Carbohydrate ABC transporter permease	EGU28_RS15525
	PepSY domain-containing protein	EGU28_RS15545
	Hydantoinase/oxoprolinase family protein	EGU28_RS15550
	Hypothetical protein	EGU28_RS15555
	Tryptophan--tRNA ligase	EGU28_RS15560
	ImmA/IrrE family metallo-endopeptidase	EGU28_RS15565
	LysR family transcriptional regulator	EGU28_RS15570
	Iron-sulfur cluster biosynthesis family protein	EGU28_RS15575
	Alpha/beta hydrolase	EGU28_RS15585
	Helix-turn-helix domain-containing protein	EGU28_RS15590
	GMP reductase	EGU28_RS15595
	Sulfite exporter TauE/SafE family protein	EGU28_RS15600
	Phosphoribosylformylglycinamidine cyclo-ligase	EGU28_RS15615
	Hypothetical protein	EGU28_RS15620
	Hypothetical protein	EGU28_RS15625
	VanZ family protein	EGU28_RS15630
	Hypothetical protein	EGU28_RS15635
	GyrI-like domain-containing protein	EGU28_RS15640
	50S ribosomal protein L15	EGU28_RS15645
	Hypothetical protein	EGU28_RS15650
	Hypothetical protein	EGU28_RS15660
	Helix-turn-helix transcriptional regulator	EGU28_RS15665
	Copper-translocating P-type ATPase	EGU28_RS15670
	ATP-binding protein	EGU28_RS15680

Strain	Product	Gene Locus
	Putative hydroxymethylpyrimidine transporter CytX	EGU28_RS15690
	Hypothetical protein	EGU28_RS15700
	DUF916 and DUF3324 domain-containing protein	EGU28_RS15705
	PTS mannitol-specific transporter subunit IIBC	EGU28_RS15710
	Hypothetical protein	EGU28_RS15725
	LysR family transcriptional regulator	EGU28_RS15730
	Histidine kinase	EGU28_RS15745
	Hypothetical protein	EGU28_RS15775
	Zinc-binding alcohol dehydrogenase family protein	EGU28_RS15795
	GlsB/YeaQ/YmgE family stress response membrane protein	EGU28_RS15815
	PadR family transcriptional regulator	EGU28_RS15825
	YbgA family protein	EGU28_RS15835
	DUF2252 domain-containing protein	EGU28_RS15845
	Vitamin B12 independent methionine synthase	EGU28_RS15850
	Phage tail protein	EGU28_RS15855
	Phosphatase PAP2 family protein	EGU28_RS15860
	DhaKLM operon coactivator DhaQ	EGU28_RS15865
	Amino acid permease	EGU28_RS15870
	Amidohydrolase family protein	EGU28_RS15875
	Alpha/beta fold hydrolase	EGU28_RS15880
	AraC family transcriptional regulator	EGU28_RS15895
	Hypothetical protein	EGU28_RS15900
	Biotin-dependent carboxyltransferase	EGU28_RS15910
	MraY family glycosyltransferase	EGU28_RS15925
	Bifunctional pyr operon transcriptional regulator/uracil phosphoribosyltransferase PyrR	EGU28_RS15930
	Nucleobase:cation symporter-2 family protein	EGU28_RS15935
	50S ribosomal protein L23	EGU28_RS15940
	NCS2 family permease	EGU28_RS15945
	Cation:proton antiporter	EGU28_RS15950
	NmrA family NAD(P)-binding protein	EGU28_RS15955
	WYL domain-containing protein	EGU28_RS15960
	30S ribosomal protein S19	EGU28_RS15965
	MobQ family relaxase	EGU28_RS15970
	Metalloregulator ArsR/SmtB family transcription factor	EGU28_RS15975
	EAL domain-containing protein	EGU28_RS15980
	Hypothetical protein	EGU28_RS15985
	Hypothetical protein	EGU28_RS15990
	Glycosyltransferase	EGU28_RS15995
	PTS transporter subunit IIC	EGU28_RS16000
	MerR family transcriptional regulator	EGU28_RS16025
	4-hydroxy-tetrahydrodipicolinate reductase	EGU28_RS16030
	50S ribosomal protein L3	EGU28_RS16035
	Phage scaffolding protein	EGU28_RS16040
	Phage terminase small subunit P27 family	EGU28_RS16060
	HIT family protein	EGU28_RS16065
	Alpha-galactosidase	EGU28_RS16070
	Type 1 glycerol-3-phosphate oxidase	EGU28_RS16075
	Hypothetical protein	EGU28_RS16080
	DNA-directed RNA polymerase subunit beta	EGU28_RS16135
	PAS domain-containing protein	EGU28_RS16140
	Sodium:proton antiporter	EGU28_RS16150
	Hypothetical protein	EGU28_RS16160
	Hypothetical protein	EGU28_RS16165
	PTS sugar transporter subunit IIB	EGU28_RS16180
	MarR family transcriptional regulator	EGU28_RS16200
	DUF916 and DUF3324 domain-containing protein	EGU28_RS16210
	Hypothetical protein	EGU28_RS16215

Strain	Product	Gene Locus
	Hypothetical protein	EGU28_RS16230
	Histidinol-phosphatase HisJ	EGU28_RS16335
	LPXTG cell wall anchor domain-containing protein	EGU28_RS16345
	MarR family transcriptional regulator	EGU28_RS16360
	Bacterial Ig-like domain-containing protein	EGU28_RS16370
83-18	Glucose-1-phosphate thymidylyltransferase RfbA	GPK32_RS00005
	TetR/AcrR family transcriptional regulator	GPK32_RS00010
	Alpha/beta hydrolase	GPK32_RS00015
	Helix-turn-helix domain-containing protein	GPK32_RS00020
	Capsid protein	GPK32_RS00035
	MraY family glycosyltransferase	GPK32_RS00040
	Bifunctional pyr operon transcriptional regulator/uracil phosphoribosyltransferase PyrR	GPK32_RS00045
	Nucleobase:cation symporter-2 family protein	GPK32_RS00050
	50S ribosomal protein L23	GPK32_RS00055
	NCS2 family permease	GPK32_RS00060
	Cation:proton antiporter	GPK32_RS00065
	NmrA family NAD(P)-binding protein	GPK32_RS00070
	WYL domain-containing protein	GPK32_RS00075
	30S ribosomal protein S19	GPK32_RS00080
	MobQ family relaxase	GPK32_RS00085
	Metalloregulator ArsR/SmtB family transcription factor	GPK32_RS00090
	EAL domain-containing protein	GPK32_RS00095
	Hypothetical protein	GPK32_RS00100
	Hypothetical protein	GPK32_RS00105
	Glycosyltransferase	GPK32_RS00110
	Fructose-6-phosphate aldolase	GPK32_RS00115
	PTS sugar transporter subunit IIC	GPK32_RS00120
	Orotidine-5-phosphate decarboxylase	GPK32_RS00125
	Cellulase family glycosylhydrolase	GPK32_RS00140
	DMT family transporter	GPK32_RS00145
	Thymidylate synthase	GPK32_RS00150
	Ribokinase	GPK32_RS00155
	Hypothetical protein	GPK32_RS00160
	DUF624 domain-containing protein	GPK32_RS00165
	Hypothetical protein	GPK32_RS00170
	IS3 family transposase	GPK32_RS00180
	Biotin transporter BioY	GPK32_RS00185
	YitT family protein	GPK32_RS00190
	Hypothetical protein	GPK32_RS00195
	Hypothetical protein	GPK32_RS00200
	DUF3284 domain-containing protein	GPK32_RS00205
	LarC family nickel insertion protein	GPK32_RS00215
	IS66 family insertion sequence element accessory protein TnpB	GPK32_RS00220
	Cell surface protein	GPK32_RS00225
	UbiD family decarboxylase	GPK32_RS00245
	Xaa-Pro peptidase family protein	GPK32_RS00250
	Catabolite control protein A	GPK32_RS00255
	PTS transporter subunit EIIC	GPK32_RS00265
	PTS transporter subunit EIIC	GPK32_RS00270
	Bifunctional DNA primase/polymerase	GPK32_RS00275
	LPXTG cell wall anchor domain-containing protein	GPK32_RS00280
	UDP-N-acetylglucosamine 1-carboxyvinyltransferase	GPK32_RS00285
	Glutamine-hydrolyzing GMP synthase	GPK32_RS00290
	Glucose-6-phosphate dehydrogenase	GPK32_RS00300
	Hypothetical protein	GPK32_RS00310
	Type II toxin-antitoxin system prevent-host-death family antitoxin	GPK32_RS00315
	Phage tail tape measure protein	GPK32_RS00320

Strain	Product	Gene Locus
	Nucleoside-diphosphate kinase	GPK32_RS00345
	SPFH domain-containing protein	GPK32_RS00350
	Polyketide cyclase	GPK32_RS00355
	Y-family DNA polymerase	GPK32_RS00360
	DeoR/GlpR family DNA-binding transcription regulator	GPK32_RS00365
	Hypothetical protein	GPK32_RS00380
	Helix-turn-helix domain-containing protein	GPK32_RS00385
	Hypothetical protein	GPK32_RS00395
	Hypothetical protein	GPK32_RS00400
	Hypothetical protein	GPK32_RS00405
	AP2 domain-containing protein	GPK32_RS00410
	Diacylglycerol kinase family protein	GPK32_RS00415
	SDR family oxidoreductase	GPK32_RS00420
	Glycoside hydrolase family 1 protein	GPK32_RS00435
	ABC transporter ATP-binding protein/permease	GPK32_RS00440
	Alkaline phosphatase family protein	GPK32_RS00445
	Hypothetical protein	GPK32_RS00470
	Nucleoid occlusion protein	GPK32_RS00485
	TIM barrel protein	GPK32_RS00495
	YebC/PmpR family DNA-binding transcriptional regulator	GPK32_RS00500
	Hypothetical protein	GPK32_RS00505
	Ribokinase	GPK32_RS00510
	Competence type IV pilus ATPase ComGA	GPK32_RS00515
	Membrane protein	GPK32_RS00520
	Type II secretion system F family protein	GPK32_RS00525
	Competence type IV pilus major pilin ComGC	GPK32_RS00530
	SNF2-related protein	GPK32_RS00535
	O-antigen ligase family protein	GPK32_RS00545
	Redoxin NrdH	GPK32_RS00550
	Type II secretion system protein	GPK32_RS00555
	Hypothetical protein	GPK32_RS00560
	Prepilin-type N-terminal cleavage/methylation domain-containing protein	GPK32_RS00565
	Class I SAM-dependent methyltransferase	GPK32_RS00570
	Histidine phosphatase family protein	GPK32_RS00575
	Metallophosphoesterase family protein	GPK32_RS00580
	Bifunctional UDP-sugar hydrolase/5-nucleotidase	GPK32_RS00585
	TIGR01457 family HAD-type hydrolase	GPK32_RS00590
	Hypothetical protein	GPK32_RS00595
	Class 1b ribonucleoside-diphosphate reductase subunit alpha	GPK32_RS00600
	ISL3 family transposase	GPK32_RS00605
	TIGR01906 family membrane protein	GPK32_RS00610
	VTT domain-containing protein	GPK32_RS00615
	Peptidylprolyl isomerase	GPK32_RS00620
	CvfD/Ygs/GSP13 family RNA-binding post-transcriptional regulator	GPK32_RS00625
	DMT family transporter	GPK32_RS00630
	Class IIb bacteriocin, lactobin A/cerein 7B family	GPK32_RS00635
	Clp protease ClpP	GPK32_RS00640
	CRISPR-associated endonuclease Cas2	GPK32_RS00645
	Cytosine permease	GPK32_RS00650
	DNA cytosine methyltransferase	GPK32_RS00670
	DNA polymerase III subunit alpha	GPK32_RS00675
	RIP metalloprotease RseP	GPK32_RS00680
	30S ribosomal protein S10	GPK32_RS00685
	DNA alkylation repair protein	GPK32_RS00690
	Helix-turn-helix domain containing protein	GPK32_RS00695
	Potassium-transporting ATPase subunit KdpB	GPK32_RS00700
	Hypothetical protein	GPK32_RS00730
	Choloylglycine hydrolase family protein	GPK32_RS00735

Strain	Product	Gene Locus
	Hypothetical protein	GPK32_RS00745
	Hypothetical protein	GPK32_RS00750
	Phage tail spike protein	GPK32_RS00755
	Phage tail family protein	GPK32_RS00760
	GNAT family N-acetyltransferase	GPK32_RS00770
	HAMP domain-containing sensor histidine kinase	GPK32_RS00785
	Hypothetical protein	GPK32_RS00790
	Helix-turn-helix domain-containing protein	GPK32_RS00795
	Phage major capsid protein	GPK32_RS00800
	Ribonuclease Z	GPK32_RS00815
	TetR/AcrR family transcriptional regulator	GPK32_RS00820
	GNAT family protein	GPK32_RS00870
	FAD-dependent oxidoreductase	GPK32_RS00880
	DHA2 family efflux MFS transporter permease subunit	GPK32_RS00885
	TrsD/TraD family conjugative transfer protein	GPK32_RS00890
	MarR family transcriptional regulator	GPK32_RS00925
	ABC-2 transporter permease	GPK32_RS00945
	Cell surface protein	GPK32_RS00950
	Hypothetical protein	GPK32_RS00955
	Hypothetical protein	GPK32_RS00965
	Hypothetical protein	GPK32_RS00970
	Hypothetical protein	GPK32_RS00975
	Hypothetical protein	GPK32_RS00980
	Helix-turn-helix domain-containing protein	GPK32_RS00985
	PTS sugar transporter subunit IIA	GPK32_RS00990
	Lipoate--protein ligase family protein	GPK32_RS00995
	ATP-binding protein	GPK32_RS01000
	2,3-diphosphoglycerate-dependent phosphoglycerate mutase	GPK32_RS01030
	FAD-dependent oxidoreductase	GPK32_RS01040
	Terminase large subunit	GPK32_RS01050
	ParA family protein	GPK32_RS01055
	Lactate oxidase	GPK32_RS01060
	Alpha-mannosidase	GPK32_RS01065
	Hypothetical protein	GPK32_RS01070
	CidA/LrgA family protein	GPK32_RS01075
	SGNH/GDSL hydrolase family protein	GPK32_RS01090
	Transcriptional regulator GutM	GPK32_RS01095
	Metallophosphoesterase	GPK32_RS01110
	Hypothetical protein	GPK32_RS01115
	M15 family metallopeptidase	GPK32_RS01120
	Biotin/lipoyl-containing protein	GPK32_RS01125
	Citrate lyase holo-[acyl-carrier protein] synthase	GPK32_RS01130
	PTS transporter subunit IIC	GPK32_RS01145
	Zinc-ribbon domain-containing protein	GPK32_RS01155
	PTS system mannose/fructose/sorbose family transporter subunit IID	GPK32_RS01160
	Zinc-binding dehydrogenase	GPK32_RS01165
	DUF916 and DUF3324 domain-containing protein	GPK32_RS01170
	Hypothetical protein	GPK32_RS01180
	Histidine phosphatase family protein	GPK32_RS01185
	M20/M25/M40 family metallo-hydrolase	GPK32_RS01190
	Hypothetical protein	GPK32_RS01205
	Hypothetical protein	GPK32_RS01210
	Copper-translocating P-type ATPase	GPK32_RS01230
	Cupredoxin domain-containing protein	GPK32_RS01235
	LrgB family protein	GPK32_RS01250
	50S ribosomal protein L36	GPK32_RS01255
	Gfo/Idh/MocA family oxidoreductase	GPK32_RS01260
	Amino acid ABC transporter substrate-binding protein/permease	GPK32_RS01265

Strain	Product	Gene Locus
	Metal-sulfur cluster assembly factor	GPK32_RS01270
	Zinc-binding alcohol dehydrogenase family protein	GPK32_RS01280
	Glycosyltransferase family 2 protein	GPK32_RS01285
	L,D-transpeptidase	GPK32_RS01295
	Cysteine desulfurase family protein	GPK32_RS01300
	Beta-glucoside-specific PTS transporter subunit IIABC	GPK32_RS01305
	DeoR/GlpR family DNA-binding transcription regulator	GPK32_RS01310
	Dihydroorotate dehydrogenase	GPK32_RS01315
	MFS transporter	GPK32_RS01320
	Phage tail spike protein	GPK32_RS01325
	Type II CRISPR-associated endonuclease Cas1	GPK32_RS01330
	Argininosuccinate synthase	GPK32_RS01335
	DegV family protein	GPK32_RS01345
	Helix-turn-helix domain-containing protein	GPK32_RS01350
	Hypothetical protein	GPK32_RS01375
	Head-tail connector protein	GPK32_RS01380
	Dihydrofolate reductase	GPK32_RS01385
	Recombinase family protein	GPK32_RS01390
	Phage major capsid protein	GPK32_RS01395
	Glycosyltransferase	GPK32_RS01400
	Threonine/serine exporter family protein	GPK32_RS01405
	Amino acid permease	GPK32_RS01410
	Branched-chain amino acid transport system II carrier protein	GPK32_RS01415
	PTS sugar transporter subunit IIA	GPK32_RS01420
	PTS ascorbate transporter subunit IIC	GPK32_RS01425
	Hypothetical protein	GPK32_RS01430
	DUF2785 domain-containing protein	GPK32_RS01440
	Phage portal protein	GPK32_RS01445
	N-acetylneuraminate lyase	GPK32_RS01450
	Hypothetical protein	GPK32_RS01460
	DUF1056 family protein	GPK32_RS01470
	HNH endonuclease	GPK32_RS01475
	Flavocytochrome c	GPK32_RS01480
	Phage portal protein	GPK32_RS01485
	Hypothetical protein	GPK32_RS01490
	Sugar O-acetyltransferase	GPK32_RS01505
	MFS transporter	GPK32_RS01510
	Energy-coupling factor transporter transmembrane component T	GPK32_RS01515
	UbiX family flavin prenyltransferase	GPK32_RS01520
	MerR family transcriptional regulator	GPK32_RS01525
	FAD synthetase family protein	GPK32_RS01530
	Class I SAM-dependent methyltransferase	GPK32_RS01535
	Terminase small subunit	GPK32_RS02630
	IS30-like element ISLpl1 family transposase	GPK32_RS02640
	IS3 family transposase	GPK32_RS02645
	IS30 family transposase	GPK32_RS02650
	Valine--tRNA ligase	GPK32_RS02940
	Type II toxin-antitoxin system RelB/DinJ family antitoxin	GPK32_RS02985
	Type II toxin-antitoxin system RelB/DinJ family antitoxin	GPK32_RS02990
	Type II toxin-antitoxin system YafQ family toxin	GPK32_RS02995
	Type II toxin-antitoxin system YafQ family toxin	GPK32_RS03000
	DegV family protein	GPK32_RS03185
	Replication-associated recombination protein A	GPK32_RS03240
	Hypothetical protein	GPK32_RS03250
	Glycerophosphodiester phosphodiesterase	GPK32_RS03255
	Hypothetical protein	GPK32_RS03955
	Zinc-binding dehydrogenase	GPK32_RS03960
	Restriction endonuclease subunit S	GPK32_RS03965

Strain	Product	Gene Locus
	ROK family protein	GPK32_RS03970
	DNA/RNA non-specific endonuclease	GPK32_RS03990
	DUF1831 domain-containing protein	GPK32_RS03995
	Polyphosphate kinase 2 family protein	GPK32_RS04010
	Glucose-1-phosphate thymidylyltransferase RfbA	GPK32_RS04025
	Hypothetical protein	GPK32_RS04030
	Hypothetical protein	GPK32_RS04045
	Hypothetical protein	GPK32_RS04050
	Hypothetical protein	GPK32_RS04060
	Peptide ABC transporter substrate-binding protein	GPK32_RS04065
	ISL3-like element ISP1 family transposase	GPK32_RS04070
	C1 family peptidase	GPK32_RS04075
	Hypothetical protein	GPK32_RS04080
	ABC transporter ATP-binding protein	GPK32_RS04085
	ABC transporter permease	GPK32_RS04090
	N-acetylmannosamine-6-phosphate 2-epimerase	GPK32_RS04095
	Isochorismatase family cysteine hydrolase	GPK32_RS04100
	1-deoxy-D-xylulose-5-phosphate synthase	GPK32_RS04105
	Na+/H+ antiporter	GPK32_RS04110
	Gfo/Idh/MocA family oxidoreductase	GPK32_RS04115
	Gfo/Idh/MocA family oxidoreductase	GPK32_RS04120
	MurR/RpiR family transcriptional regulator	GPK32_RS04125
	LacI family DNA-binding transcriptional regulator	GPK32_RS04130
	Cof-type HAD-IIB family hydrolase	GPK32_RS04135
	4-hydroxyphenylacetate 3-hydroxylase N-terminal domain-containing protein	GPK32_RS04140
	Hypothetical protein	GPK32_RS04145
	Hypothetical protein	GPK32_RS04150
	TRNA uridine-5-carboxymethylaminomethyl(34) synthesis enzyme MnmG	GPK32_RS04155
	Helix-turn-helix domain-containing protein	GPK32_RS04160
	Hypothetical protein	GPK32_RS04165
	Cof-type HAD-IIB family hydrolase	GPK32_RS04170
	Hypothetical protein	GPK32_RS04175
	DUF2316 family protein	GPK32_RS04190
	Hypothetical protein	GPK32_RS04195
	Site-specific integrase	GPK32_RS04200
	GNVR domain-containing protein	GPK32_RS04205
	Capsid protein	GPK32_RS04210
	Zinc metallopeptidase	GPK32_RS04570
	DMT family transporter	GPK32_RS04715
	Matrixin family metalloprotease	GPK32_RS04930
	D-alanine--D-alanine ligase family protein	GPK32_RS05235
	Hypothetical protein	GPK32_RS05330
	LysM domain-containing protein	GPK32_RS05350
	Hypothetical protein	GPK32_RS06050
	Prolipoprotein diacylglyceryl transferase	GPK32_RS06480
	Hypothetical protein	GPK32_RS07250
	DUF308 domain-containing protein	GPK32_RS07355
	UTRA domain-containing protein	GPK32_RS07500
	Hypothetical protein	GPK32_RS08315
	ROK family protein	GPK32_RS08320
	YaaL family protein	GPK32_RS09785
	Thiol peroxidase	GPK32_RS09790
	Glycerol-3-phosphate cytidylyltransferase	GPK32_RS09795
	Hypothetical protein	GPK32_RS09800
	Metallophosphoesterase family protein	GPK32_RS09805
	Glutamate--cysteine ligase	GPK32_RS09880
	TRNA uracil 4-sulfurtransferase ThiI	GPK32_RS09895

Strain	Product	Gene Locus
	CvpA family protein	GPK32_RS10035
	2-amino-4-hydroxy-6- hydroxymethyldihydropteridine diphosphokinase	GPK32_RS10840
	GNAT family N-acetyltransferase	GPK32_RS11415
	5-methylthioadenosine/adenosylhomocysteine nucleosidase	GPK32_RS11420
	Hypothetical protein	GPK32_RS11455
	Hypothetical protein	GPK32_RS11505
	UDP-N-acetylmuramoyl-L-alanyl-D-glutamate--2, 6-diaminopimelate ligase	GPK32_RS11510
	YvcK family protein	GPK32_RS11525
	Histidine phosphatase family protein	GPK32_RS11555
	MarR family winged helix-turn-helix transcriptional regulator	GPK32_RS11580
	Phage terminase small subunit P27 family	GPK32_RS11585
	Phage tail tape measure protein	GPK32_RS11595
	Phage tail tape measure protein	GPK32_RS11600
	Hypothetical protein	GPK32_RS11605
	Phage tail spike protein	GPK32_RS11610
	Phage tail protein	GPK32_RS11620
	Phage tail protein	GPK32_RS11630
	TRNA 2-thiouridine(34) synthase MnmA	GPK32_RS11635
	Exosortase family protein XrtG	GPK32_RS11640
	Phage tail family protein	GPK32_RS11645
	Phage tail family protein	GPK32_RS11650
	Holin	GPK32_RS11665
	Phage portal protein	GPK32_RS11670
	Firmicu-CTERM sorting domain-containing protein	GPK32_RS11720
	Hypothetical protein	GPK32_RS11735
	Oligoendopeptidase F	GPK32_RS11740
	Transketolase	GPK32_RS11745
	Lp_1303a family serine-rich repeat glycoprotein adhesin	GPK32_RS11850
	YutD family protein	GPK32_RS11855
	Bifunctional glutamate--cysteine ligase GshA/glutathione synthetase GshB	GPK32_RS11860
	F0F1 ATP synthase subunit epsilon	GPK32_RS11865
	Prophage P1 protein 59	GPK32_RS11870
	APC family permease	GPK32_RS11880
	Hypothetical protein	GPK32_RS11915
	Hypothetical protein	GPK32_RS11940
	Hypothetical protein	GPK32_RS11950
	2-hydroxyacid dehydrogenase family protein	GPK32_RS12070
	Alpha-rhamnosidase	GPK32_RS12230
	Metal ABC transporter substrate-binding protein	GPK32_RS12235
	Nickel-dependent lactate racemase	GPK32_RS12240
	Central glycolytic genes regulator	GPK32_RS12480
	Dihydroneopterin aldolase	GPK32_RS12745
	MetQ/NlpA family ABC transporter substrate-binding protein	GPK32_RS12905
	Phosphotransferase	GPK32_RS13365
	HD domain-containing protein	GPK32_RS13370
	Methionine ABC transporter permease	GPK32_RS13465
	CopY/TcrY family copper transport repressor	GPK32_RS13810
	MarR family transcriptional regulator	GPK32_RS13815
	LacI family DNA-binding transcriptional regulator	GPK32_RS14285
	Transposase	GPK32_RS14295
	Acyltransferase	GPK32_RS14310
	DUF916 and DUF3324 domain-containing protein	GPK32_RS14335
	Hypothetical protein	GPK32_RS14345
	Hypothetical protein	GPK32_RS14350
	Hypothetical protein	GPK32_RS14355
	Glycoside hydrolase family 2 TIM barrel-domain containing protein	GPK32_RS14360
	Heavy metal translocating P-type ATPase	GPK32_RS14365
	DNA cytosine methyltransferase	GPK32_RS14370

Strain	Product	Gene Locus
	Alpha-glucosidase	GPK32_RS14375
	Hypothetical protein	GPK32_RS14380
	Hypothetical protein	GPK32_RS14405
	Transcriptional regulator	GPK32_RS14415
	Site-specific integrase	GPK32_RS14420
	LPXTG cell wall anchor domain-containing protein	GPK32_RS14425
	GRP family sugar transporter	GPK32_RS14430
	Hypothetical protein	GPK32_RS14435
	Hypothetical protein	GPK32_RS14440
	VTT domain-containing protein	GPK32_RS14450
	Glycosyltransferase family 2 protein	GPK32_RS14455
	M3 family oligoendopeptidase	GPK32_RS14460
	MFS transporter	GPK32_RS14465
	PD-(D/E)XK nuclease family protein	GPK32_RS14470
	Zinc ribbon domain-containing protein	GPK32_RS14505
	Ribosome biogenesis GTPase Der	GPK32_RS14535
	Hypothetical protein	GPK32_RS14895
	Hypothetical protein	GPK32_RS14900
	Carbamoyl-phosphate synthase large subunit	GPK32_RS15300
	Fructose-6-phosphate aldolase	GPK32_RS15335
	NlpC/P60 family protein	GPK32_RS15340
	Alpha-glucosidase	GPK32_RS15685
	ABC transporter permease	GPK32_RS15990
	Spx/MgsR family RNA polymerase-binding regulatory protein	GPK32_RS15995
	DUF669 domain-containing protein	GPK32_RS16000
	Sugar ABC transporter permease	GPK32_RS16005
	Sugar ABC transporter permease	GPK32_RS16010
	HIRAN domain-containing protein	GPK32_RS16015
	Phytoene desaturase family protein	GPK32_RS16135
	AI-2E family transporter	GPK32_RS16380
	Gp58-like family protein	GPK32_RS16395
	Inositol monophosphatase family protein	GPK32_RS16400
	NUDIX hydrolase N-terminal domain-containing protein	GPK32_RS16405
	GtrA family protein	GPK32_RS16410
	SDR family oxidoreductase	GPK32_RS16425
	ABC transporter permease	GPK32_RS16445
	Helix-turn-helix domain-containing protein	GPK32_RS16460
	MFS transporter	GPK32_RS16490
	Phosphatidylglycerophosphatase A	LTG66_RS09395
	MarR family winged helix-turn-helix transcriptional regulator	LTG66_RS09910
	Spx/MgsR family RNA polymerase-binding regulatory protein	LTG66_RS10795
BF_15	Hypothetical protein	LTG66_RS00190
	SGNH/GDSL hydrolase family protein	LTG66_RS00240
	LysR family transcriptional regulator	LTG66_RS00795
	GAF domain-containing protein	LTG66_RS01245
	Hypothetical protein	LTG66_RS01270
	Type II-A CRISPR-associated protein Csn2	LTG66_RS02430
	Molybdenum cofactor guanylyltransferase	LTG66_RS03325
	Folylpolyglutamate synthase/dihydrofolate synthase family protein	LTG66_RS03375
	A/G-specific adenine glycosylase	LTG66_RS03650
	Cyclic pyranopterin monophosphate synthase MoaC	LTG66_RS03830
	Nuclear transport factor 2 family protein	LTG66_RS03835
	Molybdopterin-guanine dinucleotide biosynthesis protein B	LTG66_RS04940
	Molybdopterin molybdotransferase MoeA	LTG66_RS04945
	MogA/MoaB family molybdenum cofactor biosynthesis protein	LTG66_RS04950
	MobA/MobL family protein	LTG66_RS05535
	HesA/MoeB/ThiF family protein	LTG66_RS08450
	DUF3775 domain-containing protein	LTG66_RS08480

Strain	Product	Gene Locus
	DUF4428 domain-containing protein	LTG66_RS08580
	DUF4430 domain-containing protein	LTG66_RS08585
	DUF4868 domain-containing protein	LTG66_RS08600
	DUF536 domain-containing protein	LTG66_RS08625
	DUF5067 domain-containing protein	LTG66_RS08630
	DUF536 domain-containing protein	LTG66_RS08640
	DUF5655 domain-containing protein	LTG66_RS08645
	DUF771 domain-containing protein	LTG66_RS08655
	DUF853 family protein	LTG66_RS08665
	DUF927 domain-containing protein	LTG66_RS08670
	DUF975 family protein	LTG66_RS08675
	ECF transporter S component	LTG66_RS08680
	Endodeoxyribonuclease	LTG66_RS08695
	Hypothetical protein	LTG66_RS08715
	Energy-coupling factor transporter transmembrane protein EcfT	LTG66_RS08720
	Enolase C-terminal domain-like protein	LTG66_RS08725
	Gluconate:H+ symporter	LTG66_RS10240
	Nitrate reductase subunit beta	LTG66_RS10250
	DUF87 domain-containing protein	LTG66_RS10390
	Putative glycosyltransferase, exosortase G system-associated	LTG66_RS10560
	Type II CRISPR RNA-guided endonuclease Cas9	LTG66_RS10565
	Nitrate reductase molybdenum cofactor assembly chaperone	LTG66_RS10625
	RNase III inhibitor	LTG66_RS11015
	Hypothetical protein	LTG66_RS11540
	HlyD family secretion protein	LTG66_RS12895
	Helix-turn-helix domain-containing protein	LTG66_RS12955
	TetR/AcrR family transcriptional regulator	LTG66_RS12960
	AraC family transcriptional regulator	LTG66_RS13095
	GNAT family N-acetyltransferase	LTG66_RS13200
	Metal ABC transporter ATP-binding protein	LTG66_RS13545
	Cyclic lactone autoinducer peptide	LTG66_RS13720
	EAL domain-containing protein	LTG66_RS14795
	FAD-binding protein	LTG66_RS15130
	ClC family H(+)/Cl(-) exchange transporter	LTG66_RS15465
	Glycine betaine/L-proline ABC transporter ATP-binding protein	LTG66_RS15485
	Respiratory nitrate reductase subunit gamma	LTG66_RS15505
	Hypothetical protein	LTG66_RS15510
	Hypothetical protein	LTG66_RS15515
	Thioredoxin-disulfide reductase	LTG66_RS15520
	Thioredoxin family protein	LTG66_RS15525
	ArsR family transcriptional regulator	LTG66_RS15530
	Hypothetical protein	LTG66_RS15535
	PTS system mannose/fructose/sorbose family transporter subunit IID	LTG66_RS15540
	GHKL domain-containing protein	LTG66_RS15565
	NFACT RNA binding domain-containing protein	LTG66_RS15630
FLPL05	Cold-shock protein	GJS00_RS00505
	Serine hydrolase	GJS00_RS00760
	Pre-toxin TG domain-containing protein	GJS00_RS00770
	PTS fructose transporter subunit IIA	GJS00_RS03280
	PTS glucose transporter subunit IIA	GJS00_RS04800
	PTS fructose transporter subunit IIC	GJS00_RS04810
	PTS glucose transporter subunit IIA	GJS00_RS04915
	Glycosyltransferase family 2 protein	GJS00_RS04975
	Metallophosphoesterase	GJS00_RS05875
	Recombinase family protein	GJS00_RS07090
	Hypothetical protein	GJS00_RS07095
	16S rRNA (adenine(1518)-N(6)/adenine(1519)-N(6))- dimethyltransferase RsmA	GJS00_RS07110

Strain	Product	Gene Locus
	30S ribosomal protein S13	GJS00_RS07130
	GntR family transcriptional regulator	GJS00_RS07145
	Cof-type HAD-IIB family hydrolase	GJS00_RS07170
	Pentapeptide repeat-containing protein	GJS00_RS07175
	Glycosyltransferase	GJS00_RS07180
	Nucleoside phosphorylase	GJS00_RS07185
	Hypothetical protein	GJS00_RS07195
	PTS N-acetylglucosamine transporter subunit IIBC	GJS00_RS07200
	Helix-turn-helix transcriptional regulator	GJS00_RS07210
	PTS sugar transporter subunit IIC	GJS00_RS07220
	Branched-chain amino acid transport system II carrier protein	GJS00_RS07225
	3-deoxy-7-phosphoheptulonate synthase	GJS00_RS07330
	Hypothetical protein	GJS00_RS07340
	Histidinol dehydrogenase	GJS00_RS07350
	Metal-sensitive transcriptional regulator	GJS00_RS07360
	Histidinol-phosphate transaminase	GJS00_RS07365
	PTS transporter subunit EIIC	GJS00_RS07535
	Putative frv operon regulatory protein	GJS00_RS08745
	Hypothetical protein	GJS00_RS08840
	Hypothetical protein	GJS00_RS08945
	Hypothetical protein	GJS00_RS08950
	ROK family protein	GJS00_RS09055
	Glycosyltransferase	GJS00_RS09065
	Glycosyltransferase	GJS00_RS09070
	SH3 domain-containing protein	GJS00_RS09075
	Barstar family protein	GJS00_RS09080
	DUF1801 domain-containing protein	GJS00_RS09105
	Glycerol-3-phosphate cytidylyltransferase	GJS00_RS09110
	Helix-turn-helix domain-containing protein	GJS00_RS09115
	Bacterial Ig-like domain-containing protein	GJS00_RS09125
	Endonuclease MutS2	GJS00_RS09135
	TetR/AcrR family transcriptional regulator	GJS00_RS09145
	YfhO family protein	GJS00_RS09150
	AraC family transcriptional regulator	GJS00_RS09155
	ISL3-like element ISP1 family transposase	GJS00_RS09160
	Tetratricopeptide repeat protein	GJS00_RS09170
	Sugar phosphate isomerase/epimerase	GJS00_RS09175
	Cadmium resistance transporter	GJS00_RS09200
	Mevalonate kinase	GJS00_RS09555
	Sugar-phosphatase	GJS00_RS12295
	Hypothetical protein	GJS00_RS13355
	ABC transporter ATP-binding protein	GJS00_RS13370
	Iron ABC transporter permease	GJS00_RS13380
	Amino acid permease	GJS00_RS13400
	Pur operon repressor	GJS00_RS13405
	50S ribosomal protein L1	GJS00_RS13410
	TRNA (adenosine(37)-N6)-threonylcarbamoyltransferase complex ATPase subunit type 1 TsaE	GJS00_RS13415
	FtsW/RodA/SpoVE family cell cycle protein	GJS00_RS13420
	NAD(P)/FAD-dependent oxidoreductase	GJS00_RS13435
	Hypothetical protein	GJS00_RS13445
	Flavodoxin	GJS00_RS13450
	Serine hydrolase	GJS00_RS13455
	Molybdenum cofactor biosynthesis protein MoaE	GJS00_RS13460
	MoaD/ThiS family protein	GJS00_RS13465
	PRD domain-containing protein	GJS00_RS14585
LPT52	IS1182 family transposase	L0056_RS00870
	Galactokinase	L0056_RS01655

Strain	Product	Gene Locus
	Zinc-dependent alcohol dehydrogenase family protein	L0056_RS01720
	MFS transporter	L0056_RS01985
	Bacterial Ig-like domain-containing protein	L0056_RS02410
	Tail protein	L0056_RS02420
	LLM class flavin-dependent oxidoreductase	L0056_RS02425
	YhgE/Pip family protein	L0056_RS02430
	Cupin domain-containing protein	L0056_RS02435
	ABC transporter ATP-binding protein	L0056_RS02440
	Glycosyltransferase	L0056_RS02445
	Phosphate ABC transporter permease subunit PstC	L0056_RS02450
	Phage tail family protein	L0056_RS02455
	Carboxymuconolactone decarboxylase family protein	L0056_RS02460
	ABC transporter ATP-binding protein	L0056_RS02465
	LysM domain-containing protein	L0056_RS02470
	DUF2075 domain-containing protein	L0056_RS02475
	Phage tail protein	L0056_RS02480
	Helix-turn-helix transcriptional regulator	L0056_RS02485
	LysM domain-containing protein	L0056_RS02490
	Helix-turn-helix transcriptional regulator	L0056_RS02495
	Hypothetical protein	L0056_RS02500
	Hypothetical protein	L0056_RS02505
	FMN-binding protein	L0056_RS02510
	S41 family peptidase	L0056_RS02520
	MFS transporter	L0056_RS02525
	Zinc-binding alcohol dehydrogenase family protein	L0056_RS02535
	LysR family transcriptional regulator	L0056_RS02540
	M57 family metalloprotease	L0056_RS02545
	TMEM175 family protein	L0056_RS02550
	DNA replication protein DnaD	L0056_RS02555
	DUF4811 domain-containing protein	L0056_RS02560
	PTS glucitol/sorbitol transporter subunit IIA	L0056_RS02565
	Hypothetical protein	L0056_RS02570
	Cof-type HAD-IIB family hydrolase	L0056_RS02575
	Glucose-1-phosphate adenylyltransferase	L0056_RS02585
	HAD family hydrolase	L0056_RS02590
	Aspartate ammonia-lyase	L0056_RS02595
	AEC family transporter	L0056_RS02600
	Membrane protein	L0056_RS02605
	Nitroreductase family protein	L0056_RS02610
	HAMP domain-containing sensor histidine kinase	L0056_RS02615
	Cation:proton antiporter	L0056_RS02620
	ASCH domain-containing protein	L0056_RS02625
	Trypsin-like peptidase domain-containing protein	L0056_RS02630
	Phosphopyruvate hydratase	L0056_RS02635
	TetR/AcrR family transcriptional regulator	L0056_RS02640
	Nucleoside hydrolase	L0056_RS02645
	Phage holin family protein	L0056_RS03040
	Ribosomal protein S18-alanine N-acetyltransferase	L0056_RS05040
	IS3 family transposase	L0056_RS05065
	Putative holin-like toxin	L0056_RS05075
	Hypothetical protein	L0056_RS05080
	Hydroxyethylthiazole kinase	L0056_RS05085
	Hypothetical protein	L0056_RS05095
	DUF111 family protein	L0056_RS05100
	Phosphoglucosamine mutase	L0056_RS05105
	Hypothetical protein	L0056_RS05145
	Hypothetical protein	L0056_RS05150
	Recombinase family protein	L0056_RS05155

Strain	Product	Gene Locus
	IS3 family transposase	L0056_RS05160
	IS3 family transposase	L0056_RS05170
	Alpha-glucosidase	L0056_RS05175
	Hypothetical protein	L0056_RS05180
	SIS domain-containing protein	L0056_RS09160
	Hypothetical protein	L0056_RS10420
	KxYKxGKxW signal peptide domain-containing protein	L0056_RS10425
	Hypothetical protein	L0056_RS10440
	Hypothetical protein	L0056_RS10445
	NADPH-dependent F420 reductase	L0056_RS10450
	Pyruvate dehydrogenase (acetyl-transferring) E1 component subunit alpha	L0056_RS10505
	Hypothetical protein	L0056_RS10520
	LacI family DNA-binding transcriptional regulator	L0056_RS10525
	LytTR family transcriptional regulator	L0056_RS10530
	Sugar-binding transcriptional regulator	L0056_RS10535
	Hypothetical protein	L0056_RS10550
	Membrane protein	L0056_RS10565
	MupG family TIM beta-alpha barrel fold protein	L0056_RS10570
	MurR/RpiR family transcriptional regulator	L0056_RS10575
	N-acetylmuramic acid 6-phosphate etherase	L0056_RS10580
	Transcriptional regulator	L0056_RS10605
	NADP-dependent oxidoreductase	L0056_RS10655
	PatB family C-S lyase	L0056_RS10835
	Glycoside hydrolase family 31 protein	L0056_RS10980
	Catalase	L0056_RS13060
	UDP-galactopyranose mutase	L0056_RS13115
	Sugar ABC transporter	L0056_RS14945
	SemiSWEET family transporter	L0056_RS14950
	GntR family transcriptional regulator	L0056_RS14955
	LPXTG cell wall anchor domain-containing protein	L0056_RS14960
	PTS sugar transporter subunit IIC	L0056_RS14965
	GH25 family lysozyme	L0056_RS14970
	GIY-YIG nuclease family protein	L0056_RS14975
	GNAT family N-acetyltransferase	L0056_RS14980
	YjjG family noncanonical pyrimidine nucleotidase	L0056_RS14985
	Hypothetical protein	L0056_RS14990
	NAD(P)-binding domain-containing protein	L0056_RS14995
	Hypothetical protein	L0056_RS15000
	Accessory gene regulator AgrB	L0056_RS15005
	Glucosamine-6-phosphate deaminase	L0056_RS15010
	SDR family oxidoreductase	L0056_RS15025
	Hypothetical protein	L0056_RS15030
	ABC transporter ATP-binding protein	L0056_RS15035
	Acyltransferase family protein	L0056_RS15040
	Hypothetical protein	L0056_RS15045
	OsmC family protein	L0056_RS15050
LZ95	AraC family transcriptional regulator	AD081_RS05455
	LCP family protein	AD081_RS05485
	Hypothetical protein	AD081_RS05505
	PTS glucose transporter subunit IIABC	AD081_RS05510
	Bifunctional UDP-N-acetylglucosamine diphosphorylase/glucosamine-1-phosphate N-acetyltransferase GlmU	AD081_RS05515
	Stealth CR1 domain-containing protein	AD081_RS05520
	Flavodoxin	AD081_RS05525
	Accessory Sec system glycosyltransferase Asp1	AD081_RS05530
	Hypothetical protein	AD081_RS05535
	YlbG family protein	AD081_RS05540
	Hypothetical protein	AD081_RS05575

Strain	Product	Gene Locus
	Hypothetical protein	AD081_RS05580
	Imidazoleglycerol-phosphate dehydratase HisB	AD081_RS05585
	Glycogen synthase GlgA	AD081_RS05600
	Family 1 glycosylhydrolase	AD081_RS05605
	Hypothetical protein	AD081_RS05610
	Helix-turn-helix transcriptional regulator	AD081_RS05615
	Hypothetical protein	AD081_RS05620
	DNA gyrase subunit A	AD081_RS05625
	Hypothetical protein	AD081_RS05630
	Hypothetical protein	AD081_RS05635
	Hypothetical protein	AD081_RS05640
	EAL domain-containing protein	AD081_RS05645
	Hypothetical protein	AD081_RS05650
	Hypothetical protein	AD081_RS05660
	TetR/AcrR family transcriptional regulator	AD081_RS05665
	Hypothetical protein	AD081_RS05675
	Phage regulatory protein/antirepressor Ant	AD081_RS05770
	DUF1054 domain-containing protein	AD081_RS05775
	Hypothetical protein	AD081_RS05910
	Hypothetical protein	AD081_RS05915
	Hypothetical protein	AD081_RS05920
	Hypothetical protein	AD081_RS05925
	Hypothetical protein	AD081_RS05930
	Metalloregulator ArsR/SmtB family transcription factor	AD081_RS07580
	GtrA family protein	AD081_RS07605
	Alpha/beta fold hydrolase	AD081_RS08820
	Hypothetical protein	AD081_RS09095
	DUF488 family protein	AD081_RS12295
	Carboxymuconolactone decarboxylase family protein	AD081_RS13980
	SLC13 family permease	AD081_RS14930
	Class II fructose-1,6-bisphosphate aldolase	AD081_RS15370
	Hypothetical protein	AD081_RS15375
	Transketolase	AD081_RS15405
	Hypothetical protein	AD081_RS15430
	HAD family hydrolase	AD081_RS15435
	D-aminoacyl-tRNA deacylase	AD081_RS15440
	Hypothetical protein	AD081_RS15450
	Bifunctional (p)ppGpp synthetase/guanosine-3,5-bis(diphosphate) 3-pyrophosphohydrolase	AD081_RS15455
	30S ribosomal protein S12	AD081_RS15465
	GNAT family N-acetyltransferase	AD081_RS15470
	Sodium:proton antiporter	AD081_RS15480
	Endodeoxyribonuclease	AD081_RS15735
	DUF916 domain-containing protein	AD081_RS15750
	NAD(P)H-binding protein	AD081_RS15755
	2-oxo acid dehydrogenase subunit E2	AD081_RS15820
	MerR family transcriptional regulator	AD081_RS16135
	YkuJ family protein	AD081_RS16265
	Hypothetical protein	AD081_RS16350
	CPBP family intramembrane metalloprotease	AD081_RS16375
	Hypothetical protein	AD081_RS16380
	OsmC family protein	AD081_RS16415
	Hypothetical protein	AD081_RS16465
	50S ribosomal protein L11 methyltransferase	AD081_RS16480
	IS30 family transposase	AD081_RS16485

Table S5. Status of gene retention involved in strain-specific Molybdopterin biosynthesis from 25 *Lb. plantarum* genomes

Product	GTP 3,8-cyclase MoaA	Response regulator transcription factor	Sensor histidine kinase
Gene	*moaA*	*nreC*	*nreB*
CACC 558	GWD03_RS04050	GWD03_RS04085	GWD03_RS04090
E2	-	-	-
FBL-3a	EEJ46_RS06195	EEJ46_RS06160	EEJ46_RS06155
SRCM210465	-	-	-
W2	L1599_RS05965	L1599_RS06000	L1599_RS06005
10CH	B0667_RS06515	B0667_RS06550	B0667_RS06555
8P-A3	-	-	-
LL441	O4Z47_RS06330	O4Z47_RS06365	O4Z47_RS06370
NCIMB 700965	CO218_RS13915	CO218_RS13950	CO218_RS13955
Q7	B1H25_RS08675	B1H25_RS08640	B1H25_RS08635
B21	SH83_RS06275	SH83_RS06310	SH83_RS06315
KACC 92189	COO33_RS01090	COO33_RS01055	COO33_RS01050
KM2	JQC82_RS06350	JQC82_RS06385	JQC82_RS06390
MF1298	-	-	-
RI-113	-	-	-
BLS41	BOQ65_RS00545	BOQ65_RS00580	BOQ65_RS00585
DSR_M2	-	-	-
IDCC3501	-	-	-
KC28	-	-	-
LMT1-48	-	-	-
83-18	GPK32_RS07385	GPK32_RS07420	GPK32_RS07425
BF_15	-	-	-
FLPL05	GJS00_RS06130	GJS00_RS06165	GJS00_RS06170
LPT52	L0056_RS06310	L0056_RS06345	L0056_RS06350
LZ95	-	-	-
